# NeoHunter: Flexible software for systematically detecting neoantigens from sequencing data

**DOI:** 10.1002/qub2.28

**Published:** 2024-01-22

**Authors:** Tianxing Ma, Zetong Zhao, Haochen Li, Lei Wei, Xuegong Zhang

**Affiliations:** ^1^ MOE Key Lab of Bioinformatics, Bioinformatics Division of BNRIST and Department of Automation Tsinghua University Beijing China; ^2^ School of Medicine Tsinghua University Beijing China; ^3^ School of Life Sciences Tsinghua University Beijing China

**Keywords:** cancer vaccine, molecular alteration, neoantigen, neoantigen prioritization

## Abstract

Complicated molecular alterations in tumors generate various mutant peptides. Some of these mutant peptides can be presented to the cell surface and then elicit immune responses, and such mutant peptides are called neoantigens. Accurate detection of neoantigens could help to design personalized cancer vaccines. Although some computational frameworks for neoantigen detection have been proposed, most of them can only detect SNV‐ and indel‐derived neoantigens. In addition, current frameworks adopt oversimplified neoantigen prioritization strategies. These factors hinder the comprehensive and effective detection of neoantigens. We developed NeoHunter, flexible software to systematically detect and prioritize neoantigens from sequencing data in different formats. NeoHunter can detect not only SNV‐ and indel‐derived neoantigens but also gene fusion‐ and aberrant splicing‐derived neoantigens. NeoHunter supports both direct and indirect immunogenicity evaluation strategies to prioritize candidate neoantigens. These strategies utilize binding characteristics, existing biological big data, and T‐cell receptor specificity to ensure accurate detection and prioritization. We applied NeoHunter to the TESLA dataset, cohorts of melanoma and non‐small cell lung cancer patients. NeoHunter achieved high performance across the TESLA cancer patients and detected 79% (27 out of 34) of validated neoantigens in total. SNV‐ and indel‐derived neoantigens accounted for 90% of the top 100 candidate neoantigens while neoantigens from aberrant splicing accounted for 9%. Gene fusion‐derived neoantigens were detected in one patient. NeoHunter is a powerful tool to ‘catch all’ neoantigens and is available for free academic use on Github (XuegongLab/NeoHunter).

## 1 INTRODUCTION

Various molecular alterations occur in cancers, many of which lead to aberrant proteins that can drive cancer progression and influence clinical outcomes [1]. Some mutant peptides derived from these molecular alterations in cancers can be presented to the cell surface through human leukocyte antigens (HLAs), which are major histocompatibility complexes (MHCs) in humans. Peptides may bind with HLAs to form pMHC complexes on the cell surface, and some of these pMHC complexes can be recognized by T‐cell receptors (TCRs), eliciting immune responses to kill cancer cells. These mutant peptides, which can be presented by HLAs and further recognized by TCRs to elicit immune responses, are named neoantigens. Neoantigens could be utilized to develop personalized cancer vaccines, and pioneering studies have shown that such immunotherapy is a promising cancer treatment approach [2, 3, 4].

Precise and efficient neoantigen detection is crucial to personalized cancer vaccine development. Some relevant computational frameworks have been presented recently [5, 6]. For instance, pTuneos [5] detects single nucleotide variants (SNVs) and indels from next‐generation sequencing (NGS) data and predicts possible neoantigens based on features such as the dissimilarity between the wild‐type and mutant peptides. However, SNVs and indels are only partial sources of neoantigens in cancers. Many recent studies highlight the key role of gene fusions and aberrant splicing in neoantigen generation and cancer vaccine design [2, 7, 8, 9, 10, 11, 12, 13, 14, 15, 16, 17, 18]. Such findings indicate that a comprehensive discovery of neoantigens, rather than only detecting SNV‐ and indel‐derived neoantigens alone, is necessary. In addition, previous neoantigen prediction approaches mainly employed the sequence dissimilarity between wild‐type and mutant peptides or the expression levels of mutant peptides to evaluate the possibility of peptides being neoantigens. However, the immunogenicity of T‐cell recognition is not considered in these approaches, leading to a reduction in the accuracy and robustness of neoantigen detection and prioritization. Recently, rapid accumulation of clinical data and evolving deep‐learning‐based models empower TCR–pMHC interaction estimation [19, 20], making it possible to evaluate immunogenicity by comparing the query peptide with confirmed antigens or even directly predict TCR specificity in a more confident and accurate way. Accordingly, software capturing all significant sources of neoantigens and applying advanced neoantigen prioritization approaches to ensure high performance of neoantigen detection are urgently needed.

We developed NeoHunter, the first software that can systematically ‘catch all’ neoantigens from NGS data. It can detect neoantigens generated from various molecular alterations, including SNVs, indels, gene fusions, and aberrant splicing. NeoHunter integrates various features to discover candidate neoantigens, such as the expression level of aberrant transcripts as well as the binding affinity and binding stability between peptides and HLA alleles. NeoHunter supports both indirect and direct immunogenicity evaluation strategies to evaluate T‐cell recognition on peptides and thus further prioritize candidate neoantigens. In the indirect evaluation strategy, NeoHunter compares the candidate peptide with confirmed antigens to estimate the possibility of the peptide being recognized by T cells. In the direct evaluation strategy, NeoHunter decodes TCR components and utilizes the state‐of‐the‐art TCR–pMHC interaction prediction deep learning model to estimate TCR specificity. NeoHunter accepts a variety of data input formats, including raw sequencing reads, aligned sequences, and variant calls of DNA sequencing data. We reviewed the functions and characteristics of existing neoantigen detection methods and compared NeoHunter to them in Supplementary Table S1.

We applied NeoHunter to five cancer patients collected from Tumor Neoantigen Selection Alliance (TESLA) [21]. TESLA validated some neoantigen candidates in vitro and confirmed 34 candidates to be immunogenic (true neoantigens). NeoHunter detected 27 validated neoantigens and achieved high performance among several evaluation metrics compared with existing neoantigen detection methods. Among the top 100 candidate neoantigens of each patient, 9% were derived from aberrant splicing, and one gene fusion‐derived neoantigen was detected in a patient, suggesting that it is necessary to consider molecular alterations in addition to SNVs and indels. In addition, the results showed that the indirect immunogenicity evaluation and prioritization strategy performed better than the direct strategy. All the results indicated that NeoHunter can serve as a comprehensive and effective neoantigen detection tool to advance research on personalized cancer vaccine development.

## 2 RESULTS

### 2.1 Overview of NeoHunter

**FIGURE 1:**
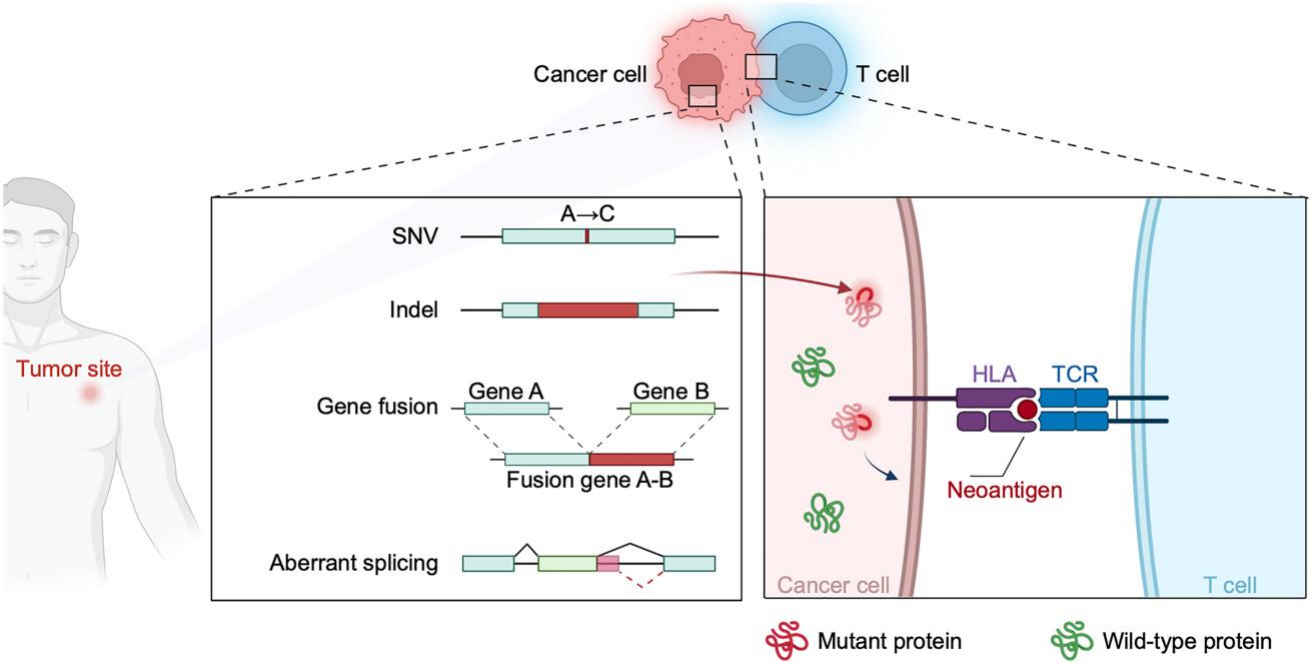
Conceptual diagram of NeoHunter and neoantigen‐induced immune responses. The left panel shows that multiple molecular alterations, including single nucleotide variants (SNVs), indels, gene fusions, and aberrant splicing in cancer cells generate mutant proteins. The right panel shows that some mutant proteins can be presented to the cell surface through human leukocyte antigen (HLA) and form pMHC (peptide–MHC) complexes. Some pMHCs can further be recognized by the T‐cell receptor (TCR) and elicit immune responses. The peptides of such pMHCs are neoantigens. The design philosophy of NeoHunter is consistent with the biological process described above. NeoHunter consists of three modules, that is, molecular alteration detection and HLA allele typing from sequencing data, peptide–MHC binding prediction, immunogenicity evaluation and neoantigen prioritization.

The philosophy of NeoHunter is consistent with the biological process of neoantigen‐induced immune responses (Figure 1). First, NeoHunter detects different types of molecular alterations, including SNVs, indels, gene fusions, and aberrant splicing, from NGS data of cancer patients. Meanwhile, NeoHunter decodes sequencing reads that fall in the HLA genomic region and in silico analyzes the types of HLA alleles. Then, NeoHunter estimates the binding affinity and binding stability between mutant peptides and HLA alleles. A peptide–HLA pair estimated to be in strong binding indicates that the peptide has great potential to be presented by the HLA allele and form pMHC complexes. Such mutant peptides are considered as candidate neoantigens. Finally, NeoHunter evaluates the immunogenicity of TCR recognition on pMHC complexes and prioritizes candidate neoantigens. It can indirectly evaluate immunogenicity by comparing the similarity between the query peptide and confirmed antigens (default strategy). It can also directly evaluate immunogenicity by deep‐learning‐based TCR–pMHC interaction prediction models. Related evaluation scores are used to prioritize the candidate neoantigens.

**FIGURE 2:**
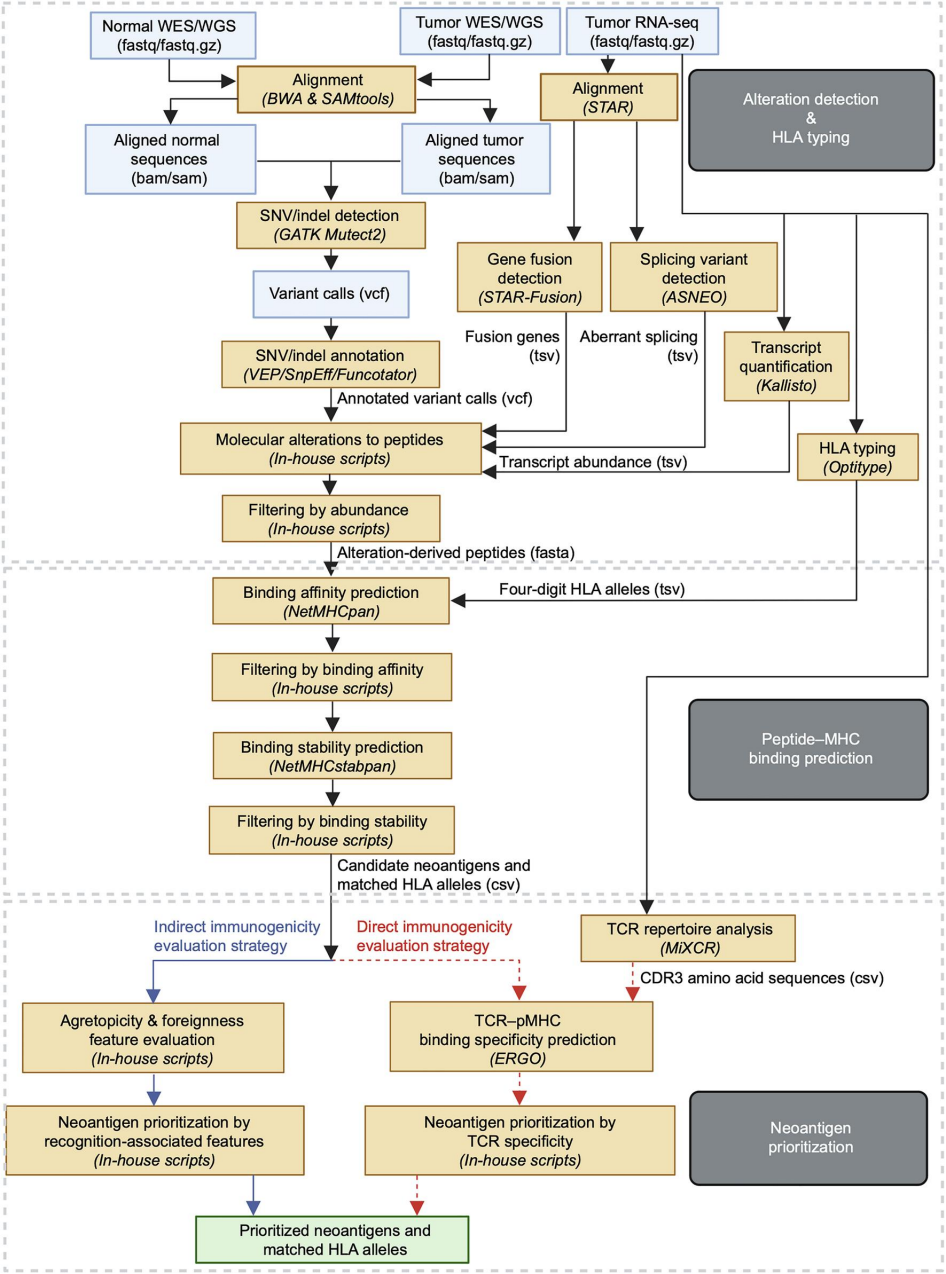
Construction of NeoHunter. NeoHunter consists of three modules. (1) Module 1 (top panel): alteration detection and human leukocyte antigen typing. NeoHunter flexibly accepts multiple kinds of sequencing data as inputs. Acceptable formats of sequencing data are outlined with blue boxes. For single nucleotide variant/indel detection, users could supply either raw reads (fastq‐formatted files), aligned sequences (bam‐formatted files), or variant calls (vcf‐formatted files) of tumor and normal samples as inputs. For gene fusion and aberrant splicing detection and HLA typing, RNA‐seq data (fastq‐formatted files) of tumor samples are taken as inputs. This module outputs molecular alterations in cancers, their downstream peptides, and four‐digit HLA alleles. (2) Module 2 (middle panel): peptide‐MHC binding prediction. This module takes alteration‐derived peptides and HLA alleles as inputs. It adopts some deep‐learning‐based models to predict the binding affinity and binding stability between each peptide and HLA allele. Then, the module considered peptide–HLA pairs with strong binding interactions as candidates. This module outputs candidate neoantigens and matched HLA alleles. (3) Module 3 (bottom panel): neoantigen prioritization. NeoHunter further evaluates the immunogenicity of T‐cell receptor (TCR) recognition and prioritizes candidate neoantigens with related immunogenicity scores. This module supports two different evaluation and prioritization strategies. The blue arrows illustrate the indirect immunogenicity evaluation strategy, in which the module calculates recognition‐associated features by estimating similarity between the given peptide and previously confirmed TCR‐binding peptides and dissimilarity between the given mutant peptide and its wild‐type. The module utilizes these features to prioritize candidate neoantigens. This indirect immunogenicity evaluation strategy was set as the default. The red dashed arrows illustrate the direct immunogenicity evaluation strategy, in which the module infers TCR clones and estimates TCR specificity on recognizing peptides. The estimated TCR specificity scores are utilized to prioritize candidate neoantigens. Finally, this module outputs prioritized neoantigens and matched HLA alleles.

The above three processes are constructed into three modules, that is, alteration detection and HLA typing, peptide–MHC binding prediction, and neoantigen prioritization. Constructions of the NeoHunter are shown in Figure 2. Detailed construction methods can be found in Materials and Methods.

### 2.2 NeoHunter achieves high performance on TESLA data

**FIGURE 3:**
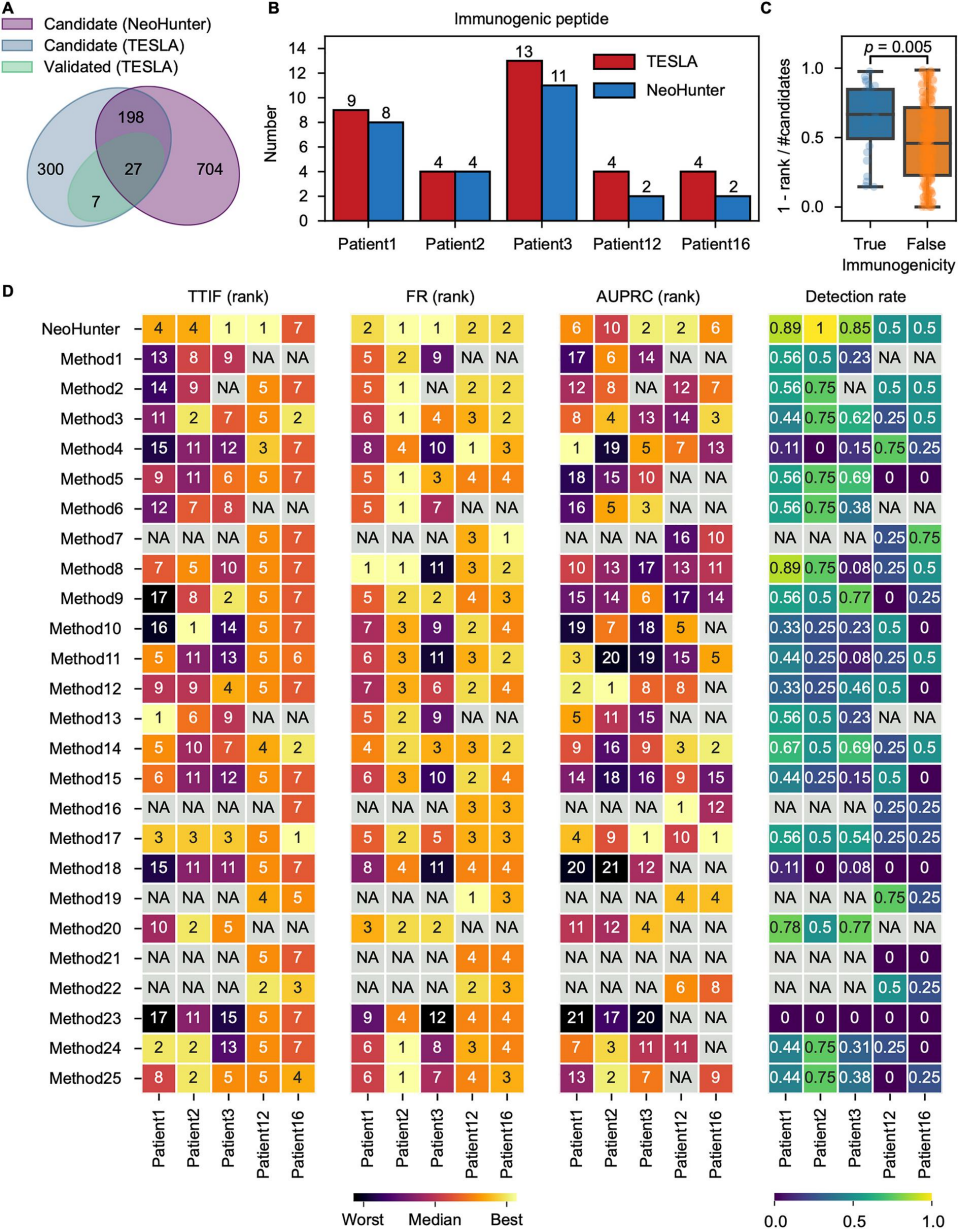
Performance of NeoHunter on TESLA data. (A) A Venn diagram summarizing the overlap between candidate peptides tested/reported by TESLA and NeoHunter and the overlap between immunogenic peptides validated by TESLA. (B) Bar plots of the number of immunogenic peptides reported by NeoHunter or validated by TESLA for each patient. (C) Box plots of the relative ranking score for validated immunogenic peptides and tested non‐immunogenic peptides. The relative ranking score is one minus the rank of the peptide divided by the number of candidate peptides in the matched patient (*t*‐test, *p* = 0.005). (D) Rank of each method on the three metrics (the left three panels): TTIF (top twenty immunogenic fraction), FR (fraction ranked), AUPRC (the area under the precision recall curve), and the detection rate (the right panel). Each row corresponds to a neoantigen detection method, and each column represents one patient. NA indicates that the corresponding contents were not applicable (indicated by gray squares).

We applied NeoHunter to a benchmark dataset collected from the TESLA [21]. These TESLA cancer data included three melanoma patients (named patient1, patient2, and patient3) and two non‐small cell lung cancer (NSCLC) patients (named patient12 and patient16). The TESLA consortium supplied whole exome sequencing (WES) data of both tumor and normal tissues, and RNA‐seq data of tumor tissues of cancer patients. Detailed information on the five T cancer patients is described in Supplementary Table S2. Twenty‐five independent teams performed neoantigen detection on these data with their own neoantigen detection methods. The TESLA consortium collected results from these teams and then selected the overlapping parts of the results as candidate neoantigens. The TESLA consortium then synthesized peptides of these candidates and validated the binding of the peptides to existing T cells of patients in vitro to confirm immunogenicity. TESLA tested 532 candidates and validated 34 candidates to be immunogenic (true neoantigens) (Figure 3A, related results for each patient shown in Supplementary Figure S1).

Since the teams participating in TESLA only considered SNV‐ and indel‐derived neoantigens, all candidates tested by the TESLA consortium were derived from SNVs and indels. We thus first detected SNV‐ and indel‐derived neoantigens only to maintain the consistency of performance evaluation. We detected 929 candidate neoantigens from the five patients, and 225 of them were also candidates in TESLA. Among these 225 tested candidates, 27 candidates were validated to be immunogenic, that is, true neoantigens (Figure 3A, related results for each patient can be found in Supplementary Figure S1). NeoHunter detected 84.62% (median across the five cancer patients) of the validated immunogenic peptides (Figure 3B). In addition, we found that the 27 immunogenic peptides had higher ranks than the remaining 198 non‐immunogenic peptides (the peptides tested by TESLA but not validated as immunogenic) (Figure 3C, *p* = 0.005, *t*‐test). Such findings indicated that NeoHunter had strong power to detect neoantigens, and candidate neoantigens with higher priority had higher confidence.

We further compared the performance of NeoHunter with the 25 methods from the TESLA participants. We adopted four evaluation metrics to evaluate the performance: (1) TTIF, top twenty immunogenic fraction, which is the fraction of validated immunogenic peptides in the top 20 candidate peptides; (2) FR, fraction ranked, which measures the fraction of validated immunogenic peptides of the top 100 candidates against all immunogenic peptides from TESLA; (3) AUPRC, the area under the precision recall curve, which reflects the discriminant ability on immunogenic and non‐immunogenic peptides; and (4) detection rate, which represents the proportion of immunogenic peptides of candidates against all immunogenic peptides from TESLA. Details of these metrics are described in Materials and Methods. NeoHunter achieved consistent outstanding performance across these metrics over the 25 methods (Figure 3D). NeoHunter achieved superior performance on fraction ranked (FR). It performed best on two patients and performed the second best on the other three patients compared to the other methods. NeoHunter also achieved high performance on TTIF. It performed best on two patients, and the performance on the other patients also ranked high among the methods. NeoHunter tended to balance precision and recall and achieved moderate AUPRC on some patients. This ensured that the true neoantigens could be effectively detected. For instance, although AUPRC on patient2 ranked 10 among the 26 methods, the detection rate of immunogenic peptides on this patient by NeoHunter was 100%. NeoHunter was the only method to completely detect immunogenic peptides from this patient among the 26 methods.

### 2.3 NeoHunter can detect neoantigens derived from SNVs, indels, gene fusions, and aberrant splicing

**FIGURE 4:**
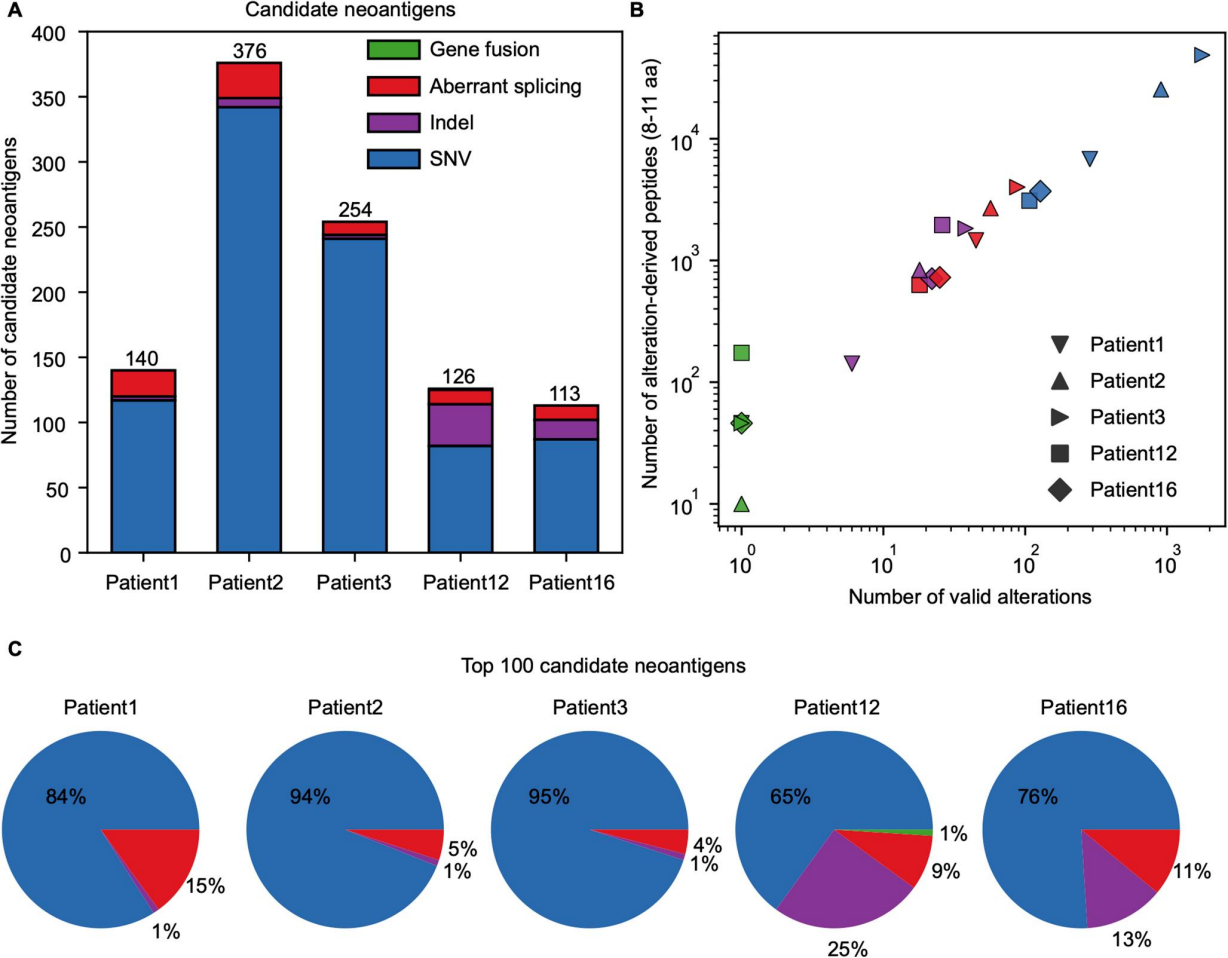
Results of neoantigens derived from different types of alterations. (A) Number of candidate neoantigens of each patient. Colors in each bar represent types of alterations. (B) Scatter plots showing the number of valid alterations (*x*‐axis) and number of alteration‐derived peptides (*y*‐axis). Colors and shapes of the dots indicate types of alterations and patient, respectively. (C) Pie charts showing the frequency of each type of alteration in the top 100 candidate neoantigens of each patient. The colors represent the types of alterations. aa, amino acids.

We further detected neoantigens derived from SNVs, indels, gene fusions, and aberrant splicing with NeoHunter. We detected 107 (in patient12) to 1773 (in patient3) valid SNVs, generating 3100 to 48,618 peptides (with lengths from 8 to 11 amino acids (aa), the same below) from the five TESLA patients. There were 6 (in patient1) to 38 (in patient3) valid indels in these patients, generating 142–1832 peptides. We detected 18 (in patient12) to 88 (in patient3) valid aberrant splicing that generated 631–4002 peptides. We detected one valid gene fusion in each patient, and these fusion genes generated 10 (in patient2) to 174 (in patient12) peptides. The number of detected valid alterations and generated peptides of each patient are shown in Figure 4B and Supplementary Table S3. Detailed methods of alteration detection and filtering as well as alteration‐derived peptide generation are described in Materials and Methods.

NeoHunter predicted binding affinity and binding stability between the alteration‐derived peptides and HLA alleles, and selected pMHCs with strong binding interactions as candidates. The candidates from all four alterations were merged together and then prioritized. NeoHunter reported 113–376 candidate neoantigens in the five TESLA patients (Figure 4A). As shown in Figure 4C, in the top 100 candidate neoantigens of each patient, SNV‐ and indel‐derived neoantigens ranged from 85% to 96% (median was 90%). The two NSCLC patients (patient12 and patient16) had a higher frequency of indel‐derived neoantigens than the three melanoma patients (patient1, patient2, and patient3). Aberrant splicing‐derived neoantigens were from 4% to 15% (median was 9%). Only patient12 was detected to have one fusion‐derived neoantigen in the top 100 candidates. The alteration type of each candidate neoantigen of the top 100 in each patient is shown in Supplementary Figure S2. Detailed results are given in Supplementary Table S4.

### 2.4 Indirect immunogenicity evaluation strategy performs better than the direct strategy

TCR recognition on pMHC complexes is a key process of neoantigen‐induced immune responses. Peptides with strong potential to be recognized by T cells have a higher possibility of being true neoantigens. We prioritized candidate neoantigens by predicting the possibility of TCR recognition and set high ranks to the neoantigens with high possibilities. However, predicting TCR recognition is a challenging task due to the natural weak interaction between TCRs and pMHCs [22, 23]. We designed two strategies, indirect immunogenicity evaluation strategy and direct immunogenicity evaluation strategy, to estimate immunogenicity and perform neoantigen prioritization.

We adopted two recognition‐associated features, agretopicity [24] and foreignness [25], to indirectly estimate TCR recognition. Agretopicity measures the variation between the binding affinity of HLA alleles on mutant and wild‐type peptides. We hypothesized that a mutant peptide with significantly higher binding affinity than its wild‐type peptide, that is, a peptide with low agretopicity, has a high possibility of being immunogenic. Foreignness measures the similarity between a peptide and the confirmed antigens. We collected antigens from the Immune Epitope Database (IEDB) [26]. These antigens have been validated to interact with TCRs. We hypothesized that a mutant peptide that is highly similar to these antigens, that is, a peptide with large foreignness, has a high possibility of being immunogenic. We ranked candidate neoantigens with small agretopicity or large foreignness at the forefront (Group 1) and ranked the other candidates at the end (Group 2). We further ranked the candidates within each group based on binding affinity. In this way, candidate neoantigens with small agretopicity or large foreignness as well as strong binding affinity have superior priority. This strategy is the default prioritization approach of NeoHunter, and the experiments without specific declaration in this study were based on this strategy. Methods of estimating the two recognition‐associated features and the relevant prioritization strategy are described in Materials and Methods.

We directly evaluated the immunogenicity of TCR recognition on candidate neoantigens by decoding TCR clonotypes and predicting TCR–pMHCs using deep learning models. The key element in TCRs that is responsible for recognizing antigens is called complementarity‐determining region 3 (CDR3). We decoded CDR3 clonotypes of patients using MiXCR [27] from RNA‐seq data. We predicted the possibility of being in interaction between each candidate neoantigen and each CDR3 clone using ERGO [19], which is a recently developed deep‐learning‐based model that was trained with TCR–pMHC pairs and can predict the TCR specificity score that measures the possibility of the given peptide interacting with the given TCR. We predicted the TCR specificity scores on candidate neoantigens and prioritized these candidates based on these scores. Methods of decoding CDR3 clonotypes, predicting TCR specificity scores, and the relevant prioritization strategy are described in Materials and Methods.

**FIGURE 5:**
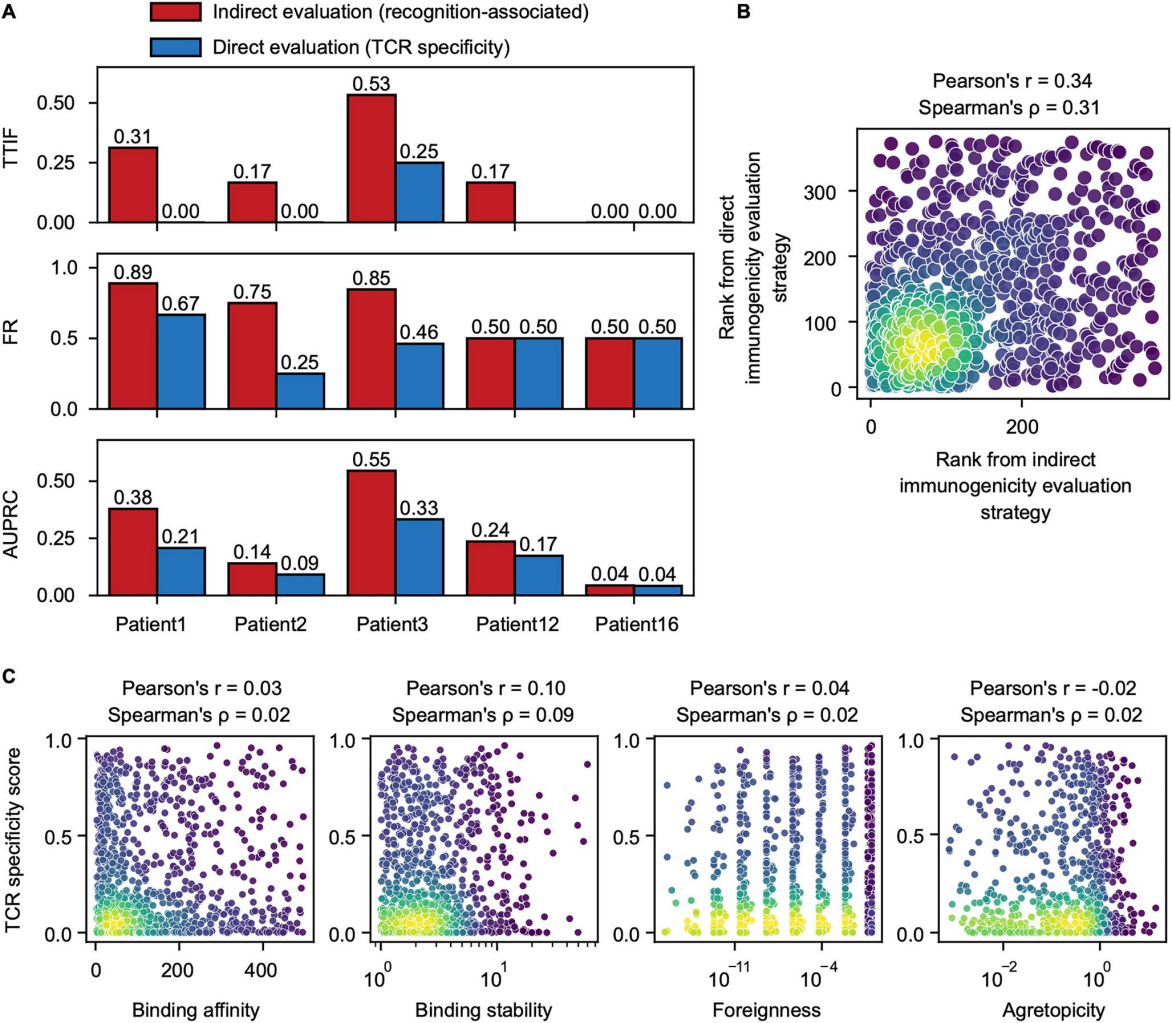
Comparison between the indirect and direct immunogenicity evaluation and prioritization strategy. (A) Performance of the indirect and direct immunogenicity evaluation and prioritization strategies on each patient. From the top panel to bottom panel, the results are for TTIF, fraction ranked (FR), and AUPRC. (B) Scatter plots showing the correlation between ranks from the indirect immunogenicity evaluation strategy (*x*‐axis) and ranks from the direct immunogenicity evaluation strategy (*y*‐axis). Each dot represents a candidate neoantigen from one patient. Light colors indicate high density. (C) Scatter plots showing the correlation between the T‐cell receptor (TCR) specificity score (*y*‐axis) and binding affinity (*x*‐axis, the leftmost panel), binding stability (*x*‐axis, the second panel from the left), foreignness (*x*‐axis, the third panel from the left), and agretopicity (*x*‐axis, the rightmost panel). Each dot represents a candidate neoantigen from one patient. Light colors indicate high density.

We found that the indirect immunogenicity evaluation strategy had consistently better performance than the direct strategy (Figure 5A). The indirect strategy obtained a significantly higher TTIF than the direct strategy. The indirect strategy obtained 0 to 0.53 TTIF across the patients, while only one patient obtained a nonzero TTIF under the direct strategy. This indicated that there were nearly no immunogenic peptides in the top 20 candidates using the direct strategy, while the indirect strategy ranked some immunogenic peptides with high prioritization. The indirect strategy also obtained some improvement over the direct strategy on the three melanoma patients on the FR metric, indicating that these three patients detected more immunogenic peptides in the top 100 candidates. For the AUPRC, the indirect strategy also showed better or comparable performance in all patients, suggesting that the immunogenic peptides identified by the indirect strategy had higher priority. Ranks from the two strategies showed moderate consistency. The Pearson’s correlation and Spearman’s correlation coefficient of these two ranks were 0.34 and 0.31, respectively (Figure 5B). In addition, Pearson’s correlation and Spearman’s correlation coefficients between the TCR specificity score derived from the direct strategy and the recognition‐associated features involved in the indirect strategy, such as agretopicity, foreignness, and binding affinity, were approximately 0 (Figure 5C). The TCR specificity score and features involved in indirect strategies, such as agretopicity, foreignness, and binding affinity may be independent. The results suggested that the features of the direct and indirect strategies may depict the characteristics of the peptide from different sides. The results of each patient can be found in Supplementary Figures S3–S6. We also discovered correlations among binding affinity, binding stability, foreignness, and agretopicity. Only binding affinity and binding stability had a moderate correlation (Pearson’s correlation and Spearman’s correlation coefficients among these patients were −0.244 and −0.435, respectively). Correlations between the other features were weak (Supplementary Figures S7–S11). Although the performance of the direct immunogenicity evaluation strategy does not work as well as the indirect strategy currently, it may yield improvement gains in the future with the development of technology for decoding TCR types and structures.

### 2.5 Detecting neoantigens derived from different alterations requires different sequencing depths

We inspected the impacts of sequencing depth on detecting neoantigens from different alterations. We randomly downsampled the DNA and RNA sequencing data of patient1 at different depths, and each depth was repeated five times (see Materials and Methods for details). We detected neoantigens derived from the four alterations using these downsampled data.

**FIGURE 6:**
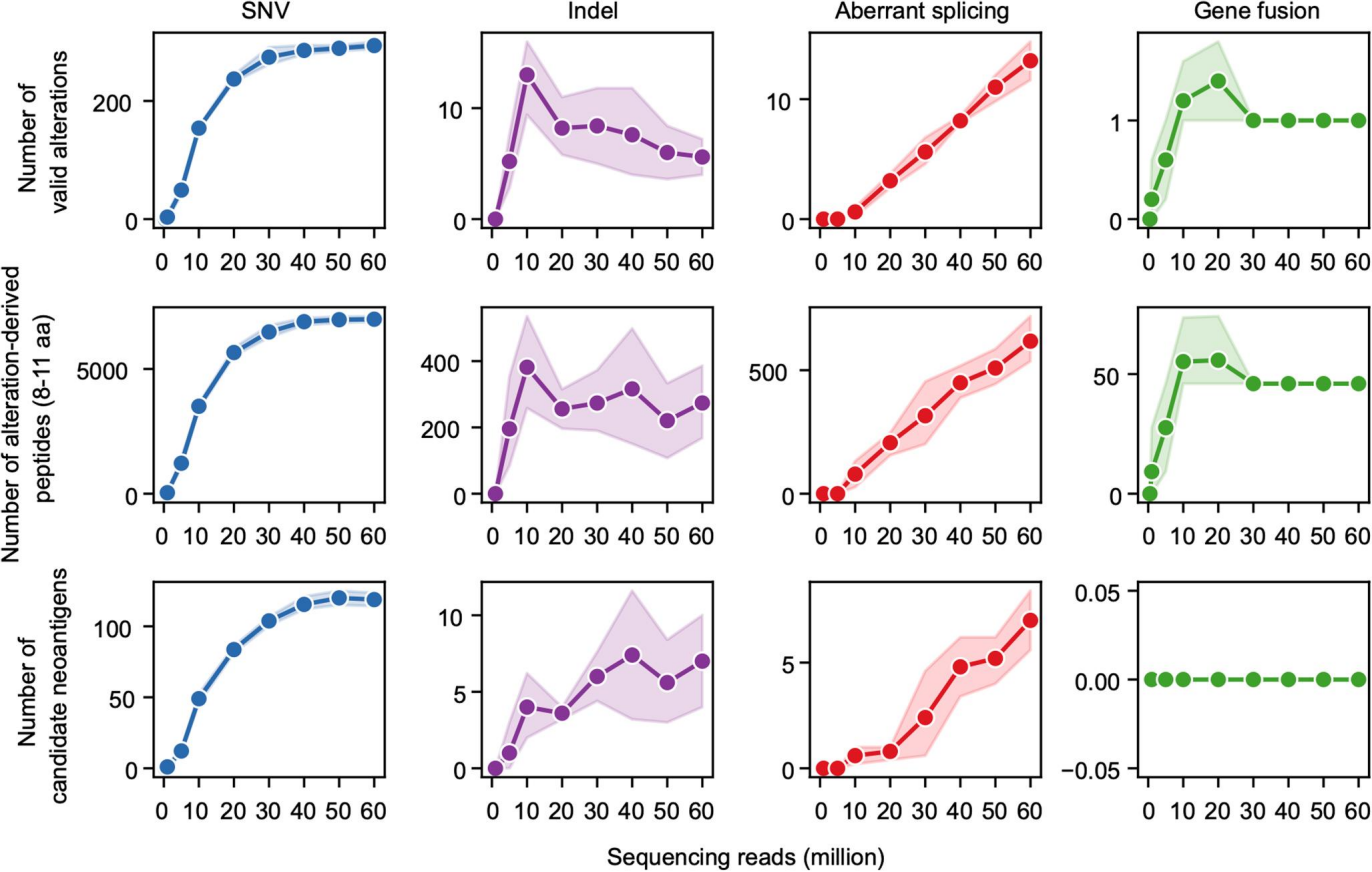
Evaluation of the impact of sequencing depth on detecting different types of alteration and alteration‐derived neoantigens. Each column represents one type of alteration. From top to bottom, each row represents the relationship between the depth of sequencing reads and the number of valid alterations, the number of alteration‐derived peptides, and the number of alteration‐derived candidate neoantigens. The results are measured as averaged results across the five repeats. Error bands are confidence intervals (95%) determined across five repeats. aa, amino acids.

We found that detecting neoantigens from different types of alterations required different suitable sequencing depths (Figure 6). SNV‐ and indel‐derived neoantigens were detected from DNA sequencing data. The number of detected valid SNVs tended to stabilize when the number of sequencing reads was larger than 40 million. The number of SNV‐derived peptides and SNV‐derived candidate neoantigens showed similar results. The number of detected valid indels and the number of peptides derived from them were slightly unstable as the number of sequencing reads increased. The number of indel‐derived neoantigens fluctuated by approximately 5, with sequencing reads ranging from 10 million to 60 million.

Aberrant splicing‐ and gene fusion‐derived neoantigens were detected from RNA‐seq data. We found that detecting aberrant splicing requires enough sequencing reads. The number of detected valid aberrant splicing events and their downstream peptides and the number of aberrant splicing‐derived candidate neoantigens increased continually as the number of sequencing reads increased to 60 million. This indicated that more aberrant splicing‐derived neoantigens may be detected if deeper sequenced RNA‐seq data of a patient were obtained. Detected valid gene fusions became stable when sequencing reads increased to 30 million. However, the number of gene fusion‐derived candidate neoantigens remained at 0. This may be due to the nature of the cancer type and the cancer status of the patient.

### 2.6 NeoHunter supports multiple SNV and indel annotation models

We adopted Variant Effect Predictor (VEP) [28] to annotate the function of SNVs and indels by default. In addition, NeoHunter also supported the other two annotation models, SnpEff [29] and the Funcotator module within GATK [30] (see Materials and Methods for details). We compared the performance of these three annotation models. VEP and SnpEff tended to output more valid SNVs/indels; thus, the number of candidate neoantigens was also greater than that from Funcotator. VEP and SnpEff obtained similar performance on TTIF, FR, and the detection rate, while TTIF and FR of Funcotator on most patients were worse. This indicated that more immunogenic peptides could be properly annotated by VEP or SnpEff. The results can be found in Supplementary Figure S12.

## 3 DISCUSSION

In this study, we developed NeoHunter to systemically detect neoantigens derived from multiple sources. Compared with previous neoantigen detection algorithms that mainly focused on neoantigens derived from SNVs and indels, NeoHunter paid additional attention to neoantigens derived by other types of molecular alterations such as gene fusion and aberrant splicing. Validation results on TESLA cancer cohorts showed that aberrant splicing events occupied a significant proportion (from 4% to 15%) of the top 100 candidate neoantigens, indicating that aberrant splicing‐derived neoantigens should be fully considered during cancer vaccine design. We also detected one gene fusion‐derived neoantigen from a NSCLC patient (patient12). Many fusion events have been discovered to drive cancer development in lung cancers, and some relevant targeted drugs, such as Crizotinib and Lorlatinib, have shown potential for treatment [31]. All the results suggested that NeoHunter is essential to comprehensive neoantigen detection.

NeoHunter achieved superior performance in detecting SNV‐ and indel‐derived neoantigens compared to existing methods. It could indirectly estimate TCR recognition on candidate neoantigens by estimating recognition‐associated features such as agretopicity and foreignness. It could also directly estimate the TCR–pMHC interaction by deep learning models trained by known TCR–pMHC data. We detected 27 out of 34 validated immunogenic peptides in total from the TESLA patients under the indirect strategy. In the top 100 candidate neoantigens, the number of validated immunogenic neoantigens detected from NeoHunter was almost always more than the other 25 neoantigen detection methods. NeoHunter could even discover all validated immunogenic neoantigens from a melanoma patient (patient2), while the other methods could at best discover 75% of them. We found that the indirect strategy showed better performance than the current direct strategy. However, we believe that, with the accumulation of TCR–pMHC data and the development of TCR decoding technologies, the direct strategy may gain significant enhancements and play a more important role in the future.

It should be noted that current neoantigen detection is based on bulk sequencing data. However, tumors and immune systems are highly heterogeneous. Molecular alterations may occur in different parts of tumors. Diverse molecular alterations form distinct subclones and drive cancer evolution and metastasis. The downstream products in different subclones have different biological effects [32, 33, 34], promoting drug resistance to targeted anticancer agents [32, 35]. Similarly, different subclones may also have distinct sequences in the coding regions of HLA genes [36]. These variations located on HLAs may change the presentation function of HLAs. In addition, TCR recognition of pMHC mainly relies on the hypervariable CDR3 regions in TCR genes. The CDR3 regions are composed of a TCR alpha chain and beta chain, encoded by TRA genes and TRB genes, respectively [37]. We can only estimate the clonotypes of TRA and TRB independently via bulk sequencing data, and joint pairing TRA and TRB is not yet possible. For all these reasons, bulk sequencing data cannot completely reflect the entire tumor immune landscape and constrain the effective discovery of neoantigens. Full‐length single‐cell sequencing technologies are rapidly developing. Some recent studies have shown the possibility of detecting alterations [33], typing HLAs [38], and estimating TCRs [39, 40] at single‐cell resolution. Thus, we plan to extend the current NeoHunter to single‐cell cancer data in the future. We believe that a well‐designed neoantigen detection method based on single‐cell data will help to overcome the problems above, improve neoantigen detection performance, and provide better guidance for precision cancer treatment.

## 4 CONCLUSION

To the best of our knowledge, NeoHunter is the first software to simultaneously detect and prioritize neoantigens derived from all common molecular alterations, including SNVs, indels, gene fusions, and aberrant splicing. Versatile neoantigen prioritization strategies ensure that the findings are more robust and reliable. We believe that NeoHunter will help to advance research on personalized cancer vaccine development, and NeoHunter is available for free academic use on Github (XuegongLab/NeoHunter).

## 5 MATERIALS AND METHODS

### 5.1 SNV/indel detection

We detected SNVs and indels from the WGS/WES data of cancer patients. We first mapped both normal and tumor WES data using BWA (version 0.7.17) [41] against the reference genome hg19. Then we applied SAMtools (version 1.9) [42] to process the index and sort the aligned sequences. We then fed the aligned sequences into the short variant discovery model Genome Analysis Toolkit (GATK, version 4.2.6.1) [30]. We detected SNVs and indels using the Mutect2 module of GATK with default settings.

NeoHunter flexibly accepts multiple kinds of genomic data as inputs. In this study, all SNVs and indels were detected from the raw genome sequencing reads (fastq‐formatted). In addition, aligned sequences (bam‐formatted or sam‐formatted) and variant calls (vcf‐formatted) were also acceptable.

### 5.2 SNV/indel annotation

We annotated SNVs and indels using Ensembl Variant Effect Predictor (VEP, version 105) [28] by default and thus confirmed the downstream influences of variants. We only considered the variant that resulted in a change in the downstream amino acid sequence. For SNVs, we only considered the variants annotated as “missense_variant”. For indels, we only considered the variants annotated as “inframe_insertion”, “inframe_deletion”, or “frameshift_variant”. In addition, we only considered variants that satisfied the following conditions: (1) abundance of the transcript the SNV/indel located in was greater than 1 transcripts per million (TPM) (the method of transcript quantification was described in the following); (2) variant allele frequency of the tumor sample was greater than or equal to 0.1; (3) variant allele frequency of the normal sample was less than or equal to 0.05; and (4) the number of sequencing reads covering the variant loci in the tumor sample was greater than or equal to 5.

NeoHunter can flexibly parse the results of multiple variant annotation models. In addition to VEP, SnpEff (version 5.1) [29] and the Funcotator module within GATK [30] were also acceptable.

### 5.3 Gene fusion detection

We detected gene fusions from the RNA‐seq data through STAR‐Fusion (version 1.11.0) [43] with default settings. We only considered the fusion event that merged coding regions of parental genes, that is, the fusion events annotated as ‘INFRAME’ and ‘FRAMESHIFT’. STAR‐Fusion provided a metric called FFPM (fusion fragments per million total reads), which measured the supporting evidence on the detected fusion event. We only considered fusion events that satisfied the following conditions: (1) FFPM of the fusion event was greater than or equal to 0.1 (at least one fusion supporting fragment per million reads); (2) abundance of the fusion transcript was greater than 1 TPM (the method of transcript quantification is described in the following).

### 5.4 Aberrant splicing detection

We detected aberrant splicing from the RNA‐seq data based on ASNEO [44]. We first detected splice junctions using STAR (version 2.7.8) [45]. Only junctions with more than 10 unique mapped reads covered were retained. Then, the retaining junctions were compared to a set of normal junctions, and only junctions that did not belong to the set of normal junctions were considered abnormal. Normal junctions were collected from two sources. The junctions that were covered by at least 2 sequencing reads and occurred in at least 1% of normal samples from genotype‐tissue expression (GTEX) [46] were defined as normal junctions. In addition, the junctions that occur in the human reference genome were also considered as normal junctions. Then, the abnormal junctions were inserted into the reference isoforms. The generated abnormal peptides were further compared to normal peptides from the reference genome and GTEX to remove false discoveries. We only kept the isoform with an abundance greater than 1 TPM (the method of transcript quantification is described in the following).

### 5.5 Alteration‐derived peptide generation

We translated mutated nucleotide sequences into amino acid sequences using the codon table. We then split the amino acid sequences into peptides with a sliding window of 8, 9, 10, and 11 amino acids.

### 5.6 Transcript and peptide quantification

We quantified transcript abundance in TPM units from the RNA‐seq data using Kallisto (version 0.48.0) [47] with default settings. For SNV‐, indel‐, and aberrant splicing‐derived peptides, the expression level was quantified as the expression level of the transcripts where the SNVs, indels, and aberrant splicing variants were located. For fusion‐derived peptides, the expression level was quantified as the average of the two parental transcripts.

### 5.7 HLA typing

We typed HLA alleles in silico from the RNA‐seq data through Optitype (version 1.3.2) [48] with default settings. The HLA alleles were typed at four‐digit resolution.

### 5.8 Peptide–MHC binding prediction

We estimated the binding affinity between each peptide and each HLA allele using NetMHCpan (version 4.1) [49]. The output prediction binding score of the query peptide was compared to the prediction binding scores of a set of random natural peptides. Such a comparison could result in a percent rank of the prediction binding score of the query peptide among backgrounds. We only retained the peptide–MHC of which the percent rank was less than 2%. In addition, we retained pMHCs with binding ability less than 500 nM.

We then estimated the binding stability between each peptide and MHC using NetMHCstabpan (version 1.0) [50]. We only retained pMHCs of which the binding stability was greater than 1 h. The remaining peptides were considered candidate neoantigens.

### 5.9 Immunogenicity evaluation and prioritization strategy

We prioritized the candidate neoantigens with the possibility of the candidate peptide being recognized by TCRs. NeoHunter supported two strategies to evaluate this immunogenicity. NeoHunter could indirectly estimate the immunogenicity evaluation with the recognition‐associated features which reflected the similarity between the candidate peptide and confirmed antigens and the dissimilarity between the candidate peptide and its wild‐type. In addition, NeoHunter could also directly estimate the TCR–pMHC interaction using deep‐learning‐based models. Candidate neoantigens could be ranked using relevant evaluation scores. Detailed methods are described below.

### 5.10 Recognition‐associated feature estimation

Recognition‐associated features indicated two metrics, agretopicity [24] and foreignness [25]. The two metrics were found to be highly informative for discovering immunogenic peptides [21]. Specifically, agretopicity represents the ratio of the binding affinity of wild‐type peptides to mutant peptides and is defined in Equation (1), where $K_{d}^{WT}$ represents the MHC binding affinity on wild‐type peptides and $K_{d}^{MT}$ represents the MHC binding affinity on mutant peptides. Less agretopicity indicates that the mutant peptide has a stronger potential to be immunogenic. Foreignness represents the similarity between the query peptide and the known antigens in the Immune Epitope Database (IEDB) [26]. Foreignness is defined in Equation (2), where *D* represents the set of antigens collected from the IEDB, *s* represents the query peptide, |e,s| represents sequence alignment based on the BLOSUM62 similarity matrix [51], and coefficients α=26 and *k* = 4.87 are maintained from the original research [25]. A greater foreignness indicates that the query peptide has a higher possibility of being immunogenic.

(1) $$A=\frac{K_{d}^{MT}}{K_{d}^{WT}}$$

(2) $$F=\frac{\sum_{e\in D}\exp\left(-k\left(a-\left|e,s\right|\right)\right)}{1+\sum_{e\in D}\exp\left(-k\left(a-\left|e,s\right|\right)\right)}$$

### 5.11 Indirect immunogenicity evaluation and prioritization strategy

We prioritized candidate neoantigens using the indirect evaluation strategy by the following steps: (1) dividing all neoantigens into two groups. Group 1 included the peptides with agretopicity less than 0.1 or peptides with foreignness greater than $10^{-12}$. We considered the peptides in group 1 having strong potential to be immunogenic. Peptides that did not satisfy the conditions above were classed into group 2 and were considered to be less immunogenic; (2) ranking the pMHC binding affinity (stronger binding affinity indicating higher priority) within group 1 and group 2 separately; (3) appending ranked peptides of group 2 to ranked peptides of group 1.

### 5.12 TCR repertoire analysis

We identified clonotypes of TCRs from the RNA‐seq data using MiXCR (version 4.0.0) [27]. MiXCR aligned raw sequencing reads against reference TCR genetic segments. Then it automatically corrected artificial errors and clustered similar nucleotide sequences to assemble clonotypes. Then, clonotypes that were out‐of‐frame or stop‐codons were filtered out. Only CDR3 peptide sequences from TCR alpha and TCR beta chains were considered.

### 5.13 TCR–pMHC binding specificity prediction

We estimated TCR specificity using ERGO (version 2, in “AE” mode, i.e., autoencoder‐based mode) [19]. ERGO was trained with tens of thousands of TCR–peptide pairs and could output a confidence score of TCR specificity (i.e., TCR specificity score) given the CDR3 and antigen amino acid sequence.

### 5.14 Direct immunogenicity evaluation and prioritization strategy

We prioritized candidate neoantigens by ranking the TCR specificity score (stronger TCR binding confidence indicating higher priority).

### 5.15 Evaluation metric

We adopted four metrics to evaluate the algorithm performance. The set of immunogenic peptides validated by the TESLA was denoted as *V*. The set of candidate peptides tested by the TESLA was denoted as *T*. The top *n* ranked candidate neoantigens predicted by neoantigen detection algorithms were denoted as $C^{\left(n\right)}$. All candidate neoantigens predicted by neoantigen detection algorithms were denoted as *C*. TTIF, top 20 immunogenic fraction, is the fraction of validated immunogenic peptides in the tested peptides from the top 20 candidates. Its formula is

$$\text{TTIF}=\frac{\left|C^{\left(20\right)}\cap V\right|}{\left|C^{\left(20\right)}\cap T\right|}$$

FR, fraction ranked, measures the fraction of validated immunogenic peptides of the tested peptides from the top 100 candidates against all immunogenic peptides from the TESLA. Its formula is

$$\text{FR}=\frac{\left|C^{\left(100\right)}\cap V\right|}{\left|V\right|}$$

AUPRC, the area under the precision recall curve, reflects the discriminant ability on immunogenic and non‐immunogenic peptides. The union of these two kinds of peptides is C∩T.

Detection rate represents the proportion of immunogenic peptides of candidates against all immunogenic peptides from the TESLA. Its formula is

$$\text{Detection}\;\text{rate}=\frac{\left|C\cap V\right|}{\left|V\right|}$$

### 5.16 Sequencing read downsampling

We randomly downsampled sequencing reads at different depths from the sequencing data of patient1 to study the impact of sequencing depth. The downsampling depths on DNA sequencing data of the normal and tumor samples and RNA sequencing data of the tumor sample were 0.1, 0.5, 1, 5, 10, 20, 30, 40, 50, and 60 million reads. We repeated the downsampling on each depth five times.

## AUTHOR CONTRIBUTIONS

**Tianxing Ma**: Data curation; formal analysis; methodology; software; visualization; writing – original draft preparation. **Zetong Zhao**: Data curation; formal analysis; software. **Haochen Li**: Formal analysis. **Lei Wei**: Funding acquisition; writing – review and editing. **Xuegong Zhang**: Conceptualization; funding acquisition; resources; supervision; writing – review and editing.

## CONFLICT OF INTEREST STATEMENT

The authors Tianxing Ma, Zetong Zhao, Haochen Li, Lei Wei and Xuegong Zhang declare that they have no conflicts of interest. This method is being applied for a patent.

## ETHICS STATEMENT

This article does not contain any studies with human or animal subjects performed by any of the authors.

## Supporting information

Supporting Information S1

## Data Availability

The supplementary materials can be found online with this article.
