# Supplementary material for: NeoHunter: Flexible software for systematically detecting neoantigens from sequencing data
Source: Quant Biol. 2024 Jan 22;12(1):70–84. doi: 10.1002/qub2.28 (PMC12806199; doi:10.1002/qub2.28)
Supplement: Supplementary file 1 — Supporting Information S1 [file QUB2-12-70-s001.pdf]

Supplementary Materials

NeoHunter: flexible software for systematically detecting neoantigens from sequencing data

Tianxing Ma[1,†], Zetong Zhao[1,†], Haochen Li[2], Lei Wei[1], and Xuegong Zhang[1,2,3*]

[1]*MOE Key Lab of Bioinformatics, Bioinformatics Division of BNRIST and Department of Automation, Tsinghua University, Beijing 100084, China*
[2]*School of Medicine, Tsinghua University, Beijing 100084, China*
[3]*School of Life Sciences, Tsinghua University, Beijing 100084, China*
[†]*These authors contributed equally to this work*
[*]*Correspondence: zhangxg@tsinghua.edu.cn*

Supplementary Figures

Figure S1 Types of alterations generating the top 100 candidate neoantigens 2
Figure S2 Overlap between candidates of TESLA and NeoHunter 3
Figure S3 Correlation between the TCR specificity score and binding affinity 4
Figure S4 Correlation between the TCR specificity score and binding stability 5
Figure S5 Correlation between the TCR specificity score and foreignness 6
Figure S6 Correlation between the TCR specificity score and agretopicity 7
Figure S7 Pairwise correlations between features of patient1 8
Figure S8 Pairwise correlations between features of patient2 9
Figure S9 Pairwise correlations between features of patient3 10
Figure S10 Pairwise correlations between features of patient12 11
Figure S11 Pairwise correlations between features of patient16 12
Figure S12 Comparison between performance of multiple annotation models 13

Supplementary Tables

Table S1 Comparison of neoantigen detection softwares 14
Table S2 Number of valid alterations and alteration-derived peptides 16
Table S3 Ranked candidate neoantigens of the TESLA patients 17
Table S4 Descriptions of the TESLA data 34

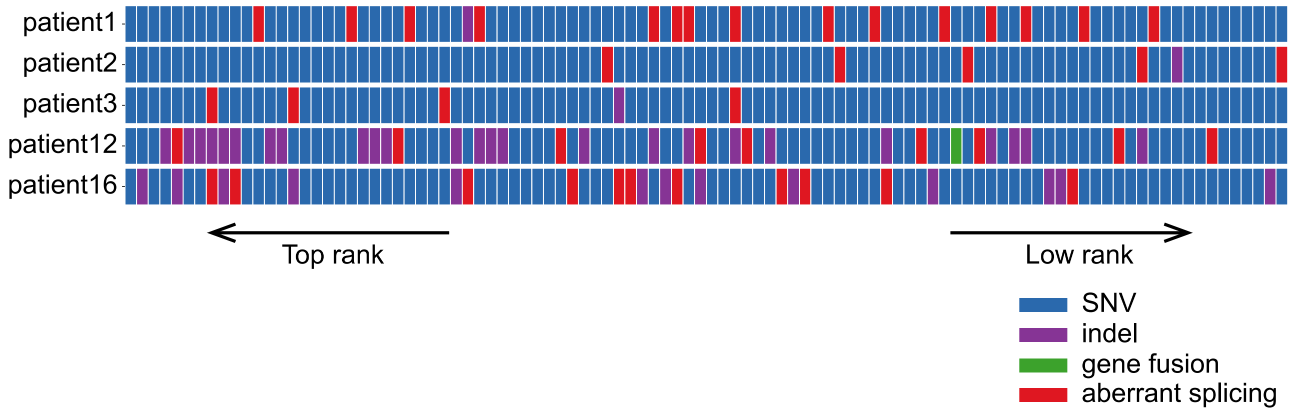

Figure S1 : Types of alterations generating the top 100 candidate neoantigens. Each row represents a patient. From left to right, each column represents a candidate neoantigens from high rank to low rank. The color of the bar indicates type of the alteration.

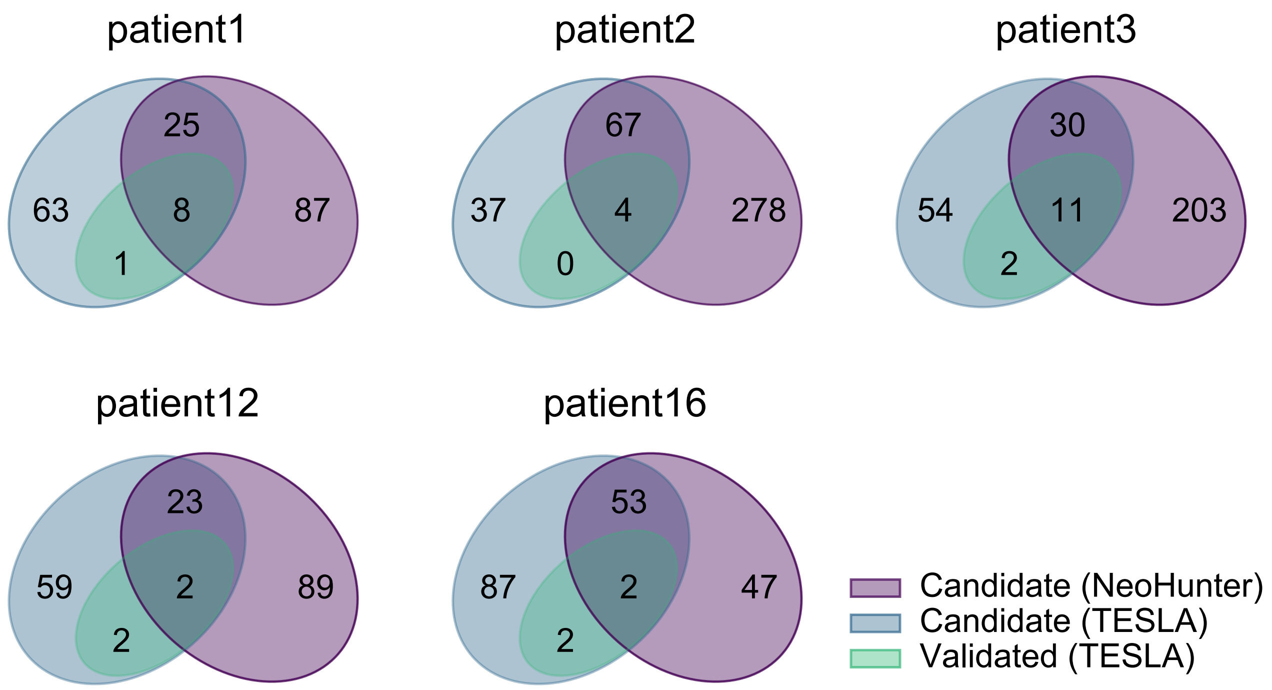

Figure S2 : Overlap between candidates of TESLA and NeoHunter. Venn diagrams summarizing the overlap between candidate peptides tested/reported by TESLA and NeoHunter and the overlap between immunogenic peptides validated/reported by TESLA and NeoHunter.

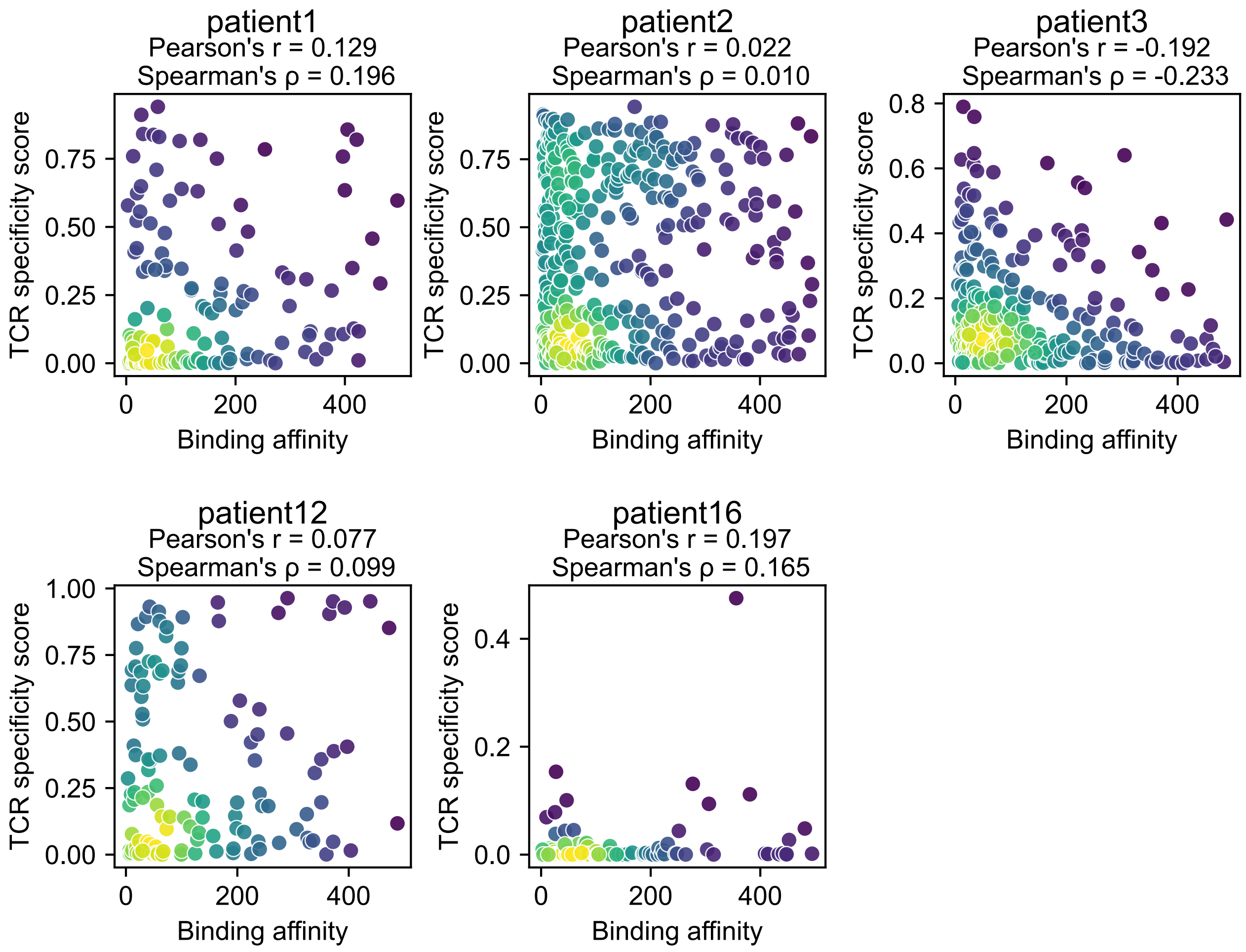

Figure S3 : Correlation between the TCR specificity score and binding affinity. Scatter plots showing the correlation between the TCR specificity score (y-axis) and binding affinity (x-axis). Each dot represents a candidate neoantigen. Light colors indicate high density.

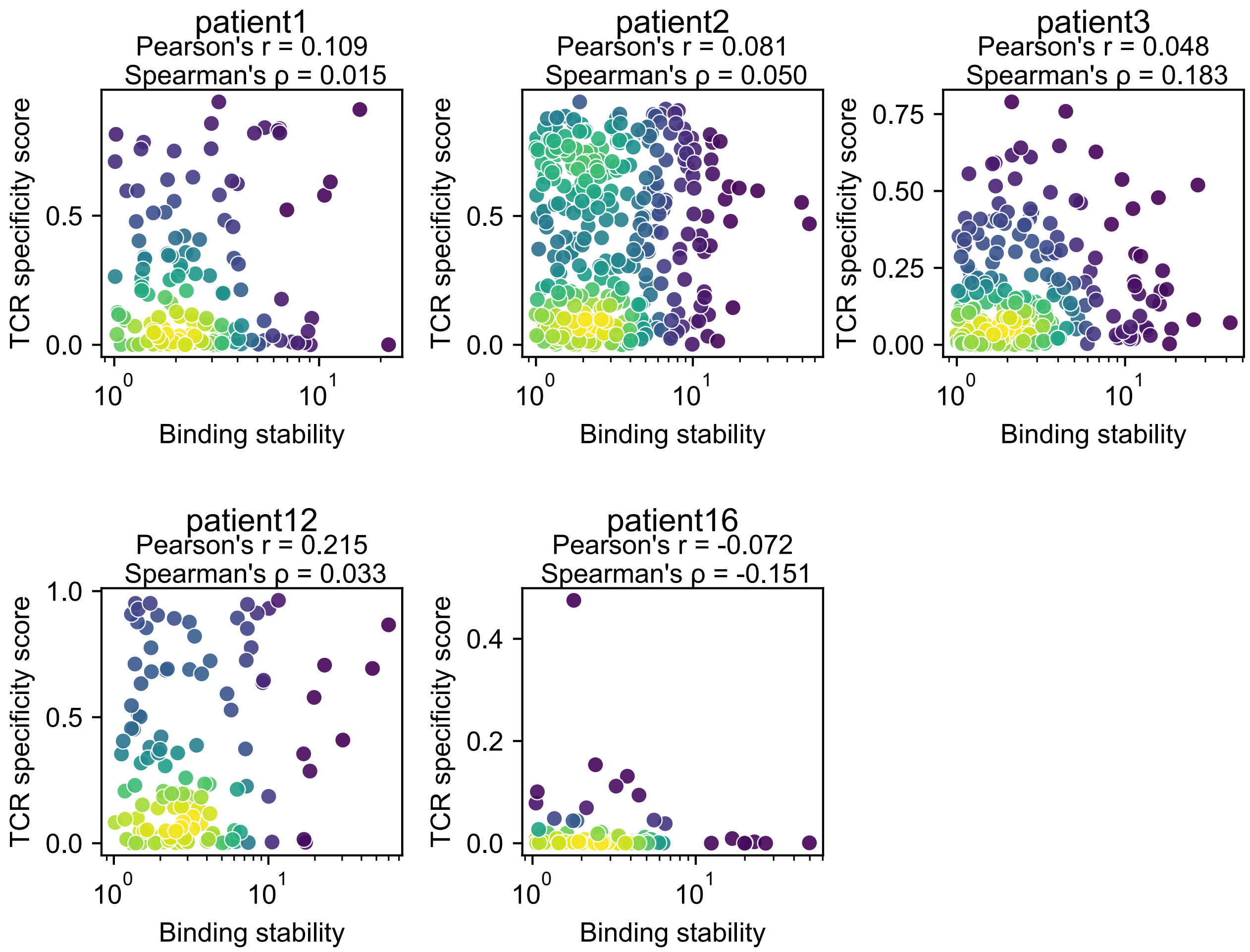

Figure S4 : Correlation between the TCR specificity score and binding stability. Scatter plots showing the correlation between the TCR specificity score (y-axis) and binding stability (x-axis). Each dot represents a candidate neoantigen. Light colors indicate high density.

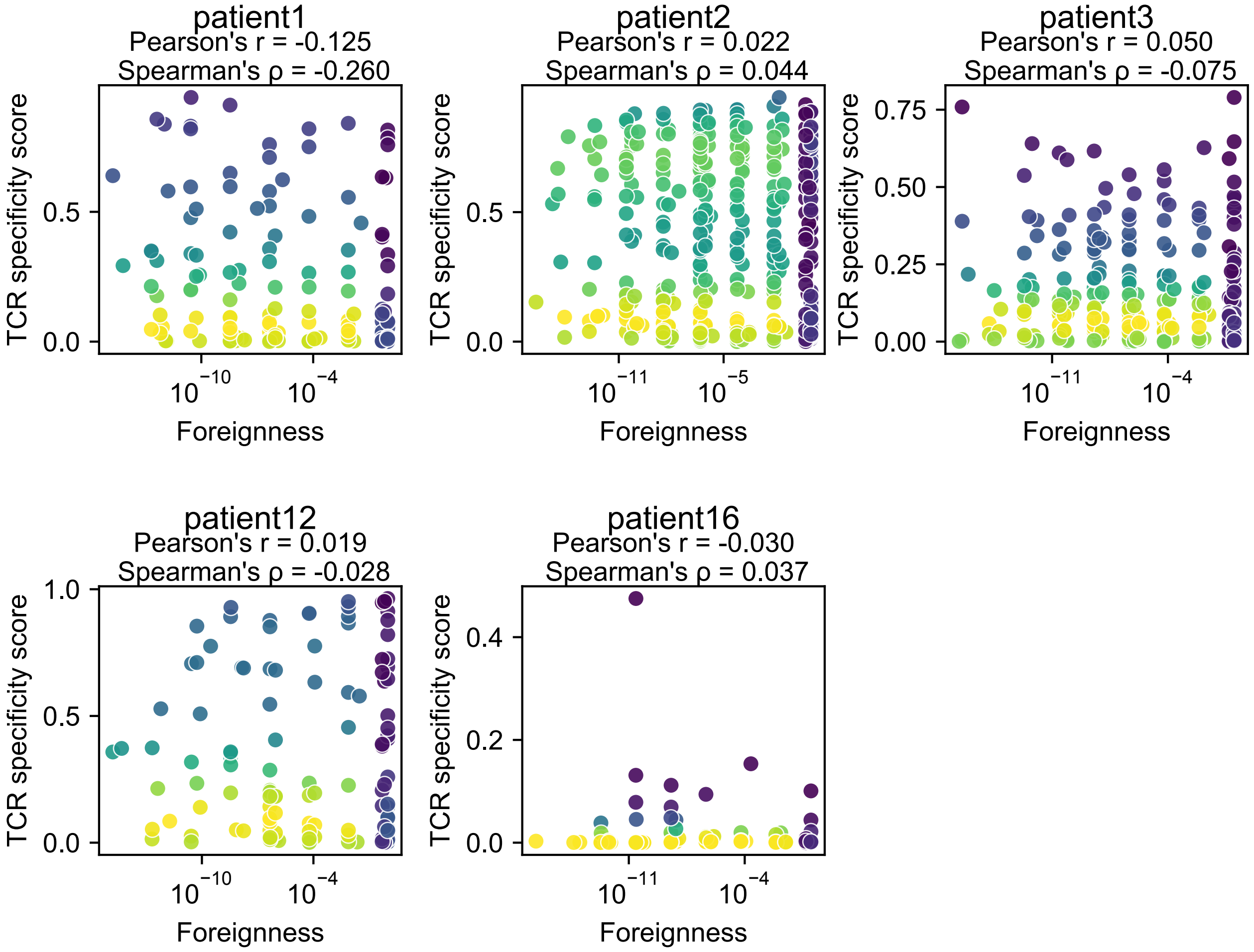

Figure S5 : Correlation between the TCR specificity score and foreignness. Scatter plots showing the correlation between the TCR specificity score (y-axis) and foreignness (x-axis). Each dot represents a candidate neoantigen. Light colors indicate high density.

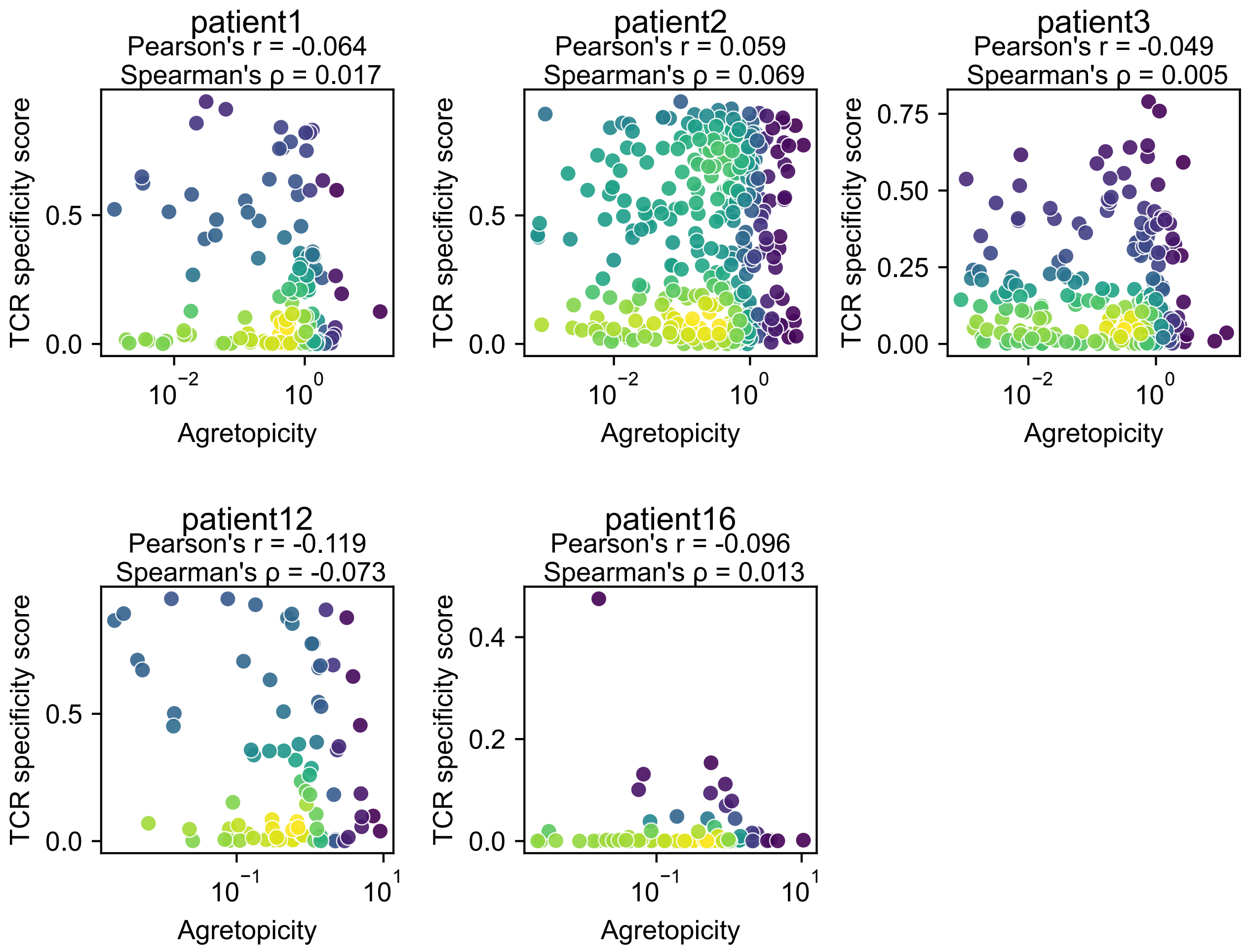

Figure S6 : Correlation between the TCR specificity score and agretopicity. Scatter plots showing the correlation between the TCR specificity score (y-axis) and agretopicity (x-axis). Each dot represents a candidate neoantigen. Light colors indicate high density.

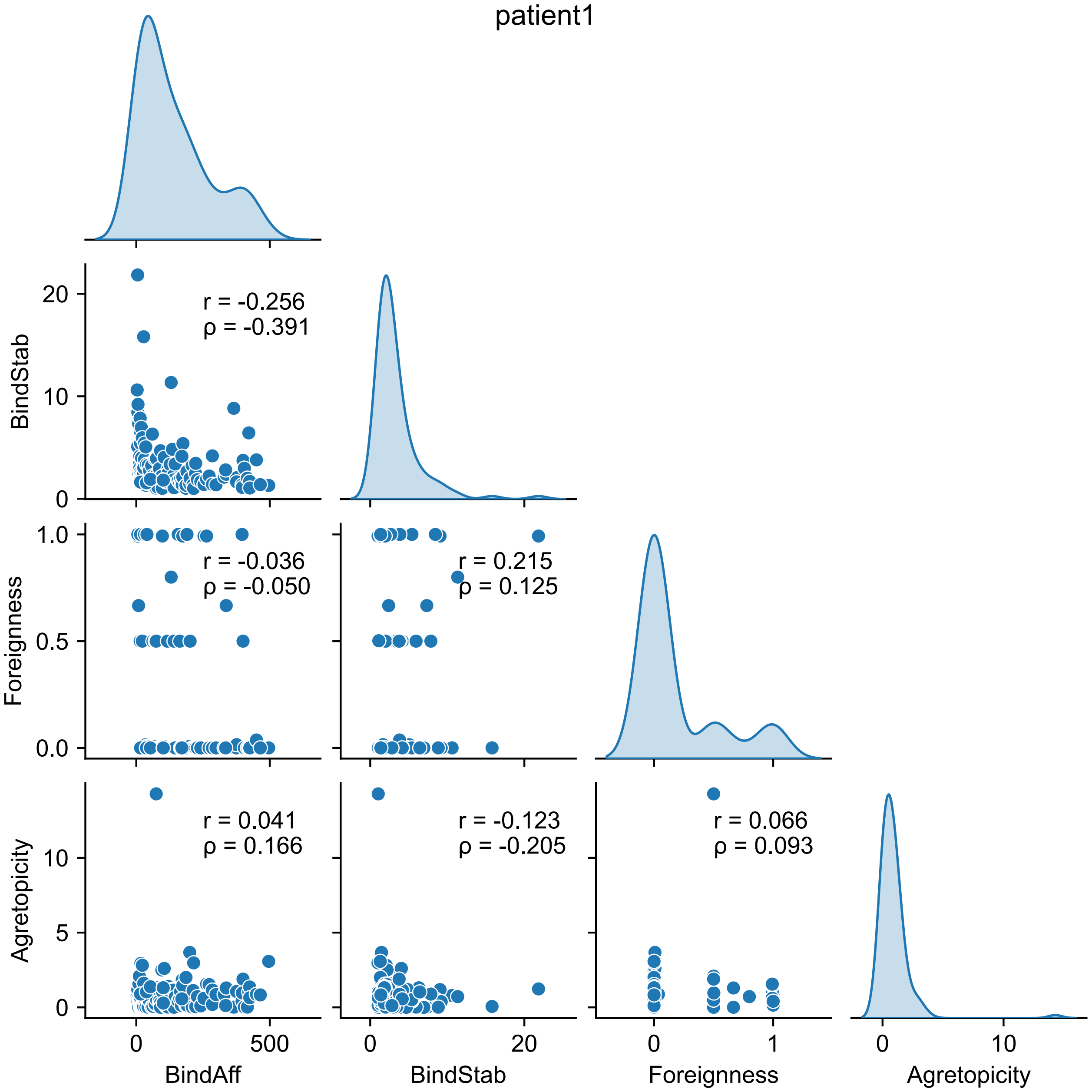

Figure S7 : Pairwise correlations between features of patient1. Pairwise correlations among binding affinity, binding stability, foreignness, and agretopicity of patient1. Each dot represents a candidate neoantigen. r represents Pearson's correlation coefficient. ρ represents Spearman's correlation coefficient.

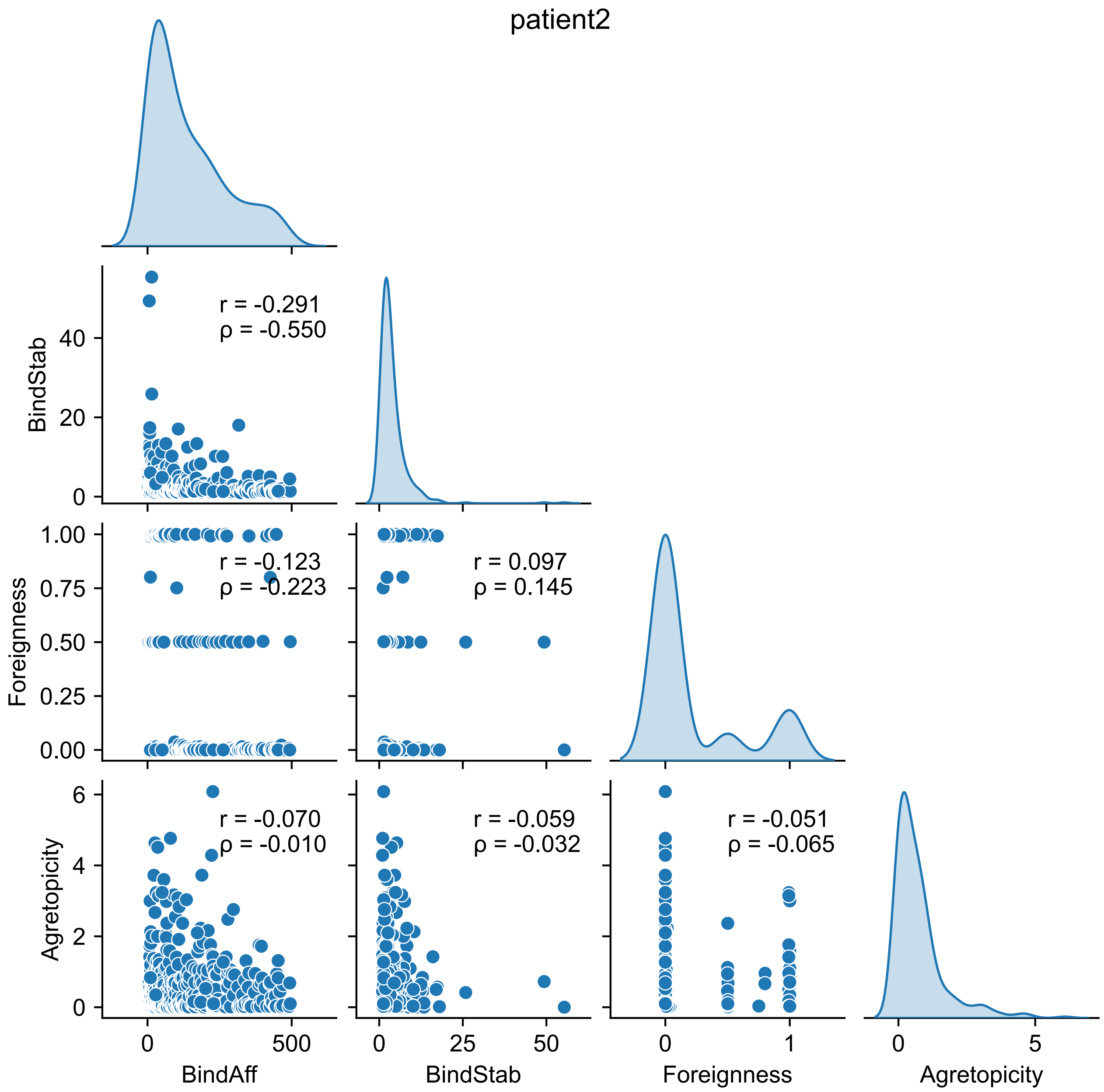

Figure S8 : Pairwise correlations between features of patient2. Pairwise correlations among binding affinity, binding stability, foreignness, and agretopicity of patient2. Each dot represents a candidate neoantigen. r represents Pearson's correlation coefficient. ρ represents Spearman's correlation coefficient.

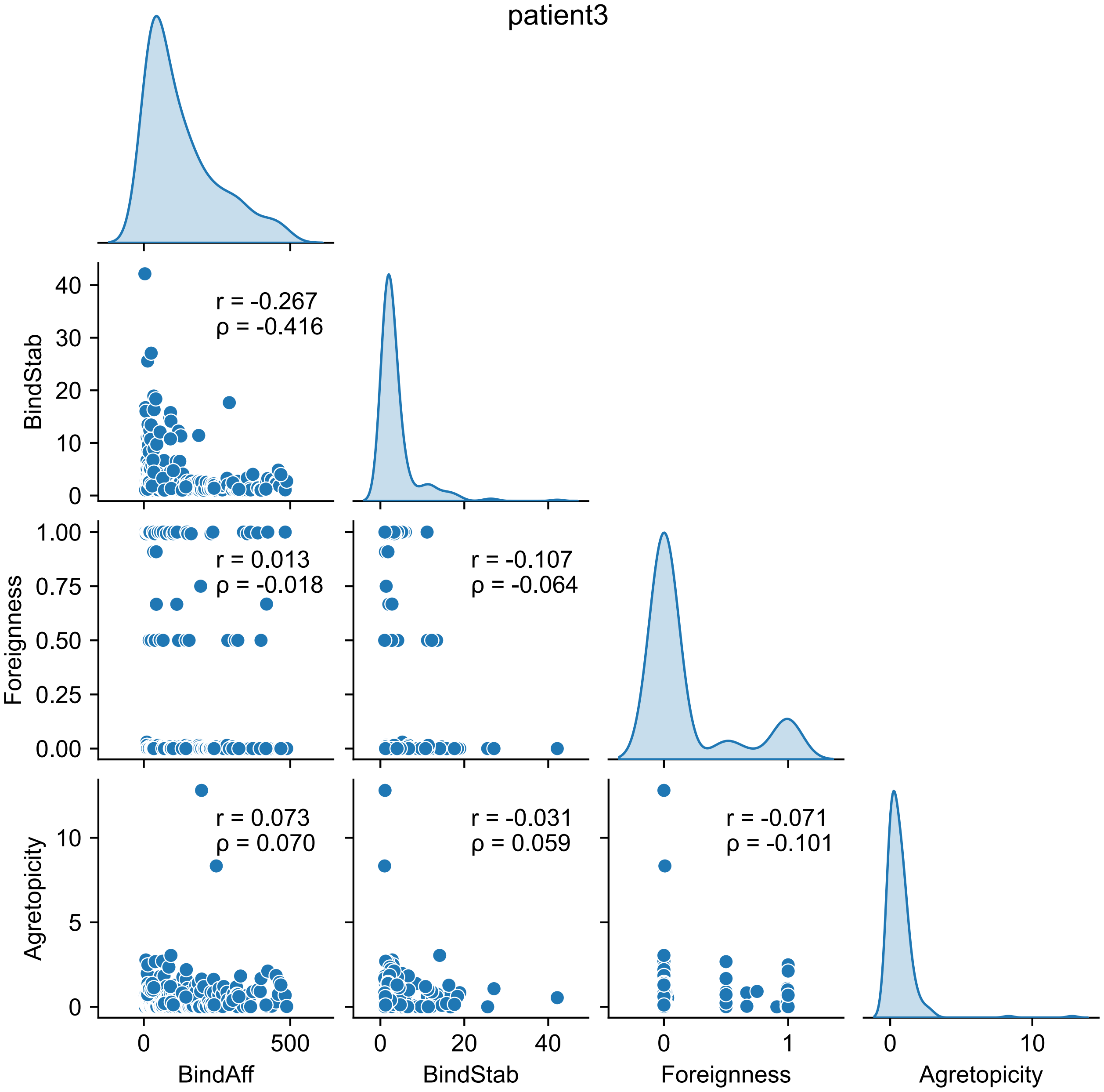

Figure S9 : Pairwise correlations between features of patient3. Pairwise correlations among binding affinity, binding stability, foreignness, and agretopicity of patient3. Each dot represents a candidate neoantigen. r represents Pearson's correlation coefficient. ρ represents Spearman's correlation coefficient.

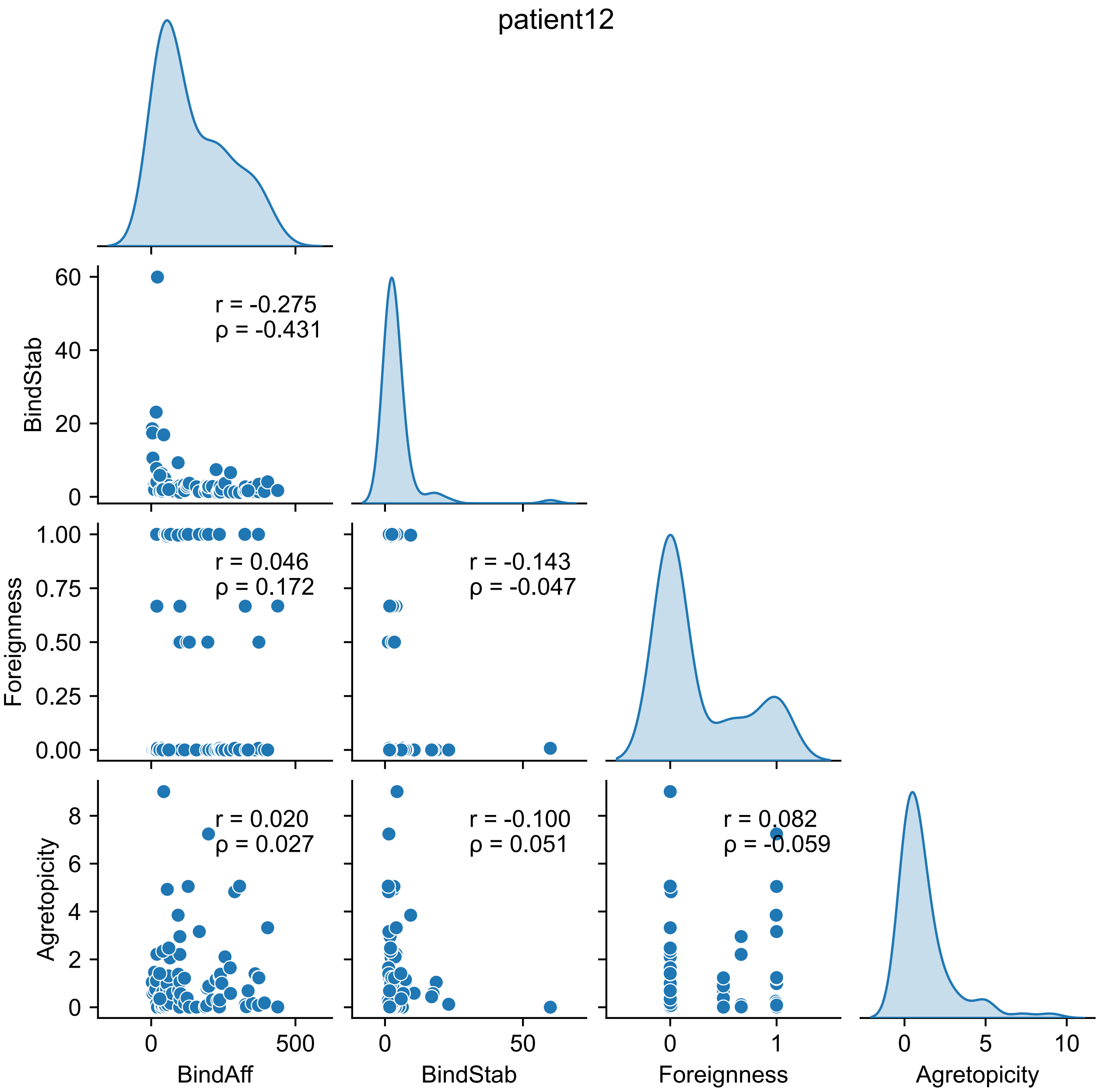

Figure S10 : Pairwise correlations between features of patient12. Pairwise correlations among binding affinity, binding stability, foreignness, and agretopicity of patient12. Each dot represents a candidate neoantigen. r represents Pearson's correlation coefficient. ρ represents Spearman's correlation coefficient.

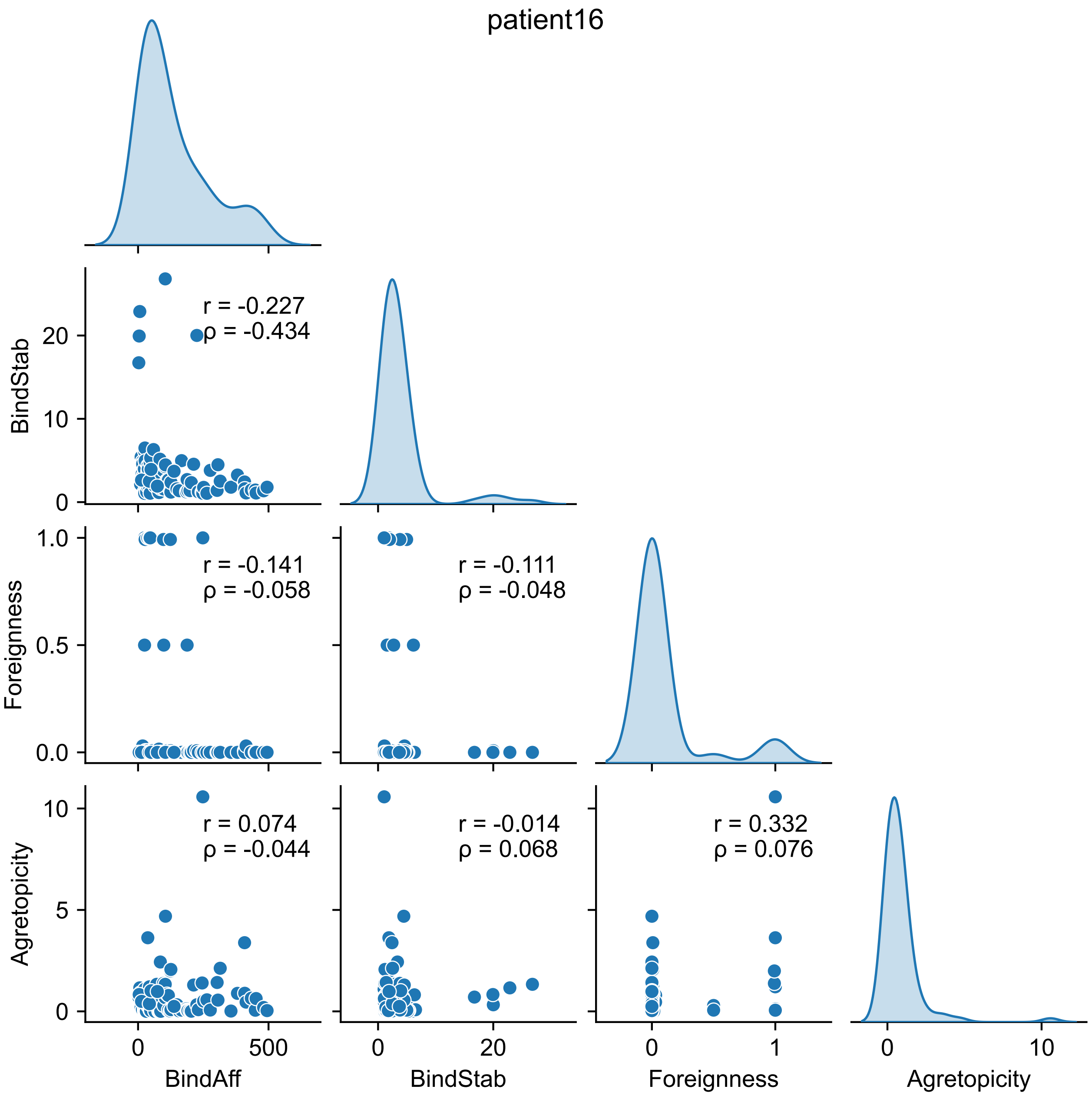

Figure S11 : Pairwise correlations between features of patient16. Pairwise correlations among binding affinity, binding stability, foreignness, and agretopicity of patient16. Each dot represents a candidate neoantigen. r represents Pearson's correlation coefficient. ρ represents Spearman's correlation coefficient.

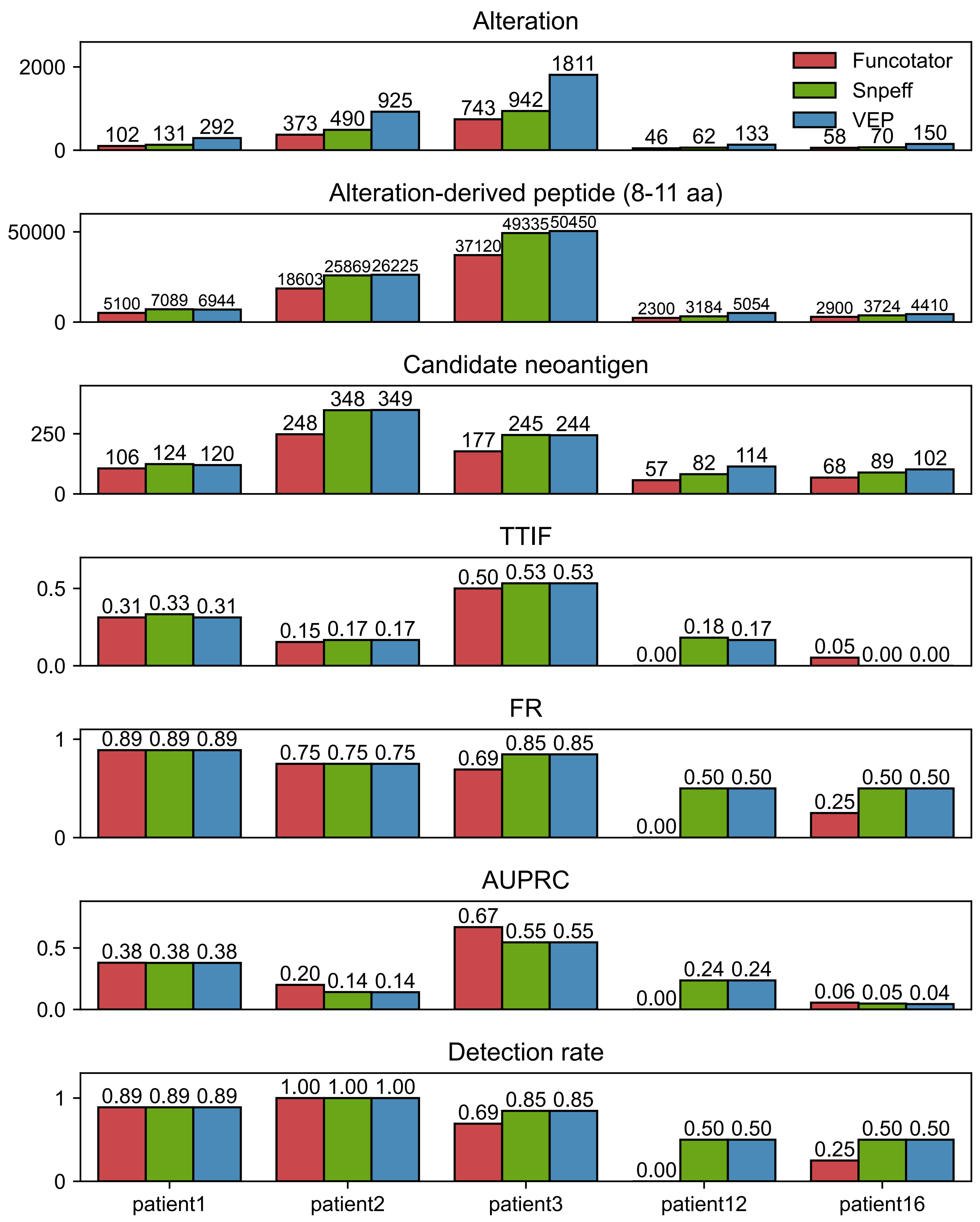

Figure S12 : Comparison between performance of multiple annotation models, including VEP, Snpeff, and Funcotator. From the top panel to bottom panel, the results are the number of SNV/indels, altaration-derived peptides, candidate neoantigens, TTIF, FR, AUPRC, and detection rate.

Table S1 : Comparison of neoantigen detection softwares

Method	Neoantigen source (alteration type)				Acceptable genomic data format	Acceptable transcriptomic data format	HLA typing	TCR decoding	Platform	Ref.
	SNV	Indel	Gene fusion	Aberrant splicing						
NeoHunter	✓	✓	✓	✓	fastq/bam/vcf	fastq/bam	✓	✓	Python	this work
pTuneos	✓	✓			fastq/vcf	fastq	✓		Python	[1]
pVAC-Seq	✓				vcf	bam			Python	[2]
Neopepsee	✓	✓			vcf	fastq	✓		Shell	[3]
Epi-Seq	✓				fastq				Shell	[4]
Antigen.garnish					peptide				R	[5]
CloudNeo	✓	✓			bam/vcf		✓		Web	[6]
DeepHLApan					peptide				Shell	[7]
ProTECT	✓	✓	✓		fastq	bam	✓		Python	[8]
Epidisco	✓	✓			fastq	fastq	✓		Shell	[9]
INTEGRATE-neo			✓		fastq	fastq	✓		Python	[10]
MuPeXI	✓	✓			vcf				Python	[11]
Neoantimon	✓	✓	✓		vcf	bam			R	[12]
NeoANT-HILL	✓	✓			vcf	fastq	✓		Python	[13]
NeoFlow	✓	✓			fastq/vcf		✓		Shell	[14]
ScanNeo		✓			vcf	bam	✓		Python	[15]
TSNAD	✓	✓			fastq				Shell	[16]
TIminer	✓	✓			vcf	fastq	✓		Shell	[17]

Table S1 (continued): Comparison of neoantigen detection softwares

Method	Neoantigen source (alteration type)				Acceptable genomic data format	Acceptable transcriptomic data format	HLA typing	TCR decoding	Platform	Ref.
	SNV	Indel	Gene fusion	Aberrant splicing						
NeoPredPipe	✓	✓			vcf				Python	[18]
EDGE					peptide				Web	[19]
ASNEO				✓		fastq			Python	[20]
Neoepiscope	✓	✓			fastq	fastq	✓		Python	[21]
NeoFuse			✓			fastq	✓		Shell	[22]
nf-core	✓	✓	✓		fastq/vcf	fastq	✓		Shell	[23]
OpenVax	✓	✓			fastq	fastq			Shell	[24]
ProGeo-neo	✓	✓	✓		fastq	fastq	✓		Python	[25]
Vaxrank	✓	✓			vcf	bam			Python	[26]
TruNeo	✓	✓	✓		fastq	fastq	✓		Shell	[27]
pVACtools	✓	✓			fastq	bam			Shell	[28]
neoFusion			✓			fastq	✓		Python	[29]

Note: blank cells represent that the corresponding function is not supported;

Table S2 : Number of valid alterations and alteration-derived peptides

Patient	Number of valid alteration				Number of alteration-derived peptides			
	SNV	indel	gene fusion	aberrant splicing	SNV	indel	gene fusion	aberrant splicing
patient1	286	6	1	45	6802	142	46	1460
patient2	907	18	1	57	25391	834	10	2683
patient3	1773	38	1	88	48618	1832	46	4002
patient12	107	26	1	18	3100	1954	174	631
patient16	128	22	1	25	3702	708	46	725

Table S3 : Ranked candidate neoantigens of the TESLA patients

patientID	MT_pep	WT_pep	BindAff	Quantification	BindStab	Foreignness	Agretopicity	AlterationType	Rank
patient2	FLDEGFSGL	FPDEGFSGL	2.92	1.2	2.62	0.500014968	0.000773953	SNV	1
patient2	FLSELEASV	SLSELEASV	3.21	38.3	8.05	4.60E-07	0.253754941	SNV	2
patient2	FLLWKATGV	FLSWKATGV	3.88	5.3	6.72	0.500044215	0.533700138	SNV	3
patient2	LLLDLLLFL	LPLDLLLFL	4.44	1.9	11.26	0.000118361	0.000759608	SNV	4
patient2	YLLVNIGFGV	YLLVNIGSGV	4.91	16.8	8.09	0.007620666	0.342160279	SNV	5
patient2	VLNFYTTFV	VLNFHTTFV	4.95	1.3	9.83	4.53E-07	0.453712191	SNV	6
patient2	FLDEGFSGLV	FPDEGFSGLV	5.05	1.2	4.94	0.500015081	0.000868027	SNV	7
patient2	YMMDEEGNQFV	GMMDEEGNQFV	5.24	35.2	8.42	0.000117905	0.224315068	SNV	8
patient2	KSFKEIKLW	KSFKKIKLW	5.85	1.9	49.34	0.500000004	0.724009901	SNV	9
patient2	FHLAELCIEV	LHLAELCIEV	5.93	10.7	1.1	6.12E-05	0.425089606	SNV	10
patient2	FLLQPVTPL	SLLQPVTPL	5.98	1.9	9.33	0.999941048	0.158326714	SNV	11
patient2	YQVKHMYQV	HQVKHMYQV	6.06	4.6	12.6	0.992380229	0.063662149	SNV	12
patient2	TLFHDLWKL	TLFHDPWKL	6.66	15.6	3.64	0.996175544	0.41136504	SNV	13
patient2	ALPPTVYEV	ALPPTVYEA	6.74	26.4	11.82	0.00762022	0.172731932	SNV	14
patient2	LLSLWVPQV	LLSLWAPQV	7.04	2.6	13.08	0.992439171	0.470273881	SNV	15
patient2	FLPERFCEA	PLPERFCEA	7.46	1.5	3.07	2.15E-10	0.002473786	SNV	16
patient2	KVWAFTEEV	KVWAGTEEV	7.51	5	11.82	1.81E-06	0.400747065	SNV	17
patient2	VLWGHRFFSV	VLWGHRFFPV	7.67	1.9	9.25	0.996175545	1.094151213	SNV	18
patient2	LSLAGSDCFW	LSLAVSDCFW	7.73	1.2	16.06	0.996190228	1.423572744	SNV	19
patient2	FLYELEEKMEV	FLHELEEKMEV	7.8	6.6	12.3	0.999941051	0.485376478	SNV	20
patient2	RLLSLWVPQV	RLLSLWAPQV	7.95	2.6	17.39	0.992439171	0.56745182	SNV	21
patient2	LLVNIGFGV	LLVNIGSGV	7.99	16.8	6.22	9.05E-07	0.152714067	SNV	22
patient2	YLLNDASSISV	YLLNDASLISV	8.22	3.2	8.27	5.94E-05	1.734177215	SNV	23
patient2	FTNREIDLY	FTDREIDLY	8.94	1	1.4	0.999941048	3	SNV	24
patient2	YLALPPTVYEV	YLALPPTVYEA	9.01	26.4	6.55	0.999999547	0.198676957	SNV	25
patient2	ALLLDLLLFL	ALPLDLLLFL	9.11	1.9	3	0.000118361	0.960970464	SNV	26
patient2	FSSPTHSLY	SSSPTHSLY	9.91	25.8	1.34	4.56E-07	0.29912466	SNV	27
patient2	GSARALSLW	GPARALSLW	9.95	2.2	8.86	0.007621112	0.006900091	SNV	28
patient2	QLLKFIPSL	QLLKFIPGL	9.95	9.7	6.12	0.999999547	0.872042068	SNV	29
patient2	FICWFSYEL	FICWFPYEL	10.13	40.7	7.07	0.801526527	0.958372753	SNV	30
patient2	YLLNDASSI	YLLNDASLI	10.77	3.2	3.3	4.53E-07	1.073778664	SNV	31
patient2	TLMEVMSKI	TLMEVMPKI	10.82	9.4	10.42	5.90E-05	2.12992126	SNV	32
patient2	ILYHGNLVV	ILYHGNPVV	10.97	1.7	6.74	0.000118845	1.196292257	SNV	33
patient2	YLAVDALEL	YLAADALEL	11.22	19.1	2.25	6.95E-09	1.803858521	SNV	34
patient2	FLYELEEKM	FLHELEEKM	11.3	6.6	1.42	0.500000114	0.322857143	SNV	35
patient2	YILYHGNLVV	YILYHGNPVV	11.33	1.7	2.84	0.000120645	0.799012694	SNV	36
patient2	GQFGKVWMA	GQFGEVWMA	11.65	4.3	1.26	0.007677839	0.782930108	SNV	37
patient2	LLLPGNNWKV	LLLPGNNRKV	12.19	132.2	6.46	6.03E-05	0.080563082	SNV	38
patient2	KLLEGQPVL	ELLEGQPVL	12.35	3.4	5.97	9.16E-07	0.032555687	SNV	39
patient2	YLLIHGTAV	YLLIHGTAD	12.93	3.3	5.6	4.56E-07	0.000980957	SNV	40
patient2	IVRNATFTW	TVRNATFTW	13.03	1.6	8.75	5.90E-05	0.472101449	SNV	41
patient2	KSLTHLFFVF	-	13.22	3.7	10.91	0.501912344	-	aberrant splicing	42
patient2	LSPPLRLASLW	SSPPLRLASLW	13.54	2	2.34	0.999941048	0.66372549	SNV	43
patient2	RTDKVRALW	RTDKVRALR	14.25	172	55.36	0.000117898	0.000809235	SNV	44
patient2	LLPGNNWKV	LLPGNNRKV	14.38	132.2	5.34	5.99E-05	0.009389058	SNV	45
patient2	KTKSFKEIKLW	KTKSFKKIKLW	14.74	1.9	25.87	0.500000004	0.414277684	SNV	46
patient2	VLWISAFMV	VLWIPAFMV	15.06	18	4.94	0.007619778	0.508611955	SNV	47
patient2	FIAEQILATL	SIAEQILATL	15.23	7.1	3.77	3.50E-09	0.142656426	SNV	48
patient2	IMVASLLPA	MMVASLLPA	15.45	1.3	3.87	0.000118351	2.001295337	SNV	49
patient2	FLAANRVENV	ILAANRVENV	15.52	1.6	8.98	3.53E-09	0.097036389	SNV	50
patient2	YMPGVGDFAV	HMPGVGDFAV	15.93	10.4	3.49	1.82E-06	0.066705749	SNV	51
patient2	GLYGNLIVL	GLSGNLIVL	16.03	8.5	3.15	1.37E-06	0.209899175	SNV	52
patient2	FLSEFAGTQL	FLPEFAGTQL	16.35	11.2	3.26	8.07E-11	0.363494887	SNV	53
patient2	FLREGSMFVV	FLREGSMFVA	16.88	6.7	2.61	0.5	0.264038792	SNV	54
patient2	TLFHDLWKLL	TLFHDPWKLL	17.03	15.6	1.31	0.9980878	0.492908828	SNV	55
patient2	FQLPILQPA	FQLPTLQPA	17.47	1.3	5.07	5.94E-05	0.671923077	SNV	56
patient2	QLFHVTYIL	QLFHVAYIL	18.32	1	3.57	0.999941497	0.585116576	SNV	57
patient2	HLYGIAESV	HLYGIAGSV	18.57	2.4	4.77	4.63E-07	0.163482701	SNV	58
patient2	KLLEGQPVLL	ELLEGQPVLL	18.93	3.4	5.8	9.19E-07	0.019084008	SNV	59
patient2	KLQKFIQWL	KLQKVIQWL	19.64	14.6	2.59	0.500000002	0.66893733	SNV	60

Table S3 (continued): Ranked candidate neoantigens of the TESLA patients

patientID	MT_pep	WT_pep	BindAff	Quantification	BindStab	Foreignness	Agretopicity	AlterationType	Rank
patient2	KTLGIKLSF	KTLGIKLPF	20.34	47.9	5.22	9.05E-07	0.643060386	SNV	61
patient2	LALQLWATCW	-	20.68	1.5	8.45	0.999999551	-	aberrant splicing	62
patient2	FLWGSRAHA	FLWGPRAHA	21.23	5.3	3.04	0.015124751	0.498825188	SNV	63
patient2	LLFKDSKLAV	LLFKDPKLAV	21.48	2.9	3.61	2.69E-11	0.786237189	SNV	64
patient2	FLFQDDLVQSI	FLSQDDLVQSI	21.7	2	8.89	3.53E-09	0.418999807	SNV	65
patient2	LLFFVEGLCFL	LLFFVEGLCFP	21.8	1.2	1.85	6.03E-05	0.008853906	SNV	66
patient2	FPYTTILFI	FPYTTILSI	21.91	30	7.68	5.90E-05	1.375392341	SNV	67
patient2	GAFKDPQFSW	GAFKDPQFPW	21.98	9.4	5.21	0.007735883	0.830374008	SNV	68
patient2	ALDKNLHQL	ALDENLHQL	22.15	1.5	4.64	4.60E-07	3.722689076	SNV	69
patient2	QLFSMCSSV	QLFPMCSSV	22.7	21	5.89	0.007619775	0.972996142	SNV	70
patient2	GLYGNLIVLSV	GLSGNLIVLSV	22.82	8.5	10.23	1.37E-06	0.274410774	SNV	71
patient2	GLLGGVSPM	GLLGGVGPM	22.85	3.9	1.92	0.999970523	0.257638967	SNV	72
patient2	LTSALWTW	-	22.88	1.5	9.11	0.022629278	-	aberrant splicing	73
patient2	HSRPSYCSASW	HSRPSYRSASW	23.48	8.3	3.83	0.999985261	0.677437969	SNV	74
patient2	KLLNCPCPA	KLLNRPCPA	23.8	3.4	5.27	0.999999547	0.287752388	SNV	75
patient2	SIIGQVPPL	SIIGQVPPS	24.47	3.4	2.79	0.999941048	0.009950229	SNV	76
patient2	KQLLKFIPSL	KQLLKFIPGL	25.69	9.7	1.07	0.999999547	0.924433249	SNV	77
patient2	IIVRNATFTW	ITVRNATFTW	25.92	1.6	5.25	5.90E-05	4.636851521	SNV	78
patient2	LLNDASSISV	LLNDASLISV	26.02	3.2	5.13	3.53E-09	2.671457906	SNV	79
patient2	LSTSGGSIFW	LSTSGGSISW	26.11	10.4	5.91	0.999999997	0.778009535	SNV	80
patient2	SLNAACYAL	SLNAARYAL	26.96	12.5	5.78	0.999999997	0.231535555	SNV	81
patient2	LVDIVLNFY	LVDIVLNFH	26.96	1.3	4.68	2.71E-11	0.00568425	SNV	82
patient2	LVLASNMDLLW	PVLASNMDLLW	27.07	7.8	3.17	4.56E-07	0.069301861	SNV	83
patient2	SLINSTVIV	SLINPTVIV	28.32	13.7	5.68	5.36E-11	0.466326363	SNV	84
patient2	FLSELEASVA	SLSELEASVA	28.62	38.3	1.55	4.60E-07	0.076848719	SNV	85
patient2	KLKEHLGFAV	KLKEHLGSAV	28.68	5.7	3.52	0.5	0.107419754	SNV	86
patient2	YLAVDALELM	YLAADALELM	28.73	19.1	1.46	6.95E-09	1.059756547	SNV	87
patient2	VVSKELPATW	-	29.67	3.7	5.9	4.70E-07	-	aberrant splicing	88
patient2	LLDNGTLTI	LLDNGTLTV	30.3	10.5	3.96	0.992380675	3.233724653	SNV	89
patient2	FLWGSRAHAEI	FLWGPRAHAEI	30.43	5.3	5.01	0.015124751	0.879479769	SNV	90
patient2	KSLAFYPKSF	-	31.97	1.5	10.02	0.000118351	-	indel	91
patient2	YLAVDALELMV	YLAADALELMV	33.32	19.1	4.84	7.01E-09	1.298013245	SNV	92
patient2	LALPPTVYEV	LALPPTVYEA	33.55	26.4	1.51	0.999999547	0.119259207	SNV	93
patient2	MMLQEQLDV	MMLQEQLDA	33.63	20.3	2.29	4.53E-07	0.064325472	SNV	94
patient2	VLSLYLNTTV	VLSPYLNTTV	34.98	9.6	7.23	5.90E-05	2	SNV	95
patient2	FQDDLVQSI	SQDDLVQSI	35.11	2	2.55	4.11E-13	0.075003738	SNV	96
patient2	YITKRIIAV	YITERIIAV	35.46	72.3	3.63	2.67E-11	4.511450382	SNV	97
patient2	LLAELPASVHV	LLAELPASVHA	35.62	32	8.59	0.500015079	0.087866006	SNV	98
patient2	KLADFGLACA	KLADFGLARA	36.14	1.6	6.18	5.94E-05	1.072721876	SNV	99
patient2	FLNQEVVETM	-	36.29	32	3.38	4.56E-07	-	aberrant splicing	100
patient2	GQLFHVTYI	GQLFHVAYI	36.41	1	2.6	0.992437851	1.0224656	SNV	101
patient2	KTSRYLSDLF	ETSRYLSDLF	36.67	1.9	4.87	0.500000004	0.013806476	SNV	102
patient2	KLLNCPCPAAV	KLLNRPCPAAV	38.02	3.4	12.93	1	0.839664311	SNV	103
patient2	SLLDNGTLTI	SLLDNGTLTV	38.61	10.5	3.43	0.992380682	3.146699267	SNV	104
patient2	LLNCPCPAAV	LLNRPCPAAV	39.56	3.4	6.29	1	0.8648885	SNV	105
patient2	ILAVEMTPL	ISAVEMTPL	39.61	9.5	1.45	3.56E-09	0.004875797	SNV	106
patient2	FLCSSTSEGL	FLRSSTSEGL	39.67	8.4	3.01	0.007621109	0.730974756	SNV	107
patient2	RLHSSLYFSL	RLHSSLHFSL	39.94	5	4.05	0.5	0.633063877	SNV	108
patient2	LLHSGVIGAV	LLHSGVMGAV	40.05	84.9	3.45	9.12E-07	1.108190371	SNV	109
patient2	KLEEIFHLA	KLEEILHLA	40.29	10.7	2.9	3.48E-09	1.342552483	SNV	110
patient2	ALWEAFYRQNL	ALREAFYRQNL	42.17	172	2.42	0.992380232	0.01611344	SNV	111
patient2	ISDGLVEIFW	ISDGLVEISW	42.2	8.3	5.45	7.06E-09	1.215437788	SNV	112
patient2	KLQKFIQWLL	KLQKVIQWLL	42.4	14.6	3.77	0.992380232	0.581937963	SNV	113
patient2	RSFSEVFLV	RSSSEVFLV	44.37	2.1	2.12	3.48E-09	0.076811218	SNV	114
patient2	FLPERFCEAL	PLPERFCEAL	44.42	1.5	3.51	5.99E-05	0.004912842	SNV	115
patient2	RLDGFFKNAV	RLDGSFKNAV	44.53	82.9	4.73	4.56E-07	0.367864519	SNV	116
patient2	FLKGEPKVFGV	FLKGEPKVLGV	44.76	18.9	1.44	2.73E-11	0.294280079	SNV	117
patient2	KIRHLLAQW	EIRHLLAQW	45.6	1.2	4.6	0.007619771	0.03078107	SNV	118
patient2	FLEGYIKAI	FSEGYIKAI	45.89	3.5	1.81	0.007619771	0.004115208	SNV	119
patient2	IMYGRIEISL	IMYGRIGISL	46.01	4	1.78	8.09E-11	0.780889341	SNV	120

Table S3 (continued): Ranked candidate neoantigens of the TESLA patients

patientID	MT_pep	WT_pep	BindAff	Quantification	BindStab	Foreignness	Agretopicity	AlterationType	Rank
patient2	ILFPAECGNLV	ILFPAECGNSV	46.87	2.5	4.83	9.05E-07	0.978088481	SNV	121
patient2	LTQGYIYFY	LTQGYIYFH	47.31	30.8	2.8	9.23E-07	0.00740203	SNV	122
patient2	TLFHDLWKLLI	TLFHDPWKLLI	47.37	15.6	3.21	0.999999547	0.472141932	SNV	123
patient2	GLFRVTPEIPV	GLFRVTPEIPA	47.69	2.9	5.37	1	0.105352685	SNV	124
patient2	SSCNTLGIDLW	SSSNTLGIDLW	48.12	2	3.75	0.999999547	0.730308089	SNV	125
patient2	MPYFTNREI	MPYFTDREI	48.56	1	3.42	5.94E-05	0.977062374	SNV	126
patient2	KKARMATGSW	-	48.88	8.3	1.02	5.94E-05	-	aberrant splicing	127
patient2	ILLQTVATI	IPLQTVATI	49.59	1.4	11.24	0.999941048	0.00256503	SNV	128
patient2	RLIYLSHVQV	-	50.46	3.7	11.04	9.05E-07	-	aberrant splicing	129
patient2	SSVYWCREDW	SPVYWCREDW	51.79	4	2.63	0.999941048	0.052852332	SNV	130
patient2	FGMDVIFRL	FGMDAIFRL	54.4	12.5	1.69	3.48E-09	0.540540541	SNV	131
patient2	SLTHLFFV	-	54.98	3.7	9.91	0.007620227	-	aberrant splicing	132
patient2	STSGGSIFW	STSGGSISW	55.87	10.4	2.65	0.999941048	0.845874338	SNV	133
patient2	NMTVLSLYL	NMTVLSPYL	56.54	9.6	1.12	5.90E-05	0.69596258	SNV	134
patient2	MLQEQLDVI	MLQEQLDAI	56.57	20.3	2.31	4.53E-07	3.600891152	SNV	135
patient2	KSFKEIKLWTM	KSFKKIKLWTM	56.64	1.9	5.75	0.500000006	0.605257534	SNV	136
patient2	ISLFSPRHTF	ISPFSPRHTF	57.22	5.5	4.52	0.007677832	0.628376894	SNV	137
patient2	RLTHELLTAL	RLTHESLTAL	59.19	19.6	4.29	0.992380232	0.407560421	SNV	138
patient2	HLIGGIQVL	HSIGGIQVL	59.48	3.3	1.33	0.00011881	0.005755251	SNV	139
patient2	VTSDCGYEIIW	VTSDRGYEIIW	60.82	5	7.19	0.999999547	1.101213109	SNV	140
patient2	FIWLATRRV	SIWLATRRV	61.95	30	2.51	0.007620227	0.153474545	SNV	141
patient2	YISDDIPIPA	YIPDDIPIPA	63.34	2.5	2.04	4.67E-07	0.401750603	SNV	142
patient2	KAVRAKAKVAW	KAVRAKAKVAR	63.46	13.3	13.37	4.53E-07	0.002121494	SNV	143
patient2	ILPGVFSKL	IPPGVFSKL	63.81	27.2	1.57	8.17E-11	0.003407786	SNV	144
patient2	HLSHSDTYV	HLSHSDAYV	65.05	9.7	3.94	6.98E-09	2.040464241	SNV	145
patient2	CVDWLIAVY	CVDWSIAVY	65.22	127.2	2.28	1.07E-10	1.963866305	SNV	146
patient2	SLIGGTMKLL	-	65.28	71.4	1.32	0.500014968	-	aberrant splicing	147
patient2	KIMNKLRYI	KIMDKLRYI	65.65	1.6	7.6	3.48E-09	2.984090909	SNV	148
patient2	ALPPTVYEVV	ALPPTVYEAV	66.1	26.4	4.18	0.007620666	1.130300958	SNV	149
patient2	YILDRESPL	YILGRESPL	66.41	3.1	1.54	0.000295145	0.346191941	SNV	150
patient2	YLDENKKVV	YLDENEKVV	67.11	1.8	2.61	4.53E-07	2.370540445	SNV	151
patient2	VVFGSMQMAV	AVFGSMQMAV	67.33	10.8	2.22	3.53E-09	1.614628297	SNV	152
patient2	ATRTDKVRALW	ATRTDKVRALR	68.18	172	4.39	0.007677829	0.002286226	SNV	153
patient2	MLGPAKWQNV	MSGPAKWQNV	69.16	3.5	1.72	6.03E-05	0.008568887	SNV	154
patient2	HISRILPGV	HISRIPPGV	74.63	27.2	6.29	8.09E-11	0.101789465	SNV	155
patient2	VIYFQGFCV	VIYFQGFRV	75.3	1.4	2.02	1	0.607405017	SNV	156
patient2	SSLYFSLPLF	SSLHFSLPLF	76.09	5	1.51	1.37E-06	1.261020882	SNV	157
patient2	LGFWPEACW	LGFRPEACW	76.82	7.2	1.09	1	1.59047619	SNV	158
patient2	LIADFIAEQI	LIADSIAEQI	76.95	7.1	2.88	3.48E-09	0.160680727	SNV	159
patient2	LLVDVPPEEL	LLVDVPPEEP	77.67	9.2	1.59	0.007619778	0.007709594	SNV	160
patient2	ILDTAGHEEY	ILDTAGQEEY	78.06	51.2	1.8	5.90E-05	0.915552428	SNV	161
patient2	SSDCSAWYM	SSDRSAWYM	79.12	37.2	1.97	1	0.768304525	SNV	162
patient2	LIGMDLVAV	LIGMDPVAV	79.26	24.8	1.75	4.53E-07	0.158216225	SNV	163
patient2	FQALDKNLHQL	FQALDENLHQL	79.63	1.5	1.04	4.60E-07	4.765409934	SNV	164
patient2	SLYFNSCQI	SLYFNGCQI	80.48	1.2	3.03	2.69E-11	0.318556048	SNV	165
patient2	VSRLHSSLYF	VSRLHSSLHF	81.93	5	1.18	5.90E-05	0.556400679	SNV	166
patient2	RIVPSLTYQV	GIVPSLTYQV	82.97	1.7	7.95	4.53E-07	0.703731976	SNV	167
patient2	RSFDYEQFY	RSFDYEQFH	84.77	1.2	10.22	4.19E-13	0.030430194	SNV	168
patient2	KLEEIFHL	KLEEILHL	84.8	10.7	2.41	3.48E-09	0.184083706	SNV	169
patient2	LLHSGVIGA	LLHSGVMGA	86.77	84.9	2.07	4.60E-07	1.016518276	SNV	170
patient2	KSLTHLFFVFF	-	87.41	2.4	11.9	0.668364312	-	aberrant splicing	171
patient2	FLPKQGQIFCL	FLPKQGQIFCS	89.12	12.7	2.67	3.48E-09	0.028632196	SNV	172
patient2	FIAEQILAT	SIAEQILAT	89.2	7.1	1.56	2.71E-11	0.056760334	SNV	173
patient2	YQASSPNEV	YQASSPDEV	90.24	1.8	1.42	7.01E-09	0.696995443	SNV	174
patient2	GMIAQCTVKV	GMIAQCTVEV	91.19	1.5	6.71	2.67E-11	3.170723227	SNV	175
patient2	KGAFKDPQFSW	KGAFKDPQFPW	91.95	9.4	3.39	0.007735883	0.542445873	SNV	176
patient2	ELYQYLLQL	ELYQYLLQP	94.56	2.8	1.48	0.036971987	0.014190442	SNV	177
patient2	VPVPVPVLV	VPVPVPVPV	94.7	2.6	1.52	0.992380229	0.492254912	SNV	178
patient2	KLSMLKVLNL	KLPMLKVLNL	94.96	1.9	1.67	5.90E-05	0.682232919	SNV	179
patient2	KVFSCHSYL	KVFSRHSYL	95.17	3.8	1.71	7.22E-09	0.172615809	SNV	180

Table S3 (continued): Ranked candidate neoantigens of the TESLA patients

patientID	MT_pep	WT_pep	BindAff	Quantification	BindStab	Foreignness	Agretopicity	AlterationType	Rank
patient2	YAVGLENVW	YAVGLGNVW	95.35	1.4	1.3	5.48E-11	0.734026174	SNV	181
patient2	KSFRWASGI	KSFRWASGL	95.81	2.4	2.23	5.90E-05	0.689776818	SNV	182
patient2	YILYHGNLV	YILYHGNPV	96.53	1.7	2.08	0.000119743	2.551678562	SNV	183
patient2	LLLEKEVSRL	LLLGKEVSRL	97.24	1.6	2.28	6.98E-09	0.379784409	SNV	184
patient2	KAFYPKSLAF	-	99.89	1.5	3.19	3.66E-09	-	indel	185
patient2	TSDCGYEIIW	TSDRGYEIIW	100.59	5	1.34	0.999999547	1.411789474	SNV	186
patient2	LAGAAPTWW	PAGAAPTWW	101.33	1.2	1.17	0.75143152	0.033768675	SNV	187
patient2	SIIVRNATFTW	SITVRNATFTW	105.23	1.6	1.73	5.90E-05	3.084114889	SNV	188
patient2	RLMDSAQVA	RFMDSAQVA	106.29	5	3.14	2.69E-11	0.008767697	SNV	189
patient2	RSREEPYYY	RSREEPYHY	106.61	12.7	17.05	4.53E-07	0.500117277	SNV	190
patient2	ILKCLSHHV	TLKCLSHHV	106.92	9	3.03	0.000117901	0.258886199	SNV	191
patient2	GVVEAALPGV	GAVEAALPGV	107.05	2	1.72	5.90E-05	0.087070038	SNV	192
patient2	FQPDAFLLQPV	FQPDASLLQPV	107.33	1.9	4.1	3.48E-09	1.203251121	SNV	193
patient2	KLTYSLETL	KLTYPLETL	107.7	5.3	5.01	4.56E-07	0.636186426	SNV	194
patient2	YLDENKKVVL	YLDENEKVVL	108.36	1.8	1.97	4.53E-07	1.913473424	SNV	195
patient2	KLCAWPVSEL	KLCAWLVSEL	109.64	21.2	3.21	4.70E-07	2.834539814	SNV	196
patient2	LLWKATGVTA	LSWKATGVTA	110.93	5.3	2.01	0.501956105	0.018779096	SNV	197
patient2	STVFHSCCPGW	PTVFHSCCPGW	110.99	2.1	3.87	0.022629727	0.070474763	SNV	198
patient2	SLFSPRHTFL	SPFSPRHTFL	115.84	5.5	1.46	0.007677832	0.005435892	SNV	199
patient2	KLEEIFHLAEL	KLEEILHLAEL	116.42	10.7	2.29	3.48E-09	0.820379114	SNV	200
patient2	SLLFKDSKLAV	SLLFKDPKLAV	117.65	2.9	1.77	9.05E-07	0.819974909	SNV	201
patient2	ELIGMDLVAV	ELIGMDPVAV	121.2	24.8	1.31	5.90E-05	0.135302589	SNV	202
patient2	IVFSPGSVSV	MVFSPGSVSV	121.65	1.4	1.18	0.501956104	2.367192061	SNV	203
patient2	RSFSEVFLV	RSSSEVFLV	122.03	2.1	2.54	3.48E-09	0.270642507	SNV	204
patient2	SARALSLW	PARALSLW	122.66	2.2	1.92	0.007621112	0.046911871	SNV	205
patient2	LSLESGFSAF	LSLESGFPAF	123.05	1.9	1.54	0.007620217	0.819022897	SNV	206
patient2	SLIGGTMKLLL	-	123.29	71.4	2.09	0.500015082	-	aberrant splicing	207
patient2	ATFPYTTILF	ATFPYTTILS	124.01	30	3.45	0.022572515	0.013366647	SNV	208
patient2	ILDRESPLSLY	ILGRESPLSLY	125.13	3.1	2.14	0.007677829	0.094774633	SNV	209
patient2	VVLNYKFEI	VVLNHKFEI	125.23	2.7	1.93	0.007619781	0.196183792	SNV	210
patient2	AGFGSPARW	AGFGSPAGW	129.23	58.4	1.13	0.007619781	0.615205179	SNV	211
patient2	LLDLLLFL	PLDLLLFL	129.28	1.9	2.71	1.40E-08	0.018709984	SNV	212
patient2	ALNSPHSEFFV	ALNSPHSESFV	129.87	81.1	3.95	0.015124741	0.066459583	SNV	213
patient2	QLTACPLHL	QLTARPLHL	133.56	19.3	1.64	1.05E-08	0.072050494	SNV	214
patient2	SLFLFEHQRI	SSFLFEHQRI	134.52	2.7	1.95	2.71E-11	0.016734903	SNV	215
patient2	NLATAPTHV	SLATAPTHV	135.01	1.5	1.33	5.94E-05	3.033250955	SNV	216
patient2	WTWPAAAW	-	137.26	1.5	1.96	0.996175656	-	aberrant splicing	217
patient2	LLGGVSPMHSV	LLGGVGPMHSV	137.3	3.9	5.04	0.999941048	0.919009371	SNV	218
patient2	KTLVSCCLY	KTLVPCCLY	139.1	3.6	12.46	0.500000682	0.640040491	SNV	219
patient2	HLYGIAESVNV	HLYGIAGSVNV	139.68	2.4	4.38	4.63E-07	1.3362671	SNV	220
patient2	IGMDLVAVFRW	IGMDPVAVFRW	140.63	24.8	3.28	0.007794379	0.992939349	SNV	221
patient2	SALWTWPAAAW	-	142.04	1.5	3.25	0.999999997	-	aberrant splicing	222
patient2	IAADGEFLY	IAADGEFLH	142.32	6.6	2.34	2.71E-11	0.009576158	SNV	223
patient2	RLDGFFKNAVV	RLDGSFKNAVV	144.77	82.9	3.41	4.56E-07	0.290691137	SNV	224
patient2	LVQETLFHDLW	LVQETLFHDPW	145.97	15.6	3.35	4.74E-07	0.733075532	SNV	225
patient2	LLIHGTAVDNV	LLIHGTADDNV	146.7	3.3	7.22	3.48E-09	0.872954478	SNV	226
patient2	VQFGRHPTV	VQSGRHPTV	154.49	2.7	1.67	0.007620663	0.074374158	SNV	227
patient2	ITKRIIAVSF	ITERIIAVSF	155.74	72.3	1.68	4.53E-07	0.379825866	SNV	228
patient2	KTLGIKLSFL	KTLGIKLPFL	156.59	47.9	2.3	9.05E-07	1.143827611	SNV	229
patient2	FLLWKATGVTA	FLSWKATGVTA	157.52	5.3	3.58	0.501956105	0.296826713	SNV	230
patient2	TVVSKELPATW	-	158.36	3.7	1.73	4.70E-07	-	aberrant splicing	231
patient2	KMAIPVLEA	EMAIPVLEA	160.77	6	1.25	1.82E-16	0.050788827	SNV	232
patient2	ILLEAPGKCV	ILSEAPGKCV	162.6	33.9	1.71	4.53E-07	0.172954804	SNV	233
patient2	TSSVYWCREDW	TSPVYWCREDW	163.84	4	1.78	0.999941048	0.338323662	SNV	234
patient2	SSDCSAWY	SSDRSAWY	164.79	37.2	2.48	0.999999547	0.693940287	SNV	235
patient2	KLAPLRVSQL	KSAPLRVSQL	165.21	1	2.08	2.27E-06	0.010052425	SNV	236
patient2	FLFQDDLV	FLSQDDLV	165.22	2	7.81	3.53E-09	0.123647304	SNV	237
patient2	CALALQLW	-	165.59	1.5	6.25	3.58E-09	-	aberrant splicing	238
patient2	KTLMEVMSKI	KTLMEVMPKI	167.25	9.4	1.99	5.94E-05	1.075701055	SNV	239
patient2	RLTHELLTA	RLTHESLTA	168.83	19.6	4.68	5.94E-05	0.146074512	SNV	240

Table S3 (continued): Ranked candidate neoantigens of the TESLA patients

patientID	MT_pep	WT_pep	BindAff	Quantification	BindStab	Foreignness	Agretopicity	AlterationType	Rank
patient2	RLMDSAQVAHW	RFMDSAQVAHW	170.86	5	13.37	5.40E-11	0.116411057	SNV	241
patient2	VPFTGELSV	VPSTGELSV	170.98	49.3	1.9	0.015181927	0.095868751	SNV	242
patient2	CPFKQDASI	CPFEQDASI	174.51	26	1.38	4.60E-07	1.564130143	SNV	243
patient2	RILPGVFSKL	RIPPGVFSKL	175.53	27.2	1.91	7.06E-09	0.068538562	SNV	244
patient2	LLSPEKFVQI	LLSPEKSVQI	177.03	24.8	2.96	5.38E-11	0.331927101	SNV	245
patient2	FPPSHIFFV	FPPSHIFSV	178.15	4.1	1.85	9.05E-07	1.036659878	SNV	246
patient2	IIPGFPYPTAV	IIPGFPYPTAA	178.92	1.2	1.87	0.501941596	0.07322883	SNV	247
patient2	VPVLVAPAV	VPVPVAPAV	180.17	2.6	2.5	1.38E-06	1.718851364	SNV	248
patient2	IGYAVGLENVW	IGYAVGLGNVW	183.73	1.4	1.33	5.90E-05	0.690247201	SNV	249
patient2	RVVDGSRHSNW	RAVDGSRHSNW	184.04	16.9	8.27	0.015124302	2.229706809	SNV	250
patient2	KLSEILIQNT	NLSEILIQNT	184.46	1.2	1.65	4.53E-07	0.216578607	SNV	251
patient2	LLDLNSQSI	LLDLNPQSI	185.82	4.4	1.31	1.04E-08	0.808721765	SNV	252
patient2	KLLEGPVPEGA	-	185.83	19.5	1.77	0.000237578	-	aberrant splicing	253
patient2	KSDSVTSDCGY	KSDSVTSDRGY	191.79	5	1.85	0.503809887	0.679744817	SNV	254
patient2	KLGFKHFSEA	KLGFKHFPEA	193.35	1.5	1.14	2.73E-11	1.83583365	SNV	255
patient2	LLHSGVIGAVV	LLHSGVMGAVV	194.35	84.9	3.35	6.03E-05	1.052930978	SNV	256
patient2	ATSAEKDSVLY	ATSAEKDSVLN	196.19	3.8	1.51	0.007620227	0.006197342	SNV	257
patient2	ITIPDGFTI	TTIPDGFTI	197.8	3.4	2.43	9.09E-07	0.245595302	SNV	258
patient2	IPGFPYPTAV	IPGFPYPTAA	200.82	1.2	1.42	0.501941596	0.053611616	SNV	259
patient2	LSCAPPPW	-	202.25	37.9	12.89	0.02251618	-	indel	260
patient2	AADVRTFLEGY	AADVRTFSEGY	202.3	3.5	1.76	0.007619778	0.260669003	SNV	261
patient2	KAAVRTQW	-	204.52	8.8	19.76	0.029797924	-	aberrant splicing	262
patient2	LMDSAQVAHW	FMDSAQVAHW	207.15	5	1.32	2.73E-11	0.508193906	SNV	263
patient2	ALSTSGGSIFW	ALSTSGGSISW	208.25	10.4	1.09	0.999999997	0.862318841	SNV	264
patient2	QAMEITLGM	QAMGITLGM	209.65	2.6	1.01	6.95E-09	1.038951385	SNV	265
patient2	KLKEHLGFA	KLKEHLGSA	209.69	5.7	2.34	0.5	0.462820315	SNV	266
patient2	GLFQALDKNL	GLFQALDENL	210.37	1.5	1.13	4.53E-07	2.163410119	SNV	267
patient2	GLGNTIQAI	GLGKTIQAI	210.84	1.3	2.07	5.90E-05	0.544609185	SNV	268
patient2	GMDVIFRL	GMDAIFRL	213.71	12.5	1.2	3.48E-09	0.583540398	SNV	269
patient2	KLGAGQFGKV	KLGAGQFGEV	218.74	4.3	1.54	0.992380229	1.760624598	SNV	270
patient2	TLQKEVMEA	TLQEEVMEA	222.17	29.9	1	2.69E-11	4.286513602	SNV	271
patient2	TSQRREFFLY	TSQRRESFLY	223.45	5.1	1.03	2.67E-11	1.462464821	SNV	272
patient2	RLLGEAAEASL	-	223.62	2.4	1.21	6.03E-05	-	indel	273
patient2	SLYLNTTVL	SPYLNTTVL	225.58	9.6	2	5.94E-05	0.009550907	SNV	274
patient2	MLYLDENKKV	MLYLDENEKV	226.32	1.8	1.32	4.53E-07	6.082235958	SNV	275
patient2	FLLRENHEC	FLLRGNHEC	227.19	10.1	2.15	3.48E-09	1.42331788	SNV	276
patient2	SPVPRFLEI	SPLPRFLEI	227.93	1	1.49	5.90E-05	1.1587697	SNV	277
patient2	ISDILLWY	ISGILLWY	229.14	44.4	3.55	0.500000119	0.026733651	SNV	278
patient2	YLTSSELHKEL	YLTSPELHKEL	230.55	8	1.98	6.95E-09	0.569217095	SNV	279
patient2	FSSPTHSLYVF	SSSPTHSLYVF	231.73	25.8	1.34	4.60E-07	1.030002667	SNV	280
patient2	KLADFGLAC	KLADFGLAR	231.84	1.6	2.16	5.94E-05	0.050214969	SNV	281
patient2	ILNNGTTCRV	ILNNGKTCRV	235.22	1.4	10.19	3.48E-09	0.503974461	SNV	282
patient2	YLKKNKFLEAL	YLKKNRFLEAL	236.33	16.4	1.98	3.48E-09	0.907251718	SNV	283
patient2	RVSPTLNIFF	RVSPTLNIFI	238.04	1	3.01	0.50001508	0.170922251	SNV	284
patient2	VLGASTCSL	ALGASTCSL	240.03	6.7	1.6	4.53E-07	0.91659984	SNV	285
patient2	KQDASITHYL	EQDASITHYL	241.73	26	1.38	1.04E-08	0.040022053	SNV	286
patient2	SCCELTSALW	-	241.76	1.5	1.02	0.507576453	-	aberrant splicing	287
patient2	RTHTGEKLF	RTHTGEKPF	244.6	4.8	4.64	3.48E-09	0.219879004	SNV	288
patient2	RAGEAWIW	-	247.51	1.5	14.95	0.007677832	-	aberrant splicing	289
patient2	KAFYPKSLAFY	-	248.76	1.5	6.27	9.12E-07	-	indel	290
patient2	YTTILFIW	YTTILSIW	253.02	30	2.07	0.5	1.121741444	SNV	291
patient2	SVYWCREDW	PVYWCREDW	257.5	4	1.52	0.999941048	0.087055005	SNV	292
patient2	KLLEGQPV	ELLEGQPV	260.66	3.4	10.14	9.16E-07	0.028966181	SNV	293
patient2	LPLPQGSASI	-	267.95	39.5	6.64	1	-	aberrant splicing	294
patient2	SLTSSVSTL	SPTSSVSTL	270.63	11	2.35	0.503809897	0.009211237	SNV	295
patient2	FLLQPVTPLGT	SLLQPVTPLGT	270.87	1.9	1.37	0.999941048	0.132847138	SNV	296
patient2	RSGLKRGKHAW	RSGPKRGKHAW	272.52	20.4	4.17	0.992380229	1.415467719	SNV	297
patient2	KIYQVKHMYQV	KIHQVKHMYQV	275.16	4.6	6.09	0.992380685	0.173891062	SNV	298
patient2	ISNQDFRAF	ISNQDLRAF	277.36	1	1.09	2.69E-11	0.916286753	SNV	299
patient2	FSSPTHSLY	SSSPTHSLY	278.04	25.8	1.67	4.56E-07	0.571792868	SNV	300

Table S3 (continued): Ranked candidate neoantigens of the TESLA patients

patientID	MT_pep	WT_pep	BindAff	Quantification	BindStab	Foreignness	Agretopicity	AlterationType	Rank
patient2	MLNDSVLWISA	MLNDSVLWIPA	278.54	18	1.59	6.12E-05	2.47965815	SNV	301
patient2	ISVATFLPY	LSVATFLPY	279.43	4.8	1.53	5.34E-11	1.02754284	SNV	302
patient2	ISVKRTIYSL	ISVKGTIYSL	285.39	1	1.21	3.48E-09	0.70336414	SNV	303
patient2	MKIRHLLAQW	MEIRHLLAQW	286.44	1.2	1.12	0.007619771	1.057091191	SNV	304
patient2	ILLQETTRA	ISLQETTRA	287.84	1.5	2.47	2.71E-11	0.012340063	SNV	305
patient2	FQDSGRIVAI	FQDPGRIVAI	294.27	8.9	1.17	0.5	0.940670652	SNV	306
patient2	RTTDSLPGKF	RATDSLPGKF	297.82	5.6	2.88	5.94E-05	0.332990451	SNV	307
patient2	NLLDLNSQSI	NLLDLNPQSI	297.88	4.4	1.54	4.60E-07	2.756616694	SNV	308
patient2	YILDRESPLSL	YILGRESPLSL	302.12	3.1	1.38	0.000295145	0.910356465	SNV	309
patient2	KLHPDGSPDV	-	311.21	2	2.16	0.00762067	-	indel	310
patient2	LPPTVYEVV	LPPTVYEAV	313.58	26.4	1.74	4.56E-07	0.569710403	SNV	311
patient2	RLMDSAQV	RFMDSAQV	316.42	5	18.01	2.69E-11	0.015213202	SNV	312
patient2	GLATPPYPHLL	GLATPPYPHLP	320.02	7.8	1.52	0.500000454	0.066065781	SNV	313
patient2	SSIAADGEFLY	SSIAADGEFLH	322.64	6.6	1	1.07E-10	0.015698486	SNV	314
patient2	KLSTLCPKA	KPSTLCPKA	328.36	9.2	2.2	0.015124302	0.009953609	SNV	315
patient2	RCVDWLIAV	RCVDWSIAV	330.27	127.2	2.41	1.07E-10	0.351852641	SNV	316
patient2	LTHRRSGRFLF	LTHRRSGRFLS	335.91	2	1.88	3.58E-09	0.019369258	SNV	317
patient2	FPAECGNLV	FPAECGNSV	337.72	2.5	3.06	4.53E-07	0.753620601	SNV	318
patient2	IVYPTPTPI	IVHPTPTPI	338.96	4.4	1.53	7.03E-09	0.098000156	SNV	319
patient2	YSFGMDVIF	YSFGMDAIF	341.52	12.5	1.29	1.03E-12	1.310815998	SNV	320
patient2	GLYGNLIV	GLSGNLIV	348.99	8.5	5.13	1.37E-06	0.15615883	SNV	321
patient2	KARLIIWNLSF	KARLIIRNLSF	349.65	2.8	1.74	0.007677839	0.564051687	SNV	322
patient2	VPGFPYPTAV	VPGFPYPTAA	350.91	13.3	1.18	0.501927083	0.065882013	SNV	323
patient2	RIFSSPTHSLY	RISSSPTHSLY	351.85	25.8	2.71	0.992380229	0.962022202	SNV	324
patient2	KVIEAIAEC	EVIEAIAEC	357.28	15.2	2.07	2.07E-13	0.038473425	SNV	325
patient2	ISDDIPIPAEF	IPDDIPIPAEF	359.84	2.5	2.03	0.00017684	0.018744195	SNV	326
patient2	ASVHVLTGV	ASVHALTGV	371.06	32	1.12	5.34E-11	0.411790165	SNV	327
patient2	LTACPLHLHI	LTARPLHLHI	373.65	19.3	1.01	2.79E-08	0.95204729	SNV	328
patient2	KSLTHLFF	-	375.56	2.4	14.41	4.60E-07	-	aberrant splicing	329
patient2	LPATWPLRLI	-	375.91	3.7	1.9	1	-	aberrant splicing	330
patient2	RLIYLSHVQVL	-	376.75	3.7	4.51	9.05E-07	-	aberrant splicing	331
patient2	YAMYSRIF	YAMYSRIS	383.68	25.8	1.03	2.08E-13	0.02314031	SNV	332
patient2	QLNQNLIKEV	QLNRNLIKEV	387.07	1.2	5.3	1.33E-10	0.625800297	SNV	333
patient2	ISVATFLPY	LSVATFLPY	387.18	4.8	1.97	5.34E-11	1.752342159	SNV	334
patient2	SIFLKKHLV	STFLKKHLV	391.39	17.4	1.11	1.36E-06	0.150226074	SNV	335
patient2	IASGPTVASF	IASGPTVASS	391.82	1.3	1.33	0.007620217	0.027136191	SNV	336
patient2	NSQNKTSSVYW	NSQNKTSPVYW	394.01	4	2.4	4.53E-07	0.685759538	SNV	337
patient2	ATSQRREFFLY	ATSQRRESFLY	395.04	5.1	2.03	2.67E-11	1.72130719	SNV	338
patient2	KIPPITNIFFL	KIPPITNIFPL	398.25	1.5	1.88	0.007622007	0.821794845	SNV	339
patient2	CLAGAAPTW	CPAGAAPTW	400.52	1.2	1.24	0.503810111	0.181744753	SNV	340
patient2	HSIHSAIHLI	HSIHSAIHSI	407.68	3.3	1.28	4.60E-07	0.547471329	SNV	341
patient2	LTTTDRHVLF	LTTTDRHVLS	413.53	3.4	1.42	0.992380229	0.02369813	SNV	342
patient2	AQMKGVLLRW	ARMKGVLLRW	425.8	1.2	2.34	0.80031138	0.663084949	SNV	343
patient2	RSSSSSASPSF	RSSSSSASPSS	425.88	8.1	4.97	3.53E-09	0.025364539	SNV	344
patient2	KSYSSSCNTL	KSYSSSSNTL	426.81	2	2.79	0.999999547	0.707810945	SNV	345
patient2	RMLGPAKWQNV	RMSGPAKWQNV	429.9	3.5	1.37	6.03E-05	0.166191815	SNV	346
patient2	KARLIIWNL	KARLIIRNL	430.37	2.8	1.04	0.007677836	0.269403877	SNV	347
patient2	FLQIVSKLA	FLRIVSKLA	434.03	18.9	1.01	3.48E-09	0.123337264	SNV	348
patient2	ILSPPLRLASL	ISSPPLRLASL	443.37	2	1.73	0.999941048	0.020822404	SNV	349
patient2	FPERSFSEV	FPERSSSEV	443.6	2.1	1.16	4.67E-07	0.31822323	SNV	350
patient2	ILSPPLRLA	ISSPPLRLA	446.67	2	1.47	0.999941048	0.028352691	SNV	351
patient2	SSPNEVALVQW	SSPDEVALVQW	448.45	1.8	1.36	4.56E-07	0.941390096	SNV	352
patient2	VVEAALPGV	AVEAALPGV	453.17	2	1.09	3.53E-09	1.315213606	SNV	353
patient2	TLLSPEKFVQI	TLLSPEKSVQI	459.59	24.8	1.02	8.07E-11	0.449164883	SNV	354
patient2	VADKKARLIIW	VADKKARLIIR	464.31	2.8	1.97	0.022516616	0.014287015	SNV	355
patient2	LPPTVYEV	LPPTVYEA	469.36	26.4	1.36	3.61E-09	0.0527907	SNV	356
patient2	NTGNMTVLSLY	NTGNMTVLSPY	470.99	9.6	1.28	4.53E-07	0.63734286	SNV	357
patient2	GSCRSLVW	-	487.37	11.4	4.19	9.05E-07	-	aberrant splicing	358
patient2	RLQPKLEAML	-	488.97	32.3	1.51	1.23E-12	-	aberrant splicing	359
patient2	HLLVDVPPEEL	HLLVDVPPEEP	491.32	9.2	1.19	0.007619778	0.023642955	SNV	360

Table S3 (continued): Ranked candidate neoantigens of the TESLA patients

patientID	MT_pep	WT_pep	BindAff	Quantification	BindStab	Foreignness	Agretopicity	AlterationType	Rank
patient2	LVPGFPYPTAV	LVPGFPYPTAA	495.07	13.3	1.37	0.501941707	0.098143078	SNV	361
patient2	VMAENAAEV	VMAENAAGV	10.15	1	6.02	4.13E-13	0.834703947	SNV	362
patient2	KMLNVARLNV	-	13.31	2	2.48	1.60E-15	-	indel	363
patient2	KLSFLVMII	KLPFLVMII	28.39	47.9	3.25	2.07E-13	0.349975345	SNV	364
patient2	YLSHVQVLNA	-	42	3.7	4.02	7.94E-15	-	aberrant splicing	365
patient2	MIAQCTVKV	MIAQCTVEV	50.77	1.5	4.87	3.40E-15	3.237882653	SNV	366
patient2	VLFSNESSL	VLSSNESSL	160.86	3.4	1.39	4.10E-13	0.161442809	SNV	367
patient2	LIYFYVITI	LIYFYVTTI	172.68	3.4	2.61	6.18E-13	2.098432373	SNV	368
patient2	FQDDLVQSIGV	SQDDLVQSIGV	185.22	2	2.06	6.18E-13	0.142661285	SNV	369
patient2	FLKNNHLAI	FLKDNHLAI	188.4	5.4	1.61	8.21E-13	3.724792408	SNV	370
patient2	LTIREASVF	LTVREASVF	201.38	10.5	1.89	4.76E-15	0.528237547	SNV	371
patient2	FAVDVAEY	SAVDVAEY	228.96	5.7	1.27	3.16E-15	0.100910113	SNV	372
patient2	KTFYRKSFLTI	KTFHRKSFLTI	262.5	3.5	1.3	1.27E-14	1.26934236	SNV	373
patient2	YLSHVQVL	-	453	3.7	7.31	7.92E-15	-	aberrant splicing	374
patient2	YAISVKRTI	YAISVKGTI	453.15	1	1.39	2.08E-13	0.828336928	SNV	375
patient2	RSFDYEQFYEL	RSFDYEQFHEL	493.24	1.2	4.47	4.26E-13	0.685084101	SNV	376
patient3	RLFPYALHK	RLLPYALHK	3.56	15.9	42.15	9.09E-07	0.544342508	SNV	1
patient3	KMFCAGYPK	KMFCAGYPE	5.42	1.1	16.71	7.01E-09	0.001370168	SNV	2
patient3	FSDVGEVPY	LSDVGEVPY	5.98	1.4	1.06	0.999999849	0.460354119	SNV	3
patient3	FTNESYLELY	FTDESYLELY	6.94	38.3	2.83	6.98E-09	2.776	SNV	4
patient3	RIYDKILQSK	RIYDEILQSK	7.18	1	16.02	5.90E-05	0.533432392	SNV	5
patient3	RVDFSATWYK	RVDFPATWYK	7.92	11.3	3.66	0.992380229	0.782608696	SNV	6
patient3	VIYTGEKLYK	VIHTGEKLYK	8.05	1.4	2.78	1.39E-08	0.171276596	SNV	7
patient3	KTMPFFFIGK	-	8.77	18.8	11.96	2.71E-11	-	aberrant splicing	8
patient3	ILFRTPSVAK	ILSRTPSVAK	9.44	7.7	3.8	4.60E-07	0.521546961	SNV	9
patient3	RTKYDFTWFK	RTKNDFTWFK	10.09	33.7	5.24	0.029853429	0.534427966	SNV	10
patient3	KIYTGEKPYK	KIHTGEKPYK	10.96	6.3	6.71	0.015184997	0.162490734	SNV	11
patient3	RIYGFSFPDPK	RIYGISFPDPK	11.63	4.5	11.13	0.999999997	0.660045403	SNV	12
patient3	AINRPTVLK	AIYRPTVLK	11.64	2.4	5.14	4.56E-07	1.979591837	SNV	13
patient3	HLFDIQGLPK	HLLDIQGLPK	12.1	6.2	3.6	1.74E-08	0.382669197	SNV	14
patient3	KVLWWLLVPK	-	12.11	3.9	6.21	1	-	aberrant splicing	15
patient3	LSDGPSMGRY	LSDSPSMGRY	12.14	27.3	2.41	4.60E-07	1.37020316	SNV	16
patient3	LLFKAGEMRK	LLFEAGEMRK	12.88	1.8	2.93	6.95E-09	0.617153809	SNV	17
patient3	KTYACQQCGK	KPYACQQCGK	12.95	2	25.57	5.94E-05	0.003871994	SNV	18
patient3	GMFRGCGIRK	GMFRGCRIRK	13.41	1.2	3.06	4.56E-07	0.985304923	SNV	19
patient3	CVFASGQLLK	RVFASGQLLK	13.7	1.2	3.02	0.999999774	2.486388385	SNV	20
patient3	ATRYNYTSEK	ATRYNYTSEE	13.98	2.6	1.89	2.15E-10	0.000880142	SNV	21
patient3	RLSPVLAESK	RLSPFLAESK	14.09	11.4	8.53	8.17E-11	1.380019589	SNV	22
patient3	KLRDEISLAK	KLRDEINLAK	14.42	13.9	13.5	9.05E-07	0.753789859	SNV	23
patient3	LVYQFKDMLK	LVYQFKDMPK	14.44	4.1	2.12	0.999941048	0.766861391	SNV	24
patient3	RSLSNFPQLK	RSLSNSPQLK	14.89	4.5	3.15	5.99E-05	0.434110787	SNV	25
patient3	ALYKVEINK	ALYKVEINE	15.68	1.4	9.59	2.08E-13	0.001064218	SNV	26
patient3	ATDAVKLAIY	ATDAVKPAIY	15.89	2.2	5.97	0.999999849	0.641501817	SNV	27
patient3	AVNACSLFQK	-	16.26	31.3	9.11	0.999941048	-	aberrant splicing	28
patient3	MTKMNSPMRK	MTKMNSPMGK	16.38	2.6	1.37	2.69E-11	1.121917808	SNV	29
patient3	KLYKCKVCDK	KPYKCKVCDK	17.13	1	11.54	0.000118365	0.002565885	SNV	30
patient3	SLSNFPQLK	SLSNSPQLK	17.63	4.5	3.48	5.94E-05	0.402052452	SNV	31
patient3	KIYTEENPYK	KIHTEENPYK	17.85	4.6	5.44	1	0.18904893	SNV	32
patient3	RVNGGYSGLK	RVNGGYSGLR	18	6.6	8.3	5.90E-05	0.062443627	SNV	33
patient3	ESEPYTFSY	ESEPYTFSH	18.15	1.7	1.82	9.09E-07	0.004566647	SNV	34
patient3	KLSDFWQQLK	KLSDFWQQSK	18.21	1.8	11.22	0.500014857	0.618126273	SNV	35
patient3	TLNPVFGRMYK	TLNPVFGRMYE	18.25	6.6	3.74	5.90E-05	0.001279858	SNV	36
patient3	KMFTYICNHIK	EMFTYICNHIK	19.09	4.1	3.82	0.999999774	0.010401796	SNV	37
patient3	RTVRFFPGSK	RTVRSFPGSK	19.77	13.6	3.49	0.007620663	0.918680297	SNV	38
patient3	FTQEDPQMVY	LTQEDPQMVY	20.14	7.7	1.11	0.000176837	0.417149959	SNV	39
patient3	RTREFTAKK	RTRESTAKK	20.38	102.5	12.4	3.50E-09	0.578156028	SNV	40
patient3	ALLPPPPLAK	ALPPPPPLAK	20.74	3.1	5.11	0.999999547	0.178148085	SNV	41
patient3	MIDNETLLVEY	MIDNETLPVEY	22.28	10.6	3.27	3.53E-09	0.388627246	SNV	42
patient3	YMNPYQLNAK	-	22.42	5.2	1.22	4.56E-07	-	indel	43
patient3	ATNYIYVLNK	ATNYIYVLNE	22.47	7.9	5.13	4.53E-07	0.001418066	SNV	44

Table S3 (continued): Ranked candidate neoantigens of the TESLA patients

patientID	MT_pep	WT_pep	BindAff	Quantification	BindStab	Foreignness	Agretopicity	AlterationType	Rank
patient3	AMTKMNSPMRK	AMTKMNSPMGK	22.58	2.6	3.07	2.69E-11	0.982166159	SNV	45
patient3	KTHSGAKSYK	KTHSGAKSYE	23.61	1.3	10.68	1.06E-08	0.001883394	SNV	46
patient3	LLRLTSEFFK	LLRLTSEFFE	24.14	4.4	1.06	1.60E-10	0.001774157	SNV	47
patient3	KLKPKGSLLPK	KLKPKGSLPPK	24.99	3.8	13.41	0.500014968	0.605231291	SNV	48
patient3	KLNCMAMGISK	KLNCMAMGIPK	25.12	8.1	27.07	5.90E-05	1.075342466	SNV	49
patient3	RLTLPALQQK	RLTLPALQQR	27.86	14.4	5.79	4.12E-13	0.054856558	SNV	50
patient3	ILYRCELEEK	ILYRCESEEK	29.37	1.2	2.79	1.05E-08	0.538109198	SNV	51
patient3	KMTSGVSQGK	KMTSGVSQGE	29.6	4.6	3.5	5.94E-05	0.001549534	SNV	52
patient3	AVCPMSMLNK	-	29.74	31.3	10.71	0.500000226	-	aberrant splicing	53
patient3	TINKCVPVFK	TINKCVPVSK	30.7	1.3	1.41	5.99E-05	0.344286195	SNV	54
patient3	VLKALKSGYK	VLKALKSGYE	30.97	1.2	1.48	7.11E-09	0.00200264	SNV	55
patient3	SVRNITGALFK	SVRDITGALFK	33.35	1.3	2.74	2.67E-11	0.739795918	SNV	56
patient3	MAMEHYPMTK	MAMEHYPMTE	33.53	1.1	1.2	0.909090917	0.00171942	SNV	57
patient3	YLYCYGYWKK	YPYCYGYWKK	33.54	8	1.6	0.999941939	0.006967758	SNV	58
patient3	VTLAFPTAK	VTSAFPTAK	33.64	1.7	3.59	0.007621109	0.82329907	SNV	59
patient3	SVSAMLVLVK	PVSAMLVLVK	33.71	10.7	1.51	3.48E-09	0.026209191	SNV	60
patient3	RLVYQFKDMLK	RLVYQFKDMPK	33.82	4.1	4.07	0.999941048	0.741829349	SNV	61
patient3	YLYCYGYWK	YPYCYGYWK	33.83	8	1.7	0.999941939	0.007333686	SNV	62
patient3	KLAESGSSMGK	KLAESGSSLGK	34.02	3.2	18.91	3.53E-09	0.837931034	SNV	63
patient3	KLSCYLPQEK	ELSCYLPQEK	34.66	6.6	8.84	4.60E-07	0.010500421	SNV	64
patient3	YMYISPPEALK	YMYISPPEALE	34.75	37	1.03	0.015182366	0.001798427	SNV	65
patient3	NLFGLGIFK	DLFGLGIFK	34.91	6.6	1.31	5.94E-05	0.068643451	SNV	66
patient3	GIKAHPVLK	GIEAHPVLK	35.07	3.8	2.31	1.37E-06	0.191702197	SNV	67
patient3	RLARLLASAQK	RLAHLLASAQK	35.07	13	16.32	3.48E-09	1.281329923	SNV	68
patient3	SVMAVTELK	SVMAVTEPK	36.4	1.1	5.78	5.36E-11	0.909772557	SNV	69
patient3	FSDCEIFY	FSDCEISY	37.48	2.9	2.9	0.992380229	0.426782054	SNV	70
patient3	ISEYVSVRFY	IPEYVSVRFY	37.88	12.9	1.59	0.5	0.006477816	SNV	71
patient3	RSNMFIPGEK	RSNMFIPGEE	38.2	1	2.13	0.500000114	0.002279847	SNV	72
patient3	SLAINRPTVLK	SLAIYRPTVLK	39.07	2.4	1.69	0.500000114	2.674195756	SNV	73
patient3	ALLPRNYEK	ALLPRNHEK	39.11	7.7	3.34	3.50E-09	0.94973288	SNV	74
patient3	KAYEKPSEK	KAYEKPPEK	39.15	168.3	3.95	4.53E-07	0.741196516	SNV	75
patient3	MLHMPNICKK	MLHMPNICKR	39.8	3	1.8	7.06E-09	0.056870954	SNV	76
patient3	GTEVTFFLAY	GTEVTFSLAY	40.71	5.8	1.33	8.01E-11	1.446695096	SNV	77
patient3	RSCHCFLHK	RSCHRFLHK	41	3	18.37	8.07E-11	0.620460048	SNV	78
patient3	AMEHYPMTK	AMEHYPMTE	42.2	1.1	1.81	0.909090917	0.001863888	SNV	79
patient3	CSDSGKSFINY	CSDSGKSFINH	42.79	3.5	2.24	0.666666668	0.004584089	SNV	80
patient3	RLPWQICSK	RPPWQICSK	43.01	1.6	1.14	0.015124309	0.004469872	SNV	81
patient3	RVLFSTQFPR	RVLFSTQPPR	43.34	1	2.4	3.48E-09	0.702431118	SNV	82
patient3	ATYKGVPYEVK	ATYKGVPFEVK	43.35	5.2	4.48	0.000120634	0.869085806	SNV	83
patient3	RLKQEKAFSLK	RPKQEKAFSLK	44.37	30.9	9.74	3.53E-09	0.015273876	SNV	84
patient3	VLFQRILQFTK	VLFQRILQCTK	48.2	3.2	4.94	3.48E-09	0.924434216	SNV	85
patient3	STDAFRPEVRY	STDAFRPGVRY	49.06	8.7	2.53	0.015124305	1.286988458	SNV	86
patient3	MSRGGNIFYK	MSRGGNIFHK	49.29	2.6	1.09	0.999999997	0.344902386	SNV	87
patient3	VLIPVTQKK	VLIPVTQRK	51.96	2.3	3.84	3.56E-09	1.275718144	SNV	88
patient3	ILSHNQSPK	ISSHNQSPK	53.66	16.2	3.97	5.90E-05	0.182102012	SNV	89
patient3	RLFIQSLK	RLFIQSLE	55.57	8.2	12.07	3.17E-15	0.004341172	SNV	90
patient3	RLILDESIIPK	RIILDESIIPK	55.6	2.1	2.67	0.501926856	0.633762681	SNV	91
patient3	FTWKSTNFK	LTWKSTNFK	56.41	1.7	1.52	0.007677839	1.798788265	SNV	92
patient3	KVYDIAFSR	EVYDIAFSR	57.42	41.6	3.1	0.999970859	0.016170002	SNV	93
patient3	KIQLVRIEK	EIQLVRIEK	61.52	1.1	1.91	4.53E-07	0.015864338	SNV	94
patient3	VLFSTQFPR	VLFSTQPPR	61.57	1	1.43	3.48E-09	0.491380686	SNV	95
patient3	YLWAAQAEK	YLWAAQAEE	63.76	1.3	1.78	5.90E-05	0.003119935	SNV	96
patient3	SLYLHTTER	SLYLHTMER	64.15	9.4	1.25	5.36E-11	2.701052632	SNV	97
patient3	ASVMAVTELK	ASVMAVTEPK	65.36	1.1	1.19	3.53E-09	1.050128535	SNV	98
patient3	RLCILDGGINK	RICILDGGINK	65.82	3	4.14	0.500000114	0.453305785	SNV	99
patient3	GIYDLPHLRSK	GIYDLPHLRNK	66.12	8.7	2.79	0.007621115	0.950682962	SNV	100
patient3	LMSRGGNIFYK	LMSRGGNIFHK	66.27	2.6	1.1	1	0.239077889	SNV	101
patient3	VSFAGTTSDK	VSSAGTTSDK	68.46	2.4	1.63	8.01E-11	0.118740786	SNV	102
patient3	KLVHVLSQSIK	KLEHVLSQSIK	68.72	4.1	5.87	3.50E-09	0.311726015	SNV	103
patient3	ALNQGIAAVK	ALNRGIAAVK	69	5	6.66	2.73E-11	1.831210191	SNV	104

Table S3 (continued): Ranked candidate neoantigens of the TESLA patients

patientID	MT_pep	WT_pep	BindAff	Quantification	BindStab	Foreignness	Agretopicity	AlterationType	Rank
patient3	KLHVASLSFR	KPHVASLSFR	69.83	36.3	2.97	4.60E-07	0.00738462	SNV	105
patient3	STRRCYLEAK	STRRCHLEAK	71.01	4.1	1.5	0.007619771	0.830332086	SNV	106
patient3	KVEMMKAAYRK	EVEMMKAAYRK	73.85	7.6	1.33	6.95E-09	0.040533049	SNV	107
patient3	KIGHTGEKLYK	KIGHTGEKPYK	74.76	1	3.87	0.000117919	0.454744526	SNV	108
patient3	RVDFSATWY	RVDFPATWY	77.28	11.3	2.74	0.992380229	0.487202118	SNV	109
patient3	SVLIPVTQKK	SVLIPVTQRK	78.38	2.3	1.12	3.58E-09	1.213124903	SNV	110
patient3	SLVPEIARIYK	PLVPEIARIYK	80.87	1.1	1.74	0.007620663	0.025671793	SNV	111
patient3	LLFILILLAK	LPFILILLAK	80.91	3.7	1.03	0.007619785	0.01802687	SNV	112
patient3	IVCKISFNK	TVCKISFNK	81.12	1.3	3.4	4.60E-07	0.710146196	SNV	113
patient3	QLKMKKLSAK	QLKMKKPSAK	83.11	2.5	1.23	4.53E-07	0.595514474	SNV	114
patient3	QTINRMWRK	QTKNRMWRK	83.79	2.2	1.13	0.007620224	0.186386386	SNV	115
patient3	KLGSEPSIK	KLGSGPSIK	85.53	81.2	2.27	1.81E-06	2.352310231	SNV	116
patient3	LSALLLQCLY	LPALLLQCLY	88.24	1.5	1.63	0.998466717	0.029974964	SNV	117
patient3	ALNQFQCSITK	APNQFQCSITK	88.37	20.3	14.66	0.007619771	0.088370884	SNV	118
patient3	RTRKRRKMYK	RTRKRRKMYE	90.87	1.3	2.63	4.53E-07	0.009038985	SNV	119
patient3	ALKCRVALSLK	ALKCRVALSPK	90.89	6.5	4.49	2.73E-11	0.818902604	SNV	120
patient3	RQMEMTPEK	RQVEMTPEK	91.14	7.5	15.77	9.12E-07	0.199435436	SNV	121
patient3	RICASLVLILK	RIRASLVLILK	92.59	2.3	14.13	5.90E-05	3.044722131	SNV	122
patient3	RATRYNYTSEK	RATRYNYTSEE	92.73	2.6	2.31	1.36E-06	0.003297812	SNV	123
patient3	NTYDIYRLEK	NTYDIHRLEK	94.33	1.9	1.26	3.48E-09	1.245773904	SNV	124
patient3	KSYKCKECGK	KSYECKECGK	96.86	1.3	3.77	4.84E-07	0.839050589	SNV	125
patient3	SIYQPKFKTEK	PIYQPKFKTEK	100.22	13.9	3.45	0.992494608	0.021966124	SNV	126
patient3	KTLERETASSK	KTLERETASPK	102.97	2.8	4.33	5.90E-05	1.272805933	SNV	127
patient3	IQKYFWVASK	IQKYFRVASK	103.14	10.6	1.16	0.999941048	1.09385937	SNV	128
patient3	KAYEKPSEKK	KAYEKPPEKK	103.24	168.3	3.42	5.94E-05	0.6370087	SNV	129
patient3	KSFAYNSALR	KSFTYNSALR	106.82	1.6	1.32	4.53E-07	0.678050019	SNV	130
patient3	FLYGADGGAK	FSYGADGGAK	110.22	2.3	1.25	0.007621565	0.327149684	SNV	131
patient3	KAYHEQLSVAK	KAYHEQLSVAE	110.88	2.4	6.51	5.42E-11	0.003734098	SNV	132
patient3	ATVRFLEAEK	ATVRSLEAEK	112.56	6.9	2.01	0.666666717	0.830701107	SNV	133
patient3	GTVNPTGKK	ETVNPTGKK	112.75	3.6	1.7	1.04E-08	0.041268017	SNV	134
patient3	KLWHLDPDTK	KLWHLDPDTE	113.98	2.7	1.1	0.999999849	0.004675245	SNV	135
patient3	KVFPVFRK	KVFPVSRK	118.71	15.6	12.24	0.500000114	0.887087132	SNV	136
patient3	FSDSQSHMHF	FSDSQSHMHS	119.1	1.5	1.3	4.56E-07	0.067565651	SNV	137
patient3	IVDQITLLK	IADQITLLK	119.83	2.2	2.25	2.67E-11	0.131393985	SNV	138
patient3	LVIYTGEKLYK	LVIHTGEKLYK	120.17	1.4	1.11	1.39E-08	0.697932396	SNV	139
patient3	RIATKQASPEK	RIATKQASPEE	122.76	8.1	6.5	0.007620227	0.00440727	SNV	140
patient3	RTREFTAKKIK	RTRESTAKKIK	122.92	102.5	2.35	3.50E-09	0.635836954	SNV	141
patient3	SAEVEMTFY	SAEVEMTSY	124.3	320.8	1.03	2.67E-11	0.213996729	SNV	142
patient3	RTSSLVSGHCK	RTSPLVSGHCK	126.31	6.6	11.32	3.50E-09	1.038135941	SNV	143
patient3	SVAHCARALRK	-	128.7	1.7	2.49	0.000117901	-	indel	144
patient3	RMKGKQEGK	RMKGKQEGE	130.22	1.6	1.6	8.55E-17	0.005608367	SNV	145
patient3	RISLEGLEK	RISPEGLEK	133.3	2.3	4.07	2.75E-11	1.762295082	SNV	146
patient3	STQQFLAEK	STQQSLAEK	136.01	1.9	2.08	0.007619771	0.743711724	SNV	147
patient3	RLLGRGVAR	RLSGRGVAR	143.63	9	1.92	4.53E-07	0.591751813	SNV	148
patient3	CSNHGVCIY	CSNHGVCIH	144.13	2	1.37	0.015238661	0.010992908	SNV	149
patient3	VSCAAVLPGK	VPCAAVLPGK	144.45	1.1	1.44	0.500000113	0.027074493	SNV	150
patient3	KLSDGPSMGR	KLSDSPSMGR	145.06	27.3	1.84	2.79E-11	1.617348645	SNV	151
patient3	KMKKLSAKQQK	KMKKPSAKQQK	145.18	2.5	2.47	5.42E-11	2.20136467	SNV	152
patient3	VLKGDWRCPK	VPKGDWRCPK	147.08	11.3	1.9	0.999999997	0.01025641	SNV	153
patient3	VLSSHGERK	VLSSHGERE	148.03	1.8	2.73	4.53E-07	0.004906374	SNV	154
patient3	ATLFSDSWYY	ATPFSDSWYY	149.69	26	2.2	0.007678285	1.027314529	SNV	155
patient3	NLSAVSTTLK	NLSAISTTLK	150.06	2.5	1.11	0.992380229	1.089918652	SNV	156
patient3	RVGAPRIIDSK	RVGALRIIDSK	151.21	1.8	1.81	1.05E-08	0.471235353	SNV	157
patient3	CLSKPQQSLSK	CLSKPQQSLSE	152.16	1.5	1.47	4.53E-07	0.005239778	SNV	158
patient3	IFYKHSAPK	IFHKHSAPK	153.16	2.6	1.15	5.94E-05	0.167541787	SNV	159
patient3	ESESEPYTFSY	ESESEPYTFSH	154.55	1.7	1.12	0.50000034	0.008894329	SNV	160
patient3	RTSLGSAMLR	RTPLGSAMLR	158.16	5.2	1.89	0.007619771	0.879986647	SNV	161
patient3	ALADGIPLFR	ALADGIPFFR	159.62	10.7	1.42	1.36E-06	0.557702386	SNV	162
patient3	VVLSSHGERK	VVLSSHGERE	160.64	1.8	1.94	5.94E-05	0.00883228	SNV	163
patient3	TTYSSGESYNK	TTYSSGESYDK	161.61	4.7	2.75	0.992380229	0.249263515	SNV	164

Table S3 (continued): Ranked candidate neoantigens of the TESLA patients

patientID	MT_pep	WT_pep	BindAff	Quantification	BindStab	Foreignness	Agretopicity	AlterationType	Rank
patient3	VLLEVPQVK	VPLEVPQVK	165.57	11.4	2.13	3.48E-09	0.00764493	SNV	165
patient3	RAATASPTK	RAATASPTR	168.62	37.7	2.2	4.60E-07	0.067998516	SNV	166
patient3	ILHTLLTLVNK	-	175.28	5.6	1.49	4.53E-07	-	indel	167
patient3	GIFQPIYKSGK	GIFQPSYKSGK	177.35	8.2	1.93	0.007678274	1.272146905	SNV	168
patient3	AQLLSHSPK	AQSLSHSPK	178.76	11.9	2.39	4.56E-07	0.887454699	SNV	169
patient3	ALKNPCYDMK	ALENPCYDMK	180.4	37	1.5	5.94E-05	0.339857953	SNV	170
patient3	GLVLGIYFK	GLVLGIYSK	182.29	5.1	1.13	2.72E-06	0.455702215	SNV	171
patient3	SVHNLCSHK	SVHNLRSHK	185.94	27	2.71	4.53E-07	1.407889755	SNV	172
patient3	ALYCFDYDLEK	ALYCFDYDLEE	187.42	16.2	11.42	0.015181924	0.006419095	SNV	173
patient3	RLFGSMVREER	-	188.38	31.3	2.23	5.34E-11	-	aberrant splicing	174
patient3	TVLKALKSGYK	TVLKALKSGYE	188.97	1.2	1.03	0.007619778	0.006849213	SNV	175
patient3	MTSGVSQGK	MTSGVSQGE	190.72	4.6	1.38	4.53E-07	0.007614947	SNV	176
patient3	ATNQKGSVK	ATNQKGSVE	191.07	17.2	2.51	5.34E-11	0.007493041	SNV	177
patient3	HIHQRVYKK	HIHQRVHKK	194.15	1.9	1.38	0.750000029	0.915413268	SNV	178
patient3	MTFYVLLAY	MTSYVLLAY	197.15	320.8	1.11	5.36E-11	12.80194805	SNV	179
patient3	VLKPATEEK	VLKPATEGK	197.71	1.2	2.56	1.24E-12	1.644568291	SNV	180
patient3	GMLRGIASGIK	GMLRGIASGMK	204.8	1.1	1	1.05E-08	1.195074984	SNV	181
patient3	SLAKQEAQRLK	NLAKQEAQRLK	208.17	13.9	1.32	2.69E-11	0.079138851	SNV	182
patient3	KQRPKRSLRK	-	212.55	7.9	1.99	4.53E-07	-	aberrant splicing	183
patient3	ATKQASPEK	ATKQASPEE	212.9	8.1	2.52	3.53E-09	0.007957097	SNV	184
patient3	LQLAEPFYK	LQLAEPFWK	220.96	2.7	1.18	5.90E-05	0.314283276	SNV	185
patient3	ATGGARGLQK	-	221.23	3.9	1.76	7.01E-09	-	aberrant splicing	186
patient3	KANNSACNK	KADNSACNK	224.44	2.3	2.24	4.56E-07	0.144678657	SNV	187
patient3	AQALYKVEINK	AQALYKVEINE	227.29	1.4	1.38	1.07E-10	0.006938413	SNV	188
patient3	SIVPVSFTIMK	SIVPVPFTIMK	229.48	1.5	1.18	0.992438293	0.862966306	SNV	189
patient3	SLKTNQTLQLK	SPKTNQTLQLK	233.04	6.5	2.22	4.53E-07	0.191435354	SNV	190
patient3	ETSPDSHHY	ETSPDSHHH	235.78	1.4	1.29	0.999999547	0.015924916	SNV	191
patient3	ASVNLSLVK	ASVNPSLVK	238.52	3.5	1.15	5.90E-05	1.627567383	SNV	192
patient3	SLCTKFVSYIK	SLCTKSVSYIK	238.71	5	2.23	0.000117905	0.8515625	SNV	193
patient3	KLVYSILER	KLVYSILEG	240.44	1.3	1.93	5.90E-05	0.015013144	SNV	194
patient3	RFFPGSKEYK	RSFPGSKEYK	247.42	13.6	1.01	0.007794369	8.333445605	SNV	195
patient3	KQQFVNLKEK	-	250.03	6.4	1.31	0.000235772	-	aberrant splicing	196
patient3	LTLPALQQK	LTLPALQQR	251.54	14.4	1.23	4.11E-13	0.053933982	SNV	197
patient3	RTPNSGSSASK	RTPNSGSPASK	256.13	2.2	1.77	1.81E-06	0.71460856	SNV	198
patient3	ASVHNLCSHK	ASVHNLRSHK	257.15	27	1.72	4.53E-07	1.083832083	SNV	199
patient3	DSLERSFLY	DPLERSFLY	268.29	2.3	1.06	0.007735879	0.016386224	SNV	200
patient3	ASTQQFLAEK	ASTQQSLAEK	268.69	1.9	1.78	0.007619771	0.756830601	SNV	201
patient3	RSDGVVSVNK	RRDGVVSVNK	269.38	23.4	1.85	8.20E-13	0.021430901	SNV	202
patient3	QLASVNLSLVK	QLASVNPSLVK	273.19	3.5	1.89	5.94E-05	1.205125943	SNV	203
patient3	KVAASPTCRR	KVATSPTCRR	284.21	7.9	3.28	0.015124299	0.859913467	SNV	204
patient3	LTINKCVPVFK	LTINKCVPVSK	286.83	1.3	2.01	0.500000341	0.411219911	SNV	205
patient3	HLDLHSCPK	QLDLHSCPK	302.34	7.1	1.15	0.007621115	0.525415776	SNV	206
patient3	ASEESAPIQYY	ASEGSAPIQYY	307	2.1	1.52	3.48E-09	0.785487668	SNV	207
patient3	WVDPEDLY	WVDPEDPY	312.37	2.8	1.12	0.500000001	0.231366333	SNV	208
patient3	KAVTHWVIK	RAVTHWVIK	316.24	1.7	1.25	9.09E-07	1.186953421	SNV	209
patient3	HTSCKEFLLTK	HTSCKESLLTK	318	7.1	1.76	5.99E-05	0.748517089	SNV	210
patient3	ALSCHSSLR	AFSCHSSLR	319.79	4.8	1.39	9.44E-07	0.05060649	SNV	211
patient3	SSNDAMVMDK	SSNDAMVMDE	320.1	1.4	1.08	3.65E-17	0.010361319	SNV	212
patient3	KILEADKSKPK	KISEADKSKPK	321	9.1	2.7	0.5	0.721072849	SNV	213
patient3	RVRCRRLAR	RVRCRRLAH	322.95	13	1.5	0.000119263	0.317617207	SNV	214
patient3	YVLKPATEEK	YVLKPATEGK	330.41	1.2	1.11	1.24E-12	1.824461623	SNV	215
patient3	CSNNPISEHFY	CSNNPISEHFH	340.2	5.7	1.09	0.999999551	0.017252326	SNV	216
patient3	RVCSLLTFHR	RVCSLLTSHR	353.45	1	3.34	0.992380675	0.688623921	SNV	217
patient3	KTQLIAHDKK	KTQLIAHDKE	354.33	41.6	1.06	2.11E-13	0.038843881	SNV	218
patient3	TSCKEFLLTK	TSCKESLLTK	361.87	7.1	1.35	5.90E-05	0.589105768	SNV	219
patient3	GVFGDVQLGWK	GVFGDVQLGWE	364.74	1.8	1.06	0.999941051	0.011440089	SNV	220
patient3	KSCVNQTWSVK	-	370.74	9.7	2	0.992380232	-	aberrant splicing	221
patient3	GIFQPIYK	GIFQPSYK	372.36	8.2	4.03	0.000118351	0.898855791	SNV	222
patient3	GSFRSSFLCR	GSFRSPFLCR	389.22	29.9	1.45	0.996175545	1.004464631	SNV	223
patient3	NTGKANQTYY	NTGKADQTYY	397.62	10.6	1.07	1.23E-12	0.910510648	SNV	224

Table S3 (continued): Ranked candidate neoantigens of the TESLA patients

patientID	MT_pep	WT_pep	BindAff	Quantification	BindStab	Foreignness	Agretopicity	AlterationType	Rank
patient3	LLMDNLCDK	LLMDNLRDK	400.19	4.4	1.02	0.500000001	1.677031388	SNV	225
patient3	NTDTCSCSHF	-	409.95	17.3	1.31	5.94E-05	-	aberrant splicing	226
patient3	SIYQPKFK	PIYQPKFK	419.16	13.9	2.77	0.667517631	0.036273887	SNV	227
patient3	ATDAVKLAIYK	ATDAVKPAIYK	423.42	2.2	3.24	1	2.11815908	SNV	228
patient3	VSCRSVWEY	VPCRSVWEY	436.54	13.5	2.99	1.81E-06	0.039510745	SNV	229
patient3	LTMSAAIPMAY	PTMSAAIPMAY	451.74	8.1	2.48	1.36E-06	0.291298572	SNV	230
patient3	RAASNYTEIK	RAASNFTEIK	451.82	7.4	2.2	3.48E-09	1.853620513	SNV	231
patient3	KVAASPTCR	KVATSPTCR	459.3	7.9	4.84	2.75E-11	0.740710876	SNV	232
patient3	AIRNSLEWHCK	AIRNPLEWHCK	463.51	5.6	1.83	0.000176837	1.446615274	SNV	233
patient3	AATDAVKLAIY	AATDAVKPAIY	482.82	2.2	1.08	0.999999849	0.690788909	SNV	234
patient3	VLLEVPQVKPK	VPLEVPQVKPK	487.99	11.4	2.73	0.000117912	0.02204386	SNV	235
patient3	DTDSRFISY	DTDSRFIPY	13.08	17.1	1.21	4.16E-13	0.725860155	SNV	236
patient3	RSFLFFKSGK	RSFLSFKSGK	18.49	2.2	2.52	3.67E-17	1.051165435	SNV	237
patient3	GVSFLILPK	GVSFLVLPK	25.1	11.2	2.07	2.07E-13	0.96390169	SNV	238
patient3	ASFFGVHSK	ASFFGVHPK	28.47	120.5	1.82	4.11E-13	1.380029084	SNV	239
patient3	RLGVSFLILPK	RLGVSFLVLPK	31.19	11.2	6.73	2.14E-13	0.99553144	SNV	240
patient3	ILAEKTAQK	ILAVKTAQK	34.25	12.8	4.44	3.64E-17	1.135986733	SNV	241
patient3	RVMRSTLVLH	RVTRSTLVLH	64.94	4	3.27	2.11E-13	0.138258463	SNV	242
patient3	KMEPGLELGK	KMEPGLEQGK	70.51	17.2	1.02	6.31E-13	0.204828027	SNV	243
patient3	KMNSPMRK	KMNSPMGK	90.9	2.6	10.76	1.59E-15	1.20301747	SNV	244
patient3	AVISMRNGK	AVISMRDGK	93.92	2.5	1.34	1.61E-15	0.278843299	SNV	245
patient3	KILQSKVLPSK	EILQSKVLPSK	100.59	1	4.73	6.23E-13	0.123178467	SNV	246
patient3	GVKISVVSK	GVKISVVTK	131.31	10.7	1.02	2.08E-13	0.82413858	SNV	247
patient3	ATGLSRLTK	ATGLSGLTK	143.6	39.7	1.65	6.38E-15	0.580835659	SNV	248
patient3	SSFSSTLER	SSSSSTLER	239.85	1.8	1.39	2.43E-17	0.12377119	SNV	249
patient3	RLLASAQK	HLLASAQK	291.94	13	17.66	2.07E-13	0.145510913	SNV	250
patient3	VLNKEDLQK	VLNEEDLQK	304.3	7.9	2.4	6.18E-13	0.392331296	SNV	251
patient3	TTKKVEMMK	TTKEVEMMK	324.75	7.6	1.18	7.94E-15	0.605481495	SNV	252
patient3	LSENVLSY	LSGNVLSY	417.03	2.7	1.22	3.21E-15	0.110524223	SNV	253
patient3	KMKKLSAK	KMKKPSAK	468.17	2.5	3.98	2.10E-13	1.294395753	SNV	254
patient1	YVFPAITTPR	YVSPAITTPR	3.31	1.2	10.62	4.53E-07	0.789976134	SNV	1
patient1	VLASLCLYV	VLAPLCLYV	5.15	2.7	21.84	0.992380232	1.23501199	SNV	2
patient1	FLDPDLTNI	SLDPDLTNI	5.28	5.2	5.09	3.50E-09	0.147651007	SNV	3
patient1	FVLASLCLYV	FVLAPLCLYV	5.55	2.7	9.06	0.992380232	1.188436831	SNV	4
patient1	FLGSLLILV	SLGSLLILV	5.78	17.9	8.45	0.999999997	0.558454106	SNV	5
patient1	FVFSKYCHR	FVFSKYRHR	6.51	68.6	9.21	4.60E-07	0.382491187	SNV	6
patient1	LLAPLIATL	LLAPLIATP	8.7	25.2	7.34	0.666666717	0.007504982	SNV	7
patient1	DTIDVSKLNR	DTIDVSNLNR	9.98	3.5	2.43	5.36E-11	1.579113924	SNV	8
patient1	SMLTYPSVLV	SMLTYPFVLV	11.67	31.3	3.37	0.500059062	2.08765653	SNV	9
patient1	ITNFYETAMR	ITNSYETAMR	12.68	7.3	2.97	4.53E-07	0.454969501	SNV	10
patient1	KLLSEFFSCL	KLLSEFSSCL	13.26	5.1	4.16	0.500014852	0.356355818	SNV	11
patient1	SAICYILFEK	-	14.31	178.2	3	0.501927079	-	aberrant splicing	12
patient1	LLSEFFSCL	LLSEFSSCL	14.55	5.1	7.87	0.500000113	0.889364303	SNV	13
patient1	EIIPQCIAR	KIIPQCIAR	15.27	66.7	2.61	9.09E-07	0.029509527	SNV	14
patient1	FLGSLLILVV	SLGSLLILVV	15.94	17.9	5.41	0.999999997	0.514691637	SNV	15
patient1	SLHDLTDGV	SLHGLTDGV	15.96	4.9	2.36	4.53E-07	0.410493827	SNV	16
patient1	FSAVNSLIQR	FPAVNSLIQR	16.01	12.5	1.89	3.48E-09	0.63531746	SNV	17
patient1	YLYHRVDVI	DLYHRVDVI	16.85	11.2	6.46	3.48E-09	0.001835986	SNV	18
patient1	SVLVSNLMAV	FVLVSNLMAV	17.28	31.3	1.74	4.53E-07	2.93877551	SNV	19
patient1	FLSIPMFSWL	-	18.1	18.2	1.22	0.992380239	-	aberrant splicing	20
patient1	FLDPDLTNIL	SLDPDLTNIL	18.27	5.2	1.95	3.50E-09	0.053366438	SNV	21
patient1	ATFEEWFPQR	ATFEEWFPQG	18.99	3.2	6.96	4.53E-07	0.001218305	SNV	22
patient1	SAVNSLIQR	PAVNSLIQR	19.26	12.5	2.19	3.48E-09	0.042782887	SNV	23
patient1	HTVTYTPSQK	HTVTYTPSQE	19.81	11.3	3.99	2.27E-06	0.003326812	SNV	24
patient1	AESASGNQGSW	-	21.43	178.2	1.58	0.999941048	-	aberrant splicing	25
patient1	SLCLYVVVGV	PLCLYVVVGV	22.58	2.7	5.95	0.500014739	0.017304273	SNV	26
patient1	TLANRFSAV	TLANRFPAV	22.82	12.5	2.08	1.36E-06	2.813810111	SNV	27
patient1	LQDSGLWFPV	LRDSGLWFPV	25.36	1.8	3.15	1.43E-08	0.004018107	SNV	28
patient1	CTFCPPPLPK	RTFCPPPLPK	25.43	24.8	1.96	0.007736785	0.122743508	SNV	29
patient1	AETMQSLAAM	-	26.11	14	1.13	3.50E-09	-	indel	30

Table S3 (continued): Ranked candidate neoantigens of the TESLA patients

patientID	MT_pep	WT_pep	BindAff	Quantification	BindStab	Foreignness	Agretopicity	AlterationType	Rank
patient1	LVLPPVMASV	-	26.31	178.2	3.03	0.992380232	-	aberrant splicing	31
patient1	KAWENFPNV	KAWENSPNV	26.53	6.8	2.42	0.007853325	0.39095196	SNV	32
patient1	FSAKAGPAR	FSAKAGPAQ	26.96	14.7	2.42	3.50E-09	0.003204842	SNV	33
patient1	AMKTFGAHSF	AMETFGAHSF	27.55	2.6	15.81	3.48E-09	0.06184757	SNV	34
patient1	MLTYPSVLV	MLTYPFVLV	28.08	31.3	3.03	0.000236224	1.612866169	SNV	35
patient1	LTFEKYTIMK	LTFEEYTIMK	30.88	1.4	3.84	1	0.891197691	SNV	36
patient1	YVFPAITTPRR	YVSPAITTPRR	30.93	1.2	5.43	0.007620217	0.432345541	SNV	37
patient1	ALDHMFMYFL	ALGHMFMYFL	35.51	36.9	5.06	0.015124299	0.431732523	SNV	38
patient1	EENSTGSLY	EGNSTGSLY	36.23	3.5	1.32	7.01E-09	0.002047407	SNV	39
patient1	GLVSIYVSL	GLVSIHVSL	38.23	1.3	1.71	3.50E-09	0.701725404	SNV	40
patient1	FSAHQCMHK	FSAHQRMHK	38.77	2.1	3.36	2.71E-11	1.025118985	SNV	41
patient1	NLFNTYLCL	NLFNTYPCL	40.12	4.9	1.82	0.007630933	0.849819953	SNV	42
patient1	ALKYADNPEV	ALKYADDPEV	40.16	1.7	1.51	0.999999547	0.56191409	SNV	43
patient1	FVANLFNTYL	FVANLFNTYP	44.28	4.9	1.77	1.01E-07	0.008319164	SNV	44
patient1	RLSEVMARM	RLSEAMARM	45.25	20.9	3.27	3.48E-09	0.526040456	SNV	45
patient1	VLLEWNWV	-	47.87	1	3.35	0.999999547	-	aberrant splicing	46
patient1	LSVGHQLFHR	LSVGHQLSHR	49.56	6.9	2.77	0.007620663	0.433444114	SNV	47
patient1	LTFVTIPR	-	49.97	68.6	6.41	1.04E-12	-	aberrant splicing	48
patient1	FSWLGITQSK	-	55.32	18.2	1.01	4.53E-07	-	aberrant splicing	49
patient1	RMPKMGKTIY	RMPKMGKTIH	57.84	109.3	3.24	2.81E-11	0.030896446	SNV	50
patient1	HMVNAMDQSY	HMVNAMDRSY	60.23	6	6.31	2.67E-11	1.308778792	SNV	51
patient1	HLLPLPSYGL	HLPPLPSYGL	62.55	5.8	2.25	0.500030164	0.069663322	SNV	52
patient1	SLIGGTMKLL	-	65.28	141	1.32	0.500014968	-	aberrant splicing	53
patient1	VLTEIFLGSL	VLTEISLGSL	68.5	17.9	1.62	1.44E-12	0.920327825	SNV	54
patient1	FAVLLEALALR	YAVLLEALALR	70.06	7.3	1.4	2.75E-11	1.1260045	SNV	55
patient1	AEQAIIHVSL	AERAIIHVSL	70.69	2.3	1.28	2.71E-11	0.199239008	SNV	56
patient1	KLLSEFFSCLA	KLLSEFSSCLA	70.94	5.1	3.87	0.500014852	0.235454214	SNV	57
patient1	ATFEEWFPQRR	ATFEEWFPQGR	72.68	3.2	2.24	4.53E-07	1.326277372	SNV	58
patient1	SMLTYPSVL	SMLTYPFVL	74.68	31.3	1.04	0.500059062	14.2791587	SNV	59
patient1	SVGHQLFHR	SVGHQLSHR	75.78	6.9	3.93	0.007620217	0.533436576	SNV	60
patient1	FLTPGRAQVLL	-	79.24	1	3.36	0.007678727	-	aberrant splicing	61
patient1	SMIRLSEVM	SMIRLSEAM	79.93	20.9	1.15	2.67E-11	1.205035429	SNV	62
patient1	HLQDSGLWFPV	HLRDSGLWFPV	86.93	1.8	2.95	4.70E-07	0.423491012	SNV	63
patient1	KLLSEFFSC	KLLSEFSSC	90.22	5.1	2.07	1.36E-06	0.262626263	SNV	64
patient1	TQMPGLRSWPL	-	90.34	621.5	1.56	1	-	aberrant splicing	65
patient1	VLIDLIQRTKV	VLIDLIQRTKD	90.99	30.1	4.69	6.98E-09	0.003642399	SNV	66
patient1	FLHEVTVRNRL	FLHEVTVGNRL	95.8	6.4	2.19	8.07E-11	2.503266266	SNV	67
patient1	QEKAGWFQF	QEKAGWFRF	97.7	17.3	1.02	0.992380232	1.0636908	SNV	68
patient1	VLLHAFEGYNV	MLLHAFEGYNV	104.78	32.1	4.04	0.007621558	2.605171556	SNV	69
patient1	FLDPDLTNILA	SLDPDLTNILA	111.29	5.2	1.66	3.50E-09	0.074835924	SNV	70
patient1	FIFHKSTMCF	-	112.96	10.1	2.75	5.90E-05	-	aberrant splicing	71
patient1	RVYDALNLL	RVYDALNVL	116.83	49.8	1.89	0.500000001	0.317602284	SNV	72
patient1	YLYHRVDVIF	DLYHRVDVIF	119.16	11.2	2.92	0.007619781	0.019389103	SNV	73
patient1	HQHFYICKNF	HQHFYICENF	119.4	2.3	2.49	1.05E-08	1.407189157	SNV	74
patient1	SLIGGTMKLLL	-	123.29	141	2.09	0.500015082	-	aberrant splicing	75
patient1	YITDVPNRFHV	YITDVPNGFHV	126.94	9	3.35	2.09E-08	1.050914811	SNV	76
patient1	FQNLTQLQY	FQNLTQLRY	130.69	1.9	11.35	0.800000018	0.718945979	SNV	77
patient1	VLVLPPVMASV	-	131.52	178.2	2.05	0.992380678	-	aberrant splicing	78
patient1	MVNAMDQSY	MVNAMDRSY	135.58	6	4.83	2.67E-11	1.196540464	SNV	79
patient1	HTDQDCLEER	HTDQDCLGER	139.34	2.3	2.25	9.09E-07	1.151570248	SNV	80
patient1	EMASVLFKA	EVASVLFKA	140.66	5.7	1.84	1.23E-12	0.056945293	SNV	81
patient1	LLPLPSYGL	LPPLPSYGL	141.51	5.8	1.08	0.50003016	0.00671468	SNV	82
patient1	FVTIPRTSTF	-	143.62	68.6	1.26	4.53E-07	-	aberrant splicing	83
patient1	SIYVSLLEY	SIHVSLLEY	145.54	1.3	3.4	2.73E-11	0.670660338	SNV	84
patient1	FQNLTQLQYL	FQNLTQLRYL	157.13	1.9	1.77	0.999999999	0.412306481	SNV	85
patient1	FVLASLCLY	FVLAPLCLY	163.12	2.7	1.55	0.500000341	0.598935194	SNV	86
patient1	SLHDLTDGVF	SLHGLTDGVF	165.86	4.9	1.96	5.94E-05	1.061979767	SNV	87
patient1	REMLKRQERF	REMLKRQERL	168.96	16.1	1.55	5.36E-11	0.13510099	SNV	88
patient1	KLTVESSHSL	-	170.19	18.2	1.24	5.90E-05	-	aberrant splicing	89
patient1	HALDKARTRR	HALDKARTGR	172.22	34.5	1.36	8.07E-11	1.843305148	SNV	90

Table S3 (continued): Ranked candidate neoantigens of the TESLA patients

patientID	MT_pep	WT_pep	BindAff	Quantification	BindStab	Foreignness	Agretopicity	AlterationType	Rank
patient1	KTWKAIIVAR	KTWKAIIAAR	174.17	4.3	2.13	0.992380229	1.456879967	SNV	91
patient1	KLSSGTESSF	KLSSETESSF	174.94	2.4	5.39	3.48E-09	0.517497412	SNV	92
patient1	FFVEASMSV	SFVEASMSV	186.21	57.7	1.03	2.69E-11	0.135127682	SNV	93
patient1	QLDSGTLIV	QLDPGTLIV	186.27	21	1.32	5.90E-05	2.001181779	SNV	94
patient1	FLGSLLIL	SLGSLLIL	189.97	17.9	2.66	0.999999997	0.148263482	SNV	95
patient1	VVLKSTFDR	VVLKSTFNR	200.36	39.6	1.46	0.007619771	3.675655843	SNV	96
patient1	SLHDLTDGVFI	SLHGLTDGVFI	201.64	4.9	1.99	0.500000227	0.488267913	SNV	97
patient1	VQNGKLCFMF	VRNGKLCFMF	209.62	2.3	3.26	1.64E-12	0.018529404	SNV	98
patient1	REGVSFSW	REGVSFPW	212.96	3	1.36	1.05E-08	1.125105664	SNV	99
patient1	VTVRNRLIR	VTVGNRLIR	214.9	6.4	1.01	5.90E-05	2.975630019	SNV	100
patient1	LLQNRELVPGL	-	220.71	61.2	1.46	9.09E-07	-	aberrant splicing	101
patient1	IVLKLTHLL	IVLKLTHLP	222.12	5.8	2.47	3.48E-09	0.014220403	SNV	102
patient1	SAAAAPSPF	SAAAAPSPV	222.69	22.2	3.46	5.90E-05	0.044437571	SNV	103
patient1	VSIYVSLLEY	VSIHVSLLEY	229.05	1.3	1.87	5.40E-11	0.719671977	SNV	104
patient1	ALKEIIPQC	ALKKIIPQC	241.75	66.7	1.67	9.09E-07	0.103545164	SNV	105
patient1	KQEKAGWFQF	KQEKAGWFRF	253.69	17.3	1.39	0.992380232	0.607030054	SNV	106
patient1	GQWMGLLDLEV	GQWMGLPDLEV	263.42	6.5	1.93	0.992380233	1.554375406	SNV	107
patient1	VLTEIFLGSLL	VLTEISLGSLL	272.93	17.9	2.22	4.56E-07	1.525259864	SNV	108
patient1	FQVKLGSAADF	SQVKLGSAADF	285.03	11.3	4.19	5.90E-05	1.154061058	SNV	109
patient1	RSLTSIPAF	QSLTSIPAF	285.38	10.6	1.41	5.38E-11	0.194338325	SNV	110
patient1	LTHLLPLPSY	LTHLPPLPSY	298.73	5.8	1.36	6.03E-05	0.856450688	SNV	111
patient1	FLTPGRAQVL	-	327.55	1	1.8	0.007678724	-	aberrant splicing	112
patient1	HQLFHRDTF	HQLSHRDTF	329.43	6.9	2.11	4.53E-07	0.870195737	SNV	113
patient1	RVYDALNLLM	RVYDALNVLM	336.97	49.8	2.39	0.666666667	1.298385543	SNV	114
patient1	SIAHPALPTI	-	347.96	10	1.68	9.23E-07	-	aberrant splicing	115
patient1	YLYHRVDV	DLYHRVDV	365.26	11.2	8.83	3.48E-09	0.01402567	SNV	116
patient1	FAQARFDAY	YAQARFDAY	375.73	19.7	2.05	3.48E-09	1.072197015	SNV	117
patient1	LEALDHMFMY	LEALGHMFMY	375.83	36.9	1.65	0.015124302	1.059720852	SNV	118
patient1	ILPFFYLGSAY	ILPFLYLGSAY	396.37	68.3	1.35	0.999941048	0.408498315	SNV	119
patient1	VEKSELWMY	VEKSELWVY	397.36	9.3	1.11	0.501941593	0.964348987	SNV	120
patient1	RLQLTFEKY	RLQLTFEEY	399.89	1.4	3.75	0.500000001	1.890196635	SNV	121
patient1	VQNGKLCFM	VRNGKLCFM	404.77	2.3	2.98	4.15E-13	0.021928734	SNV	122
patient1	LLSVGHQLF	LLSVGHQLS	416.25	6.9	2.01	9.05E-07	0.018045158	SNV	123
patient1	SAKAGPARPY	SAKAGPAQPY	421.56	14.7	6.43	5.94E-05	1.031516101	SNV	124
patient1	KFMENIMEM	KSMENIMEM	424.85	19.6	1.7	0.000117898	1.364453865	SNV	125
patient1	KTIYKYVHLF	KTIHKYVHLF	425.22	109.3	1.05	5.90E-05	0.64494699	SNV	126
patient1	HIIPRENPF	HIIPGENPF	450.14	2	3.8	0.03702834	0.876014401	SNV	127
patient1	VLLHAFEGY	MLLHAFEGY	496.07	32.1	1.3	3.53E-09	3.077548235	SNV	128
patient1	ESSHSLSTER	-	11.55	14.1	1.55	8.20E-13	-	aberrant splicing	129
patient1	YLFIKDNEI	YLFIEDNEI	15.98	3.2	1.63	6.16E-13	0.891741071	SNV	130
patient1	AVIAVQNGK	AVIAVRNGK	38.29	2.3	1.56	2.08E-13	1.038795442	SNV	131
patient1	LAVHMQNHAR	LAVHMQTHAR	53.16	2.8	1.9	2.22E-13	1.379346134	SNV	132
patient1	MQSLAAMQQF	-	69.36	14	6.55	4.10E-13	-	indel	133
patient1	AVHMQNHAR	AVHMQTHAR	100.98	2.8	1.84	2.21E-13	1.311939717	SNV	134
patient1	HSMVLDASEKR	HPMVLDASEKR	101.18	1.4	1.82	1.79E-15	0.286685745	SNV	135
patient1	MSFQQVQR	MSFQEVQR	170.84	1.3	4.15	2.07E-13	0.568670528	SNV	136
patient1	MQSLAAMQQFY	-	296.47	14	4.04	4.13E-13	-	indel	137
patient1	LEMASVLF	LEVASVLF	334.81	5.7	2.82	6.18E-13	0.121748205	SNV	138
patient1	RQFVQSAKEL	-	413.58	91	2.43	2.06E-13	-	aberrant splicing	139
patient1	RVMMKLNHL	RVMMELNHL	464.7	5	1.38	6.33E-15	0.832930042	SNV	140
patient12	LPRFNTMPF	LPRFSTMPF	3.78	3.2	18.57	4.56E-07	1.044198895	SNV	1
patient12	LVADVLSPV	LVANVLSPV	4.79	3.2	17.4	5.90E-05	0.569560048	SNV	2
patient12	VLVADVLSPV	VLVANVLSPV	5.86	3.2	10.56	5.90E-05	0.584830339	SNV	3
patient12	RPHLPRRQSI	-	6.42	11.9	10.04	9.12E-07	-	indel	4
patient12	RLATLYMLKK	-	6.54	4.3	16.99	9.30E-07	-	aberrant splicing	5
patient12	QMWSGKFSYV	-	7.49	11.2	5.34	0.022516184	-	indel	6
patient12	SPVARPSARSL	-	9.15	6.5	7.22	0.007677836	-	indel	7
patient12	KPHPSGRVWL	-	9.95	2.9	6.17	6.08E-05	-	indel	8
patient12	GPRAPKAPAF	-	10.39	4.5	9.18	0.66667337	-	indel	9
patient12	KSYAECIKQMW	-	10.78	11.2	47.14	0.992380232	-	indel	10

Table S3 (continued): Ranked candidate neoantigens of the TESLA patients

patientID	MT_pep	WT_pep	BindAff	Quantification	BindStab	Foreignness	Agretopicity	AlterationType	Rank
patient12	WQLDALDFLV	GQLDALDFLV	10.91	1.1	3.65	5.94E-05	0.662416515	SNV	11
patient12	LPRFNTMPFI	LPRFSTMPFI	11.65	3.2	1.95	4.56E-07	1.461731493	SNV	12
patient12	RPKQGKRSQPM	-	14.07	6.5	30.32	0.999941051	-	indel	13
patient12	RPSARSLPLH	-	15.1	6.5	1.18	4.56E-07	-	indel	14
patient12	ALYFNSQWK	ALYFNGQWK	15.63	106.4	4.14	5.36E-11	0.755072464	SNV	15
patient12	KLYESFYGPEK	KLDESFYGPEK	17.21	2.1	23.09	2.75E-11	0.124125496	SNV	16
patient12	LSSDNTRCVW	LSSENTRCVW	18.37	1.4	7.74	0.000117898	1.109969789	SNV	17
patient12	VLQGLLTPL	VLQGLLKPL	18.83	2.9	4.41	0.999941048	0.14189902	SNV	18
patient12	VLFDRLSKL	VLFDGLSKL	19.69	28.5	4.02	0.666679819	2.214848144	SNV	19
patient12	RTAFHRAAEHW	RTAFHRAAEHG	21.71	1.1	59.93	0.007619775	0.002167091	SNV	20
patient12	LPRRQSIGGM	-	23.94	11.9	6.04	7.09E-09	-	indel	21
patient12	RPGKDQVGTL	-	24.21	11.9	2.68	0.007619771	-	indel	22
patient12	RPSARSLPLHI	-	27.05	6.5	2.17	4.63E-07	-	indel	23
patient12	YMLKKYPPEL	-	27.57	4.3	5.4	0.00762067	-	aberrant splicing	24
patient12	WQLDALDFL	GQLDALDFL	30.45	1.1	1.44	8.09E-11	0.434627462	SNV	25
patient12	YIYNPSLAL	YIDNPSLAL	31.15	7.3	1.5	0.000119259	0.285047584	SNV	26
patient12	TAFHRAAEHW	TAFHRAAEHG	36.37	1.1	6.3	0.007619775	0.00289456	SNV	27
patient12	SLYELEKEFL	SIYELEKEFL	38.69	2.3	1.48	4.56E-07	0.079453743	SNV	28
patient12	VARPSARSLPL	-	39.65	6.5	3.87	5.94E-05	-	indel	29
patient12	RGRMQTASL	RGRRQTASL	40.24	1.1	1.5	2.73E-11	0.634600221	SNV	30
patient12	TAMNNQGYSW	-	41.38	1.6	7.19	0.999999997	-	indel	31
patient12	IVLQSVFTSV	-	41.8	4.5	4.01	1.37E-06	-	indel	32
patient12	RSLPLHIPYM	-	42.69	6.5	10.08	0.007619785	-	indel	33
patient12	YQSPDLSKV	YLSPDLSKV	43.43	7.6	4.37	9.05E-07	9.010373444	SNV	34
patient12	KTSQRQSREW	KTSQRRSREW	43.56	16.1	16.9	3.53E-09	0.439644732	SNV	35
patient12	RLPGLILYTL	RLPGLILYTF	47	15	5.03	5.94E-05	0.025242489	SNV	36
patient12	SLWATLAGTTL	SLGATLAGTTL	49.32	4.3	2.52	0.999941048	0.080200338	SNV	37
patient12	VPYGVGKAAV	-	51.76	9.1	4.2	0.500014965	-	aberrant splicing	38
patient12	FLNSFQELQA	FLNSFEELQA	53.07	1.9	2.59	2.67E-11	0.759010297	SNV	39
patient12	GLVMEGHLSK	-	53.92	8.8	2.03	0.007619771	-	indel	40
patient12	GVLVADVLSPV	GVLVANVLSPV	54.35	3.2	1.95	5.90E-05	0.332537934	SNV	41
patient12	VLLLQLLLL	VLLLLLLLL	54.45	48	2.12	0.992380229	0.251920052	SNV	42
patient12	VLQGLLTPLFK	VLQGLLKPLFK	55.08	2.9	2.92	0.999941048	0.987982063	SNV	43
patient12	GPSPSSEAL	GPSPSSGAL	55.81	9.2	3.03	5.94E-05	4.925860547	SNV	44
patient12	SLWATLAGT	SLGATLAGT	57.27	4.3	1.51	0.999941048	0.110557711	SNV	45
patient12	ATAMNNQGYSW	-	59.14	1.6	8.46	0.999999997	-	indel	46
patient12	RPGNLFIGGL	RPGKLFIGGL	60.16	36.9	1.75	9.12E-07	1.31010453	SNV	47
patient12	LVNALYFNSQW	LVNALYFNGQW	60.48	106.4	3.08	4.53E-07	0.492107404	SNV	48
patient12	HLSKRASNAFK	-	64.43	8.8	2.47	4.53E-07	-	indel	49
patient12	SLIGGTMKLL	-	65.28	55.9	1.32	0.500014968	-	aberrant splicing	50
patient12	LLAFGVTSA	LLAFGVASA	65.28	4.4	2.22	1.39E-08	2.06190777	SNV	51
patient12	SVLQGLLTPL	SVLQGLLKPL	67.03	2.9	2.25	0.999941048	0.168510232	SNV	52
patient12	GVHAGPRAPK	-	72.05	4.5	3.33	0.999999997	-	indel	53
patient12	GPAPSGMQAV	-	72.99	1.3	3.22	0.999999997	-	aberrant splicing	54
patient12	NALYFNSQW	NALYFNGQW	73.35	106.4	1.62	5.36E-11	0.573405253	SNV	55
patient12	SPAADGIHAV	-	78.73	6.5	2.91	0.992380229	-	indel	56
patient12	RLTLGGICK	RLTLGGIYK	93.3	2.4	9.3	0.996175544	3.845836768	SNV	57
patient12	ALLAFGVTSA	ALLAFGVASA	94.47	4.4	3.09	1.74E-08	1.380333139	SNV	58
patient12	GMFYAVRWGRK	GMFYAVRRGRK	95.47	10.4	1.71	0.50001542	0.704315751	SNV	59
patient12	VIDAAVLLK	VIDAAVLLE	98.59	16.3	1.37	5.34E-11	0.004457389	SNV	60
patient12	VLFDRLSKLAA	VLFDGLSKLAA	99.36	28.5	2.97	0.666679819	2.208490776	SNV	61
patient12	VLFDRLSKLA	VLFDGLSKLA	99.44	28.5	1.85	0.666679819	2.953370953	SNV	62
patient12	AAVLLKAGW	AAVLLEAGW	99.69	16.3	1.74	2.94E-10	1.057382266	SNV	63
patient12	VLVADVLSPVL	VLVANVLSPVL	99.92	3.2	1.21	0.5	0.348931415	SNV	64
patient12	TSQRQSREW	TSQRRSREW	101.95	16.1	2.46	3.50E-09	0.564288482	SNV	65
patient12	QPMAPSRRRF	-	104.43	6.5	3.53	0.992381127	-	indel	66
patient12	RPPGPSLWATL	RPPGPSLGATL	115.47	4.3	3.23	0.999980399	1.212538066	SNV	67
patient12	RSKTSRPSL	RSKTSRPSI	115.84	2.3	1.66	3.48E-09	0.171368552	SNV	68
patient12	SLIGGTMKLLL	-	123.29	55.9	2.09	0.500015082	-	aberrant splicing	69
patient12	LVADVLSPVLV	LVANVLSPVLV	124.38	3.2	2.78	0.5	0.382178522	SNV	70

Table S3 (continued): Ranked candidate neoantigens of the TESLA patients

patientID	MT_pep	WT_pep	BindAff	Quantification	BindStab	Foreignness	Agretopicity	AlterationType	Rank
patient12	VLLKAGWQNLV	VLLEAGWQNLV	128.15	16.3	3.24	0.999999997	5.047262702	SNV	71
patient12	SISAEAISGV	-	131.28	30.6	1.02	4.56E-07	-	gene fusion	72
patient12	RGFGMFYAVRW	RGFGMFYAVRR	132.15	10.4	3.7	0.500000568	0.005223463	SNV	73
patient12	VLVDGCSVNK	-	137.61	889	1.93	8.15E-11	-	aberrant splicing	74
patient12	HPSGRVWL	-	137.99	2.9	2.44	4.63E-07	-	indel	75
patient12	GPVEEVRAL	GRVEEVRAL	156.72	10	2.73	0.000118354	0.006293541	SNV	76
patient12	ALQGVVLDTL	-	162.42	6.5	2.27	4.53E-07	-	indel	77
patient12	RVWLQQAW	-	164.98	2.9	7.32	0.501927417	-	indel	78
patient12	LTLGGICKF	LTLGGIYKF	166.79	2.4	1.42	0.999941497	3.161895735	SNV	79
patient12	GPVEEVRALL	GRVEEVRALL	188.57	10	1.48	0.999941048	0.014161266	SNV	80
patient12	KLNPSQNVV	KLNPSQNVA	192.42	3.7	3	5.94E-05	0.068917844	SNV	81
patient12	SLLGTTIMNNI	ALLGTTIMNNI	193.03	6.5	2.33	0.000118351	0.808604223	SNV	82
patient12	KTSRPSLYEL	KTSRPSIYEL	196.21	2.3	2.74	0.500029477	0.891823099	SNV	83
patient12	VLLKAGWQNL	VLLEAGWQNL	198.66	16.3	1.42	0.999999997	7.239795918	SNV	84
patient12	SSDNTRCVW	SSENTRCVW	199.65	1.4	2.79	0.000117898	0.880640466	SNV	85
patient12	KAAVRTQW	-	204.52	9.1	19.76	0.029797924	-	aberrant splicing	86
patient12	LLTPLFKNTSV	LLKPLFKNTSV	212.29	2.9	2.8	1.86E-12	0.303518579	SNV	87
patient12	APQPPPSPVA	-	224.55	6.5	2.02	1	-	indel	88
patient12	FLNSFQEL	FLNSFEEL	225.01	1.9	7.41	2.67E-11	1.155378691	SNV	89
patient12	RPTYEEGFL	RPTDEEGFL	231.53	16	1.12	3.56E-09	0.279342213	SNV	90
patient12	TPGTRGPI	TPGTRGPR	236.35	153	1.34	0.999999547	0.013776529	SNV	91
patient12	ALQLGLLAA	ALQLGLRAA	237.57	12.5	2.17	0.007619778	0.307661426	SNV	92
patient12	RGYQSPDLSK	RGYLSPDLSK	239.6	7.6	1.3	4.53E-07	1.300760043	SNV	93
patient12	RPHILFPSVT	-	239.95	1.3	1.37	0.666686521	-	aberrant splicing	94
patient12	RTGKGQPCNK	RTGKGQPSNK	240.15	30.3	2.86	4.70E-07	1.380807268	SNV	95
patient12	RTRGRMQTASL	RTRGRRQTASL	244.78	1.1	2.1	9.05E-07	0.997839468	SNV	96
patient12	SPCLGHVL	SPCRGHVL	255.74	7.3	3.67	4.60E-07	2.105895916	SNV	97
patient12	FVHQSCLLQW	FVHQSCLHQW	273.84	12.2	1.3	5.94E-05	1.64745518	SNV	98
patient12	VLFDRLSK	VLFDGLSK	275.01	28.5	6.59	5.94E-05	0.576649682	SNV	99
patient12	SPSSEALDL	SPSSGALDL	289.41	9.2	1.3	0.007677829	4.826717812	SNV	100
patient12	ASNAFKTW	-	290.06	8.8	11.62	0.999999774	-	indel	101
patient12	GLILYTLRM	GLILYTFRM	306.34	15	1.18	4.53E-07	5.060961507	SNV	102
patient12	APHEPDYRPPA	APPEPDYRPPA	324.68	3.7	1.53	1	0.088924433	SNV	103
patient12	VVVREALEK	VAVREALEK	326.11	3.7	2.72	0.666666768	0.105784389	SNV	104
patient12	RQSREWAGK	RRSREWAGK	330.11	16.1	1.57	1.76E-08	0.022791718	SNV	105
patient12	RVLSRYLLLAR	-	339.02	1.3	2.15	3.56E-09	-	aberrant splicing	106
patient12	RMRSKTSRPSL	RMRSKTSRPSI	350.71	2.3	2.59	3.50E-09	0.157513451	SNV	107
patient12	TPLAVGVL	-	350.76	4.5	2.37	3.53E-09	-	indel	108
patient12	GMQESLLGTTI	GMQEALLGTTI	359.92	6.5	1.39	6.08E-05	1.400412435	SNV	109
patient12	KSYAECIKQM	-	365.2	11.2	1.92	5.90E-05	-	indel	110
patient12	SSFSDYVQCF	SSFSDYVQCV	371.89	9.9	1.38	0.007619775	0.075822262	SNV	111
patient12	GPSLWATL	GPSLGATL	371.98	4.3	2.52	0.999980348	1.238777141	SNV	112
patient12	GLLTPLFK	GLLKPLFK	373.27	2.9	3.43	0.5	1.230208951	SNV	113
patient12	YVKEMYCAW	YVEEMYCAW	392.74	28.2	1.44	3.64E-09	0.180556833	SNV	114
patient12	GTLTRPHLPR	-	397.23	11.9	1.15	9.06E-07	-	indel	115
patient12	KLNPSQNVVV	KLNPSQNVAV	403.71	3.7	4.09	5.94E-05	3.321622511	SNV	116
patient12	RAPGAGRGRSW	RAPGAGRGRSR	438.62	5.8	1.72	0.667517782	0.012930885	SNV	117
patient12	RAFKVSVVNAF	-	472.43	11.2	7.31	4.60E-07	-	indel	118
patient12	GSCRSLVW	-	487.37	1.3	4.19	9.05E-07	-	aberrant splicing	119
patient12	APADHGEAVL	-	17.63	6.5	7.11	2.11E-13	-	indel	120
patient12	ILYTLRMIMAK	ILYTFRMIMAK	29.37	15	5.74	6.18E-13	1.409985598	SNV	121
patient12	KLHVSVEAFV	KLHVSVEALV	30.08	55.4	5.86	2.08E-13	0.351977533	SNV	122
patient12	TPRAFKVSVV	-	30.09	11.2	6.28	4.21E-13	-	indel	123
patient12	YTLRMIMAK	YTFRMIMAK	40.73	15	1.95	1.61E-15	2.340804598	SNV	124
patient12	TPLFKNTSV	KPLFKNTSV	61.05	2.9	1.99	4.78E-15	2.474665586	SNV	125
patient12	QLTNSITEL	QLTHSITEL	335.89	1.2	1.64	2.14E-13	0.688779067	SNV	126
patient16	LLAKQLYRV	LLAKELYRV	6.64	1.1	22.88	0.000117898	1.158813264	SNV	1
patient16	RRLLLLLVLL	-	7.61	9.8	2.84	0.99243829	-	indel	2
patient16	RRSMLFARHL	RRSMVFARHL	9.28	9	2.47	4.53E-07	0.839059675	SNV	3
patient16	SLLLLPLSV	SLLLLPVSV	9.81	25.2	2.14	3.58E-09	0.908333333	SNV	4

Table S3 (continued): Ranked candidate neoantigens of the TESLA patients

patientID	MT_pep	WT_pep	BindAff	Quantification	BindStab	Foreignness	Agretopicity	AlterationType	Rank
patient16	RRRLLLLLVL	-	9.95	9.8	1.05	0.992380229	-	indel	5
patient16	YLIIENRYAV	YLIIENRDAV	11.69	11.4	5.42	4.53E-07	0.19274526	SNV	6
patient16	SVLSALFSI	SVLNALFSI	11.93	8.7	2.89	3.48E-09	0.895645646	SNV	7
patient16	KIMLSHLQLK	-	14.27	24.6	12.46	2.69E-11	-	aberrant splicing	8
patient16	FLGSQWTFKL	-	14.53	8.6	3.03	5.94E-05	-	indel	9
patient16	ATYNVIVEALK	-	15.42	467.9	49.7	3.56E-09	-	aberrant splicing	10
patient16	KLPYSYNLCV	TLPYSYNLCV	15.42	34.4	3.38	5.99E-05	0.574087863	SNV	11
patient16	MLFARHLREV	MVFARHLREV	16.83	9	4.87	4.56E-07	0.090009627	SNV	12
patient16	SLSSDFNPLV	SLSSDFDPLV	17.63	1.3	4.59	0.02990977	0.793786583	SNV	13
patient16	AELLAKQLY	AELLAKELY	20.04	1.1	3.16	5.90E-05	0.569641842	SNV	14
patient16	RRRLLLLL	-	23.1	9.8	1.28	3.50E-09	-	indel	15
patient16	RRSMLFARHLR	RRSMVFARHLR	24.06	9	3.97	4.53E-07	0.760910816	SNV	16
patient16	KRRPSGFFLF	KRPPSGFFLF	24.82	6.7	1.3	0.999988245	0.123519459	SNV	17
patient16	KQLYRVFQK	KELYRVFQK	24.83	1.1	6.14	0.500014852	0.01583849	SNV	18
patient16	EEMYLNEAMF	EEMYLNEAGF	25.49	3.3	1.05	2.67E-11	1.089781958	SNV	19
patient16	RMGQTVIAV	RMGQTVRAV	26.24	39.7	6.5	2.07E-13	0.081445155	SNV	20
patient16	SVRCYVPSCLK	SGRCYVPSCLK	27.08	7	4.96	0.992381124	0.043452448	SNV	21
patient16	LLFGPSCAL	LLFGPSSAL	27.19	8.5	2.43	0.000236221	0.564224943	SNV	22
patient16	RLLRPIYDYL	RRLRPIYDYL	27.98	4.6	2.29	0.007620224	0.013661042	SNV	23
patient16	SQYCRPISTK	SQYCRPISTQ	31.77	3	2.34	5.90E-05	0.002664575	SNV	24
patient16	SLSSDFNPL	SLSSDFDPL	33	1.3	1.23	6.21E-05	0.746775289	SNV	25
patient16	SLHPSAPFL	SLYPSAPFL	37.27	7.7	1.88	0.999999547	3.629016553	SNV	26
patient16	QLSSVRCYV	QLSSGRCYV	37.86	7	3.75	0.007619771	0.271106337	SNV	27
patient16	RSMLFARHLR	RSMVFARHLR	38.06	9	4.36	4.53E-07	0.816913501	SNV	28
patient16	RRRLLLLLVLL	-	38.52	9.8	1.05	0.99243829	-	indel	29
patient16	QSFTSLEVRK	-	38.92	3.6	2.24	5.90E-05	-	aberrant splicing	30
patient16	RRSMLFARH	RRSMVFARH	39.36	9	3.97	4.53E-07	0.649612147	SNV	31
patient16	RLQFSDQAEV	RLQFSDQAGV	39.47	2.5	2.26	2.71E-11	1.002794715	SNV	32
patient16	SEGKLPYSY	SEGTLPYSY	43.8	34.4	1.87	0.999941048	1.210613599	SNV	33
patient16	LIIENRYAV	LIIENRDAV	46.42	11.4	4.74	1.10E-14	0.082767228	SNV	34
patient16	AEGGTAWLEWW	AEGGTAWLEWR	46.56	147.1	1.07	1	0.056755571	SNV	35
patient16	FLCNARVKV	FQCNARVKV	48.51	1.8	5.31	5.90E-05	0.0413689	SNV	36
patient16	KIFLCNARV	KIFQCNARV	53.63	1.8	3.69	0.007678274	0.870334307	SNV	37
patient16	ALVDKVSNPK	TLVDKVSNPK	59.17	33.7	6.31	2.73E-11	0.815575465	SNV	38
patient16	RRFLGACQCV	-	59.53	84.4	5.55	2.82E-11	-	aberrant splicing	39
patient16	VVGAAGVGK	VVGAGGVGK	59.83	7.3	3.85	9.05E-07	0.716956261	SNV	40
patient16	LLLPLSVARPL	LLLPVSVARPL	63.97	25.2	3.85	4.60E-07	1.012343725	SNV	41
patient16	TIITQLSSV	TIITQLSSG	64.47	7	1.94	2.05E-13	0.002528461	SNV	42
patient16	SLIGGTMKLL	-	65.28	43.7	1.32	0.500014968	-	aberrant splicing	43
patient16	ITSLIGGTMK	-	66.88	43.7	3.33	4.63E-07	-	aberrant splicing	44
patient16	SRFFLGSQWTF	-	68.72	8.6	1.62	5.94E-05	-	indel	45
patient16	GLKDLLNPI	GLEDLLNPI	71.41	8.7	2.89	6.98E-09	1.333271098	SNV	46
patient16	RRLLLLLV	-	73.54	9.8	2.14	4.53E-07	-	indel	47
patient16	GRMNRRERRF	-	76.17	84.4	1.16	0.996175544	-	aberrant splicing	48
patient16	AEVSVLYTV	AGVSVLYTV	79.99	2.5	1.15	0.015124298	0.003282283	SNV	49
patient16	RRLLLLLVLLI	RRLLLVLLI	82.86	9.8	2.62	0.999941497	10.17936118	indel	50
patient16	GVLPNYSEGK	GVLPNYSEGT	83.94	34.4	5.15	5.94E-05	0.002306939	SNV	51
patient16	HLMNIAIIA	HLMDIAIIA	85.41	3.6	3.37	3.53E-09	2.433333333	SNV	52
patient16	MLPSNYVEA	MLPANYVEA	86.06	127.1	1.8	4.53E-07	1.047850968	SNV	53
patient16	SSQYCRPISTK	SSQYCRPISTQ	87.01	3	1.64	5.90E-05	0.004105434	SNV	54
patient16	FTANPPSQTK	CTANPPSQTK	97.57	11.4	3.79	0.992380229	1.379861406	SNV	55
patient16	RTADWHLYL	RTADWRLYL	98.49	9	1.58	0.500000115	0.290308318	SNV	56
patient16	TVLVDGCSVNK	-	100.04	2737.8	1.61	8.15E-11	-	aberrant splicing	57
patient16	RRQSMTVKRER	-	100.62	4.4	1.36	4.56E-07	-	indel	58
patient16	VLVDGCSVNK	-	102.74	2737.8	2.26	8.15E-11	-	aberrant splicing	59
patient16	KTDTGVHATLK	KTDPGVHATLK	104.16	399.8	26.8	9.12E-07	1.333674776	SNV	60
patient16	VVVGAAGVGK	VVVGAGGVGK	104.17	7.3	4.27	9.05E-07	0.678410941	SNV	61
patient16	YVYKIVSQL	YVYKNVSQL	110.76	1.4	2.6	0.007620666	0.847177604	SNV	62
patient16	AANGPTALVDK	AANGPTTLVDK	116.17	33.7	2.64	4.53E-07	0.782289562	SNV	63
patient16	YLIIENRYA	YLIIENRDA	116.86	11.4	1.89	4.53E-07	0.064117899	SNV	64

Table S3 (continued): Ranked candidate neoantigens of the TESLA patients

patientID	MT_pep	WT_pep	BindAff	Quantification	BindStab	Foreignness	Agretopicity	AlterationType	Rank
patient16	YAVQDFLHEI	DAVQDFLHEI	123.12	11.4	1.43	4.53E-07	0.020570947	SNV	65
patient16	SLIGGTMKLLL	-	123.29	43.7	2.09	0.500015082	-	aberrant splicing	66
patient16	VRCYVPSCLK	GRCYVPSCLK	123.82	7	1.99	0.992381124	1.993880837	SNV	67
patient16	YVYKIVSQL	YVYKNVSQL	125.74	1.4	1.2	0.007620666	2.066392769	SNV	68
patient16	KRHFTQVEFYL	KRHFTQVEFYQ	126.22	1.5	2.37	3.61E-09	0.085080854	SNV	69
patient16	KSRQNHLQLK	-	142.2	11.7	5.09	9.16E-07	-	indel	70
patient16	SVSDSHVYKYR	SVSDRHVYKYR	148.98	1.3	1.59	2.69E-11	0.325910046	SNV	71
patient16	NLINEAMDV	DLINEAMDV	157.83	2.1	1.37	2.69E-11	0.025596406	SNV	72
patient16	RRNSKGVCEAK	RRNSKGVCEAT	166.7	12.2	4.98	5.90E-05	0.061988696	SNV	73
patient16	EAMFNFVRK	EAGFNFVRK	186.26	3.3	1.22	5.34E-11	0.129783439	SNV	74
patient16	KQLYRVFQK	KELYRVFQK	188.02	1.1	2.71	0.500014852	0.061446653	SNV	75
patient16	SLLAARQLL	SLRAARQLL	191.35	13.7	1.36	2.48E-17	0.033396455	SNV	76
patient16	RLLRPIYVEM	RRLRPIYVEM	198.94	2.5	1.38	4.56E-07	0.030609121	SNV	77
patient16	KRCPGRFVY	KGCPGRFVY	204.1	4.3	2.34	3.61E-09	0.008685225	SNV	78
patient16	YLYVYKIVSQL	YLYVYKNVSQL	212.96	1.4	4.55	0.007620666	1.298694963	SNV	79
patient16	SRQNHLQLKR	-	214.39	11.7	1.14	1.36E-06	-	indel	80
patient16	RRQSMTVK	-	219.59	4.4	5.93	2.67E-11	-	indel	81
patient16	HRAFGRMNRR	-	222.14	84.4	1.05	1.05E-08	-	aberrant splicing	82
patient16	KLLSFHSV	KLLSFHSL	225.3	1.9	20.01	0.007620217	0.3255404	SNV	83
patient16	REYLYVYKI	REYLYVYKN	231.12	1.4	1.24	3.50E-09	0.084327283	SNV	84
patient16	GMLPSNYVEA	GMLPANYVEA	245.57	127.1	1.18	9.09E-07	1.399897389	SNV	85
patient16	SLHPSAPFLA	SLYPSAPFLA	248.19	7.7	1.06	0.999999547	10.57477631	SNV	86
patient16	LLLLPLSVA	LLLLPVSVA	251.36	25.2	1.77	7.06E-09	0.505958132	SNV	87
patient16	VSDSHVYKYR	VSDRHVYKYR	263.9	1.3	1.06	2.67E-11	0.569793803	SNV	88
patient16	AMFNFVRK	AGFNFVRK	276.82	3.3	3.81	2.67E-11	0.066163307	SNV	89
patient16	GLKDLLNPIGV	GLEDLLNPIGV	303.05	8.7	1.41	4.67E-07	1.424040224	SNV	90
patient16	SLAANGPTALV	SLAANGPTTLV	306.24	33.7	4.49	4.56E-07	0.555557571	SNV	91
patient16	RISASCATR	RISASSATR	314.86	2.2	2.52	3.50E-09	2.125278434	SNV	92
patient16	AMFNFVRKC	AGFNFVRKC	356.05	3.3	1.79	2.69E-11	0.016048648	SNV	93
patient16	LLLLPLSV	LLLLPVSV	381.02	25.2	3.25	3.58E-09	0.887744641	SNV	94
patient16	HLYLFQAPTAK	RLYLFQAPTAK	408.21	9	2.42	0.007620227	3.385668077	SNV	95
patient16	ARVKWRPQCR	ARVKWRPECR	408.81	5.4	1.61	5.90E-05	0.891955578	SNV	96
patient16	LSSDFNPLV	LSSDFDPLV	414.1	1.3	1.1	0.029854707	0.456907681	SNV	97
patient16	TRISASCATR	TRISASSATR	433.99	2.2	1.46	3.50E-09	0.648423726	SNV	98
patient16	AIAITNKEKK	-	441.27	11.7	1.58	5.90E-05	-	indel	99
patient16	QTFENALEH	PTFENALEH	449.37	4.1	1.45	3.50E-09	0.056688819	SNV	100
patient16	IENRYAVQDF	IENRDAVQDF	452.48	11.4	1.09	6.95E-09	0.63174355	SNV	101
patient16	SLAANGPTA	SLAANGPTT	481.27	33.7	1.36	3.50E-09	0.191294428	SNV	102
patient16	GLSKYRPAI	GLSKYRPAS	494.42	19.9	1.79	9.09E-07	0.037933244	SNV	103
patient16	YLNEAMFNFV	YLNEAGFNFV	2.81	3.3	16.73	2.14E-13	0.704260652	SNV	104
patient16	SVSDSHVYK	SVSDRHVYK	4.31	1.3	19.94	2.05E-13	0.83044316	SNV	105
patient16	LSVSDSHVYK	LSVSDRHVYK	13.37	1.3	2.65	6.15E-13	0.493904692	SNV	106
patient16	YLNEAMFNF	YLNEAGFNF	43.41	3.3	2.51	2.13E-13	0.376724811	SNV	107
patient16	RRSMLFAR	RRSMVFAR	49.7	9	3.93	8.20E-13	1.016567805	SNV	108
patient16	KRLSRFFL	-	57.02	8.6	5.07	4.18E-13	-	indel	109
patient16	LQFSDQAEV	LQFSDQAGV	73.95	2.5	1.92	6.21E-13	0.979340485	SNV	110
patient16	CVSRNELLQK	SVSRNELLQK	105.04	3.4	4.47	4.72E-15	4.689285714	SNV	111
patient16	KALSNAQAK	QALSNAQAK	137.88	1	3.7	2.05E-13	0.244654613	SNV	112
patient16	ELNPEQRLFK	-	447.76	7.3	1.45	6.20E-13	-	indel	113

Table S4 : Descriptions of the TESLA data

Patient ID	Tumor type[a]	Gender	Status	Cell type	Organ	Treatment (check point inhibitor)	Days on treatment at time of biopsy	Response to treatment
patient1	MM	Male	Metastatic	Epithelial	Skin	Pembrolizumab	-28	Partial response
patient2	MM	Male	Metastatic	Epithelial	Skin	Ipi + Nivo	-25	Partial response
patient3	MM	Male	Metastatic	Epithelial	Skin	Nivolimumab	-11	Complete response
patient12	NSCLC	Male	Primary	Epithelial	Lung	N/A	N/A	N/A
patient16	NSCLC	Female	Primary	Epithelial	Lung	N/A	N/A	N/A
~~patient10[b]~~	~~NSCLC~~	~~Male~~	~~Primary~~	~~Epithelial~~	~~Lung~~	~~N/A~~	~~N/A~~	~~N/A~~

[a] MM: Melanoma; NSCLC: Non-small cell lung cancer;
[b] Data for patient10 is unavailable, thus the results in this paper do not include this patient;
N/A represents that the corresponding content is Not Applicable;

Reference

[1] Chi Zhou, Zhiting Wei, Zhanbing Zhang, Biyu Zhang, Chenyu Zhu, Ke Chen, Guohui Chuai, Sheng Qu, Lu Xie, Yong Gao, and Qi Liu. PTuneos: prioritizing tumor neoantigens from next-generation sequencing data. *Genome Medicine*, 11(1):1–17, 2019.

[2] Jasreet Hundal, Beatriz M. Carreno, Allegra A. Petti, Gerald P. Linette, Obi L. Griffith, Elaine R. Mardis, and Malachi Griffith. pVAC-Seq: a genome-guided in silico approach to identifying tumor neoantigens. *Genome Medicine*, 8(1):1–11, 2016.

[3] Sangwoo Kim, H. S. Kim, E. Kim, M. G. Lee, E. C. Shin, S. Paik, and Sangwoo Kim. Neopepsee: accurate genome-level prediction of neoantigens by harnessing sequence and amino acid immunogenicity information. *Annals of Oncology*, 29(4):1030–1036, 2018.

[4] Fei Duan, Jorge Duitama, Sahar Al Seesi, Cory M. Ayres, Steven A. Corcelli, Arpita P. Pawashe, Tatiana Blanchard, David McMahon, John Sidney, Alessandro Sette, Brian M. Baker, Ion I. Mandoiu, and Pramod K. Srivastava. Genomic and bioinformatic profiling of mutational neoepitopes reveals new rules to predict anticancer immunogenicity. *Journal of Experimental Medicine*, 211(11):2231–2248, 2014.

[5] Lee P. Richman, Robert H. Vonderheide, and Andrew J. Rech. Neoantigen dissimilarity to the self-proteome predicts immunogenicity and response to immune checkpoint blockade. *Cell Systems*, 9(4):375–382.e4, 2019.

[6] Preeti Bais, Sandeep Namburi, Daniel M. Gatti, Xinyu Zhang, and Jeffrey H. Chuang. CloudNeo: a cloud pipeline for identifying patient-specific tumor neoantigens. *Bioinformatics*, 33(19):3110–3112, 2017.

[7] Jingcheng Wu, Wenzhe Wang, Jiucheng Zhang, Binbin Zhou, Wenyi Zhao, Zhixi Su, Xun Gu, Jian Wu, Zhan Zhou, and Shuqing Chen. DeepHLApan: a deep learning approach for neoantigen prediction considering both HLA-peptide binding and immunogenicity. *Frontiers in Immunology*, 10(November):1–11, 2019.

[8] Arjun A. Rao, Ada A. Madejska, Jacob Pfeil, Benedict Paten, Sofie R. Salama, and David Haussler. ProTECT—prediction of T-Cell epitopes for cancer therapy. *Frontiers in Immunology*, 11(November):1–11, 2020.

[9] Alex Rubinsteyn, Julia Kodysh, Isaac Hodes, Sebastien Mondet, Bulent Arman Aksoy, John P. Finnigan, Nina Bhardwaj, and Jeffrey Hammerbacher. Computational pipeline for the PGV-001 neoantigen vaccine trial. *Frontiers in Immunology*, 8(JAN):1–7, 2018.

[10] Jin Zhang, Elaine R. Mardis, and Christopher A. Maher. INTEGRATE-neo: a pipeline for personalized gene fusion neoantigen discovery. *Bioinformatics*, 33(4):555–557, 2017.

[11] Anne Mette Bjerregaard, Morten Nielsen, Sine Reker Hadrup, Zoltan Szallasi, and Aron Charles Eklund. MuPeXI: prediction of neo-epitopes from tumor sequencing data. *Cancer Immunology, Immunotherapy*, 66(9):1123–1130, 2017.

[12] Takanori Hasegawa, Shuto Hayashi, Eigo Shimizu, Shinichi Mizuno, Atsushi Niida, Rui Yamaguchi, Satoru Miyano, Hidewaki Nakagawa, and Seiya Imoto. Neoantimon: a multifunctional R package for identification of tumor-specific neoantigens. *Bioinformatics*, 36(18):4813–4816, 2020.

[13] Ana Carolina M.F. Coelho, André L. Fonseca, Danilo L. Martins, Paulo B.R. Lins, Lucas M. Da Cunha, and Sandro J. De Souza. NeoANT-HILL: an integrated tool for identification of potential neoantigens. *BMC Medical Genomics*, 13(1):1–8, 2020.

[14] Bo Wen, Kai Li, Yun Zhang, and Bing Zhang. Cancer neoantigen prioritization through sensitive and reliable proteogenomics analysis. *Nature Communications*, 11(1):1–14, 2020.

[15] Ting You Wang, Li Wang, Sk Kayum Alam, Luke H. Hoeppner, and Rendong Yang. ScanNeo: identifying indel-derived neoantigens using RNA-Seq data. *Bioinformatics*, 35(20):4159–4161, 2019.

[16] Zhan Zhou, Xingzheng Lyu, Jingcheng Wu, Xiaoyue Yang, Shanshan Wu, Jie Zhou, Xun Gu, Zhixi Su, and Shuqing Chen. TSNAD: an integrated software for cancer somatic mutation and tumour-specific neoantigen detection. *Royal Society Open Science*, 4(4), 2017.

[17] Elias Tappeiner, Francesca Finotello, Pornpimol Charoentong, Clemens Mayer, Dietmar Rieder, and Zlatko Trajanoski. TIminer: NGS data mining pipeline for cancer immunology and immunotherapy. *Bioinformatics*, 33(19):3140–3141, 2017.

[18] Ryan O. Schenck, Eszter Lakatos, Chandler Gatenbee, Trevor A. Graham, and Alexander R.A. Anderson. NeoPredPipe: high-throughput neoantigen prediction and recognition potential pipeline. *BMC Bioinformatics*, 20(1):1–6, 2019.

[19] Brendan Bulik-Sullivan, Jennifer Busby, Christine D. Palmer, Matthew J. Davis, Tyler Murphy, Andrew Clark, Michele Busby, Fujiko Duke, Aaron Yang, Lauren Young, Noelle C. Ojo, Kamilah Caldwell, Jesse Abhyankar, Thomas Boucher, Meghan G. Hart, Vladimir Makarov, Vincent Thomas De Montpreville, Olaf Mercier, Timothy A. Chan, Giorgio Scagliotti, Paolo Bironzo, Silvia Novello, Niki Karachaliou, Rafael Rosell, Ian Anderson, Nashat Gabrail, John Hrom, Chainarong Limvarapuss, Karin Choquette, Alexander Spira, Raphael Rousseau, Cynthia Voong, Naiyer A. Rizvi, Elie Fadel, Mark Frattini, Karin Jooss, Mojca Skoberne, Joshua Francis, and Roman Yelensky. Deep learning using tumor HLA peptide mass spectrometry datasets improves neoantigen identification. *Nature Biotechnology*, 37(1):55–71, 2019.

[20] Zhanbing Zhang, Chi Zhou, Lihua Tang, Yukang Gong, Zhiting Wei, Gongchen Zhang, Feng Wang, Qi Liu, and Jing Yu. ASNEO: identification of personalized alternative splicing based neoantigens with RNA-seq. *Aging*, 12(14):14633–14648, 2020.

[21] Mary A. Wood, Austin Nguyen, Adam J. Struck, Kyle Ellrott, Abhinav Nellore, and Reid F. Thompson. Neoepiscope improves neoepitope prediction with multivariant phasing. *Bioinformatics*, 36(3):713–720, 2020.

[22] Georgios Fotakis, DIetmar Rieder, Marlene Haider, Zlatko Trajanoski, and Francesca Finotello. NeoFuse: predicting fusion neoantigens from RNA sequencing data. *Bioinformatics*, 36(7):2260–2261, 2020.

[23] Françoise Baylis, Philip A Ewels, Alexander Peltzer, Sven Fillinger, Harshil Patel, Johannes Alneberg, Andreas Wilm, Maxime Ulysse Garcia, Paolo Di Tommaso, and Sven Nahnsen. The nf-core framework for community-curated bioinformatics pipelines. *Nature Biotechnology*, 38(3):276–278, 2020.

[24] Julia Kodysh and Alex Rubinsteyn. OpenVax: an open-source computational pipeline for cancer neoantigen prediction. In *Methods in Molecular Biology*, volume 2120, pages 147–160. 2020.

[25] Chunyu Liu, Yu Zhang, Xingxing Jian, Xiaoxiu Tan, Manman Lu, Jian Ouyang, Zhenhao Liu, Yuyu Li, Linfeng Xu, Lanming Chen, Yong Lin, and Lu Xie. ProGeo-Neo v2.0: a one-stop software for neoantigen prediction and filtering based on the proteogenomics strategy. *Genes*, 13(5), 2022.

[26] Alexander Rubinsteyn, Isaac Hodes, Julia Kodysh, and Jeffrey Hammerbacher. Vaxrank: a computational tool for designing personalized cancer vaccines. *bioRxiv*, 2018.

[27] Yunxia Tang, Yu Wang, Jiaqian Wang, Miao Li, Linmin Peng, Guochao Wei, Yixing Zhang, Jin Li, and Zhibo Gao. TruNeo: an integrated pipeline improves personalized true tumor neoantigen identification. *BMC Bioinformatics*, 21(1):1–16, 2020.

[28] Jasreet Hundal, Susanna Kiwala, Joshua McMichael, Christopher A. Miller, Huiming Xia, Alexander T. Wollam, Connor J. Liu, Sidi Zhao, Yang Yang Feng, Aaron P. Graubert, Amber Z. Wollam, Jonas Neichin, Megan Neveau, Jason Walker, William E. Gillanders, Elaine R. Mardis, Obi L. Griffith, and Malachi Griffith. PVACtools: a computational toolkit to identify and visualize cancer neoantigens. *Cancer Immunology Research*, 8(3):409–420, 2020.

[29] Zhiting Wei, Chi Zhou, Zhanbing Zhang, Ming Guan, Chao Zhang, Zhongmin Liu, and Qi Liu. The landscape of tumor fusion neoantigens: a pan-cancer analysis. *iScience*, 21:249–260, 2019.
